# Identification of Thrombosis-Related Genes in Patients with Advanced Gastric Cancer: Data from AGAMENON-SEOM Registry

**DOI:** 10.3390/biomedicines10010148

**Published:** 2022-01-11

**Authors:** David Zaragoza-Huesca, Pedro Garrido-Rodríguez, Paula Jiménez-Fonseca, Eva Martínez de Castro, Manuel Sánchez-Cánovas, Laura Visa, Ana Custodio, Ana Fernández-Montes, Julia Peñas-Martínez, Patricia Morales del Burgo, Javier Gallego, Ginés Luengo-Gil, Vicente Vicente, Irene Martínez-Martínez, Alberto Carmona-Bayonas

**Affiliations:** 1 Centro Regional de Hemodonación, Department of Haematology and Medical Oncology, Hospital General Universitario Morales Meseguer, University of Murcia, IMIB-Arrixaca, 30003 Murcia, Spain; davidzaragozahuesca5369@gmail.com (D.Z.-H.); pedro.garridor@outlook.es (P.G.-R.); manuelsanchezcanovas@gmail.com (M.S.-C.); julia.penas@um.es (J.P.-M.); gines.luengo@um.es (G.L.-G.); vicente.vicente@carm.es (V.V.); alberto.carmonabayonas@gmail.com (A.C.-B.); 2 Centro de Investigación Biomédica en Red de Enfermedades Raras, U-765-CIBERER, Instituto de Salud Carlos III (ISCIII), 28029 Madrid, Spain; 3 Department of Medical Oncology, Instituto de Investigación Sanitaria del Principado de Asturias—ISPA, Hospital Universitario Central de Asturias, 33011 Oviedo, Spain; palucaji@hotmail.com; 4 Department of Medical Oncology, Hospital Universitario Marqués de Valdecilla, 39008 Santander, Spain; eva.martinezde@scsalud.es; 5 Department of Medical Oncology, Hospital del Mar, 08003 Barcelona, Spain; LVisa@parcdesalutmar.cat; 6 Department of Medical Oncology, Hospital Universitario La Paz, CIBERONC CB16/12/00398, 28046 Madrid, Spain; anabcustodio@gmail.com; 7 Department of Medical Oncology, Complejo Hospitalario Universitario de Ourense, 32005 Ourense, Spain; afm1003@hotmail.com; 8 Department of Pathology, Hospital Universitario Central de Asturias, 33011 Oviedo, Spain; pmburgo@gmail.com; 9 Department of Medical Oncology, Hospital General Universitario de Elche, 03203 Elche, Spain; jgallego@umh.es

**Keywords:** advanced gastric adenocarcinoma, genetic factors, subtypes, stratification, venous thromboembolism

## Abstract

Advanced gastric cancer is one of the most thrombogenic neoplasms. However, genetic mechanisms underlying this complication remain obscure, and the molecular and histological heterogeneity of this neoplasm hinder the identification of thrombotic biomarkers. Therefore, our main objective was to identify genes related to thrombosis regardless of Lauren subtypes. Furthermore, in a secondary exploratory study, we seek to discover thrombosis-associated genes that were specific to each TCGA molecular subtype. We designed a nested case-control study using the cohort of the AGAMENON national advanced gastric cancer registry. Ninety-seven patients were selected—48 with and 49 without venous thromboembolism (using propensity score matching to adjust for confounding factors)—and a differential gene expression array stratified by Lauren histopathological subtypes was carried out in primary tumor samples. For the secondary objective, the aforementioned differential expression analysis was conducted for each TCGA group. Fifteen genes were determined to be associated with thrombosis with the same expression trend in both the intestinal and diffuse subtypes. In thrombotic subjects, *CRELD1*, *KCNH8*, *CRYGN*, *MAGEB16*, *SAA1*, *ARL11*, *CCDC169*, *TRMT61A*, *RIPPLY3* and *PLA2G6* were underexpressed (adjusted-*p* < 0.05), while *PRKD3*, *MIR5683*, *SDCBP*, *EPS8* and *CDC45* were overexpressed (adjusted-*p* < 0.05), and correlated, by logistic regression, with lower or higher thrombotic risk, respectively, in the overall cohort. In each TCGA molecular subtype, we identified a series of genes differentially expressed in thrombosis that appear to be subtype-specific. We have identified several genes associated with venous thromboembolism in advanced gastric cancer that are common to Lauren intestinal and diffuse subtypes. Should these genetic factors be validated in the future, they could be complemented with existing clinical models to bolster the ability to predict thrombotic risk in individuals with advanced gastric adenocarcinoma.

## 1. Introduction

Advanced gastric adenocarcinoma (AGA) is a deadly neoplasm, with a median survival of less than 12 months in most modern series [1]. Moreover, AGA is one of the most thrombogenic tumors [2,3], with an 8–24% cumulative incidence of venous thromboembolism (VTE) events [4,5,6,7,8]. Various studies have reported worse outcomes for patients with gastric cancer and VTE [6,8,9,10,11]. Thus, a recent analysis of the Spanish AGAMENON-SEOM gastric cancer registry (Number Clinical Trial (NCT) 04958720) estimated a cumulative 6-month incidence of VTE of 8.2% (95% Confidence Interval (CI), 7.1–9.5%), demonstrating that thromboses shorten overall survival (OS) with a time ratio of 0.56 (95% CI, 0.43–0.74) [8]. In the flexible competing risk model, the Khorana score, tumor burden and cisplatin-based regimens had variable effects over time (*p*-value < 0.05), with their effect diluted in 2–3 months. In contrast, the significant predictors that had a constant effect were signet ring cells (cumulative sub-hazard ratio (csHR) 1.47; 95% CI, 1.06–2.05) and primary thromboprophylaxis (csHR 0.43; 95% CI, 0.18–0.99) [8]. Per contra, the model was not particularly well-calibrated in the high-risk range, making it inaccurate as a practical predictive tool of thrombotic risk. This suggested that the addition of biomarkers or genetic variables might be necessary, the biological heterogeneity of AGA being the foremost impediment.

In the last decade, Lauren’s histopathological classification, which divides gastric tumors into intestinal (IT) and diffuse (DT) subtypes, has a clear genetic correlate, each morphological variety typifying a distinct entity [12,13,14]. These differences correlate with the influence each subtype exerts on hemostasis; for instance, gastric tumors with signet-ring cells, typical of diffuse subtype [13], are characterized by a proteome that is richer in proteins of the complement system [15]. This biological system interacts closely with coagulation [16]. Even more, the genomic and molecular classification reported by the Cancer Genome Atlas (TCGA) has further divided gastric cancer into four subtypes: Epstein–Barr virus (EBV)-positive, those with microsatellite instability (MSI), genetically stable (GS) and chromosomal instability (CIN) [17]. Oddly, the Lauren classification is partially reflected in these categories, which confirms the notion that each histopathological subtype represents a different molecular condition; whereas the GS subtype is enriched by DT tumors, CIN tumors coincide in part with ITs. In this context, the different gastric cancer subtypes can be contemplated as having idiosyncratic thrombogenic mechanisms that must be taken into account when looking for genetic factors involved in the etiology of thrombosis, particularly with an eye toward finding biomarkers to aid patients in the future.

Thus, the main objective in this study was to identify genes related to thrombosis irrespective of Lauren subtypes. To this end, we have designed a nested case-control study under the cohort of AGA formerly reported by our group [8]. A differential genetic expression array stratified by Lauren subtypes was performed on primary tumor biopsies from patients with thrombosis and controls. On the basis that each molecular subtype might induce specific thrombogenic mechanisms, we opted to perform an exploratory analysis based on the TCGA categories.

## 2. Materials and Methods

### 2.1. Patients and Study Design

The patient population assessed derived from the Spanish AGAMENON registry, that enlists the collaboration of 34 Spanish hospitals and one center in Chile and recruits consecutive cases of unresectable or metastatic, locally advanced adenocarcinoma of the stomach, gastroesophageal junction, or distal esophagus. The original clinical cohort comprised 2129 patients with 211 recorded thromboses during first line chemotherapy. The clinical details and baseline characteristics of this cohort and quality criteria, etc., have been reported extensively previously [8,14,18,19,20,21,22,23,24,25]. The basic eligibility criteria included individuals over the age of 18 years, with a confirmed histological diagnosis of gastric, gastroesophageal, or distal esophageal adenocarcinoma. Metastatic or locally advanced and unresectable tumors were further prerequisites. All the subjects had to be treated as per clinical practice with at least one cycle of polychemotherapy with regimens deemed acceptable in clinical guidelines. All the participants were followed until demise or for a minimum of six months.

A nested case-control study was designed from this cohort. Accordant with this design, the cases of VTE that occurred in a predefined cohort were identified; a specific number of matched controls were selected for each from those without the disease under scrutiny. This is an efficient design in exploratory studies such as this, insofar as it reduces the cost and time of the study compared to the full cohort approach [26]. A 1:1 ratio of cases-controls was chosen; both were defined as subjects from the entire cohort with or without VTE, respectively. A fixed sample size of 100 samples was decided on available tissue samples (half with and half without VTE) and participants were selected by means of propensity score matching (PSM). The aim in implementing PSM was for the clinical and therapy attributes to be comparable between cases and controls, such that thrombotic risk would be attributable to the differences in gene expression. PSM was performed based on treatment schedule, use of cisplatin, use of trastuzumab, Eastern Cooperative Oncology Group performance status (ECOG-PS), liver disease burden, number of metastatic sites, histological grade, tumors with signet ring cells, Khorana index, age, sex and prior presence of vascular disease. Individuals who had received thromboprophylaxis were excluded. The matched samples were obtained by nearest neighbor matching with a caliper width of 0.2. The standardized differences method was applied to assess the balance diagnostics [27]. In general, standardized differences less than 10% indicate a proper balance between baseline variables [28].

Finally, the 100 primary tumor biopsies were collected from six Spanish hospitals (Hospital General Universitario Morales Meseguer, Hospital Universitario Central de Asturias, Complejo Hospitalario Universitario de Ourense, Hospital Universitario Marqués de Valdecilla, Hospital del Mar and Hospital Universitario La Paz). Of these 100 samples, 3 were not processed due to poor tissue quality; 51 of the 97 remaining had Lauren intestinal histology (29 with and 22 without VTE) and 46 had diffuse histology (19 with and 27 without VTE). All of the procedures were executed in fulfillment of the ethical standards of the committee in charge of human experimentation (institutional and national) and with the Declaration of Helsinki 1964 and its subsequent versions. Informed consent was obtained from all patients prior to their inclusion in the study. The ethic committee in clinical research of the Hospital General Universitario José María Morales Meseguer approved the study (C.P.AGAMENON-C.I.EST:30/14, 26 November 2014).

### 2.2. RNA Isolation

The formaldehyde-fixed, paraffin-embedded patient samples were cut into 10-micra slices. The kit PureLink™ FFPE Total RNA Isolation (Invitrogen™, ThermoFisher Scientific, Waltham, MA, USA) was then used to isolate the RNA, following the manufacturer’s protocols. After isolation, the RNA was filtered to eliminate impurities, using centrifugal filtration units from the Amicon^®^ Ultra-0,5 mL kit (Merck, Darmstadt, Germany), following the supplier’s instructions. Finally, RNA concentration was measured with NanoDrop (ThermoFisher Scientific, Waltham, MA, USA) and the purified samples were stored at −80 °C.

### 2.3. Expression Array

The expression analysis was performed by means of the Clariom D human array (Affymetrix ThermoFisher Scientific, Waltham, MA, USA). Two ng of the total RNA were processed with the GeneChip WT Pico Reagent kit (Affymetrix ThermoFisher Scientific, Waltham, MA, USA), following the supplier’s instructions. The amount and quality of the resulting cDNAs were determined using NanoDrop 2000 (ThermoFisher Scientific, Waltham, MA, USA) and Bioanalyzer (Agilent Technologies, Santa Clara, CA, USA). Next, cDNAs were washed, fragmented, labelled and finally added to the hybridization mix using the GeneChip Hybridization, Wash and Stain kit (Affymetrix ThermoFisher Scientific, Waltham, MA, USA), following the manufacturer’s protocols. The resulting preparations were hybridized in the Clariom D human array and the results of the analysis were generated as Cell Intensity Data (CEL) files.

### 2.4. Sample Classification According to TCGA Subtypes

For this secondary aim, samples were classified according to TCGA subtypes: EBV, MSI, GS and CIN. This was done on the basis of a list of 80 overexpressed or underexpressed genes in each category according to the original analysis (see Table S1), which constitutes a reasonably accurate approach to the more complex multi-omic classification [17]. The criterion used to classify the samples was the relative overexpression of said genes, corresponding to values above the third quartile (Q3) plus 1.5 times the interquartile range (1.5 × IQR), or their relative underexpression, corresponding to levels below the first quartile (Q1) minus 1.5 × IQR. The normalized expression of the genes in the samples is displayed in Figure S1. This type of filtering is convenient when no expression controls are available; furthermore, this kind of criterion has already been used by other authors [29]. This modus operandi made it possible to unequivocally classify all the samples, with the exception of 13 samples that were imputed using anatomic location, gender, histopathological subtype, age and HER2 amplification (e.g., assigning DTs in young people to the GS category and HER2-positive tumors to the CIN category).

### 2.5. Statistical Analysis

The method of standardization used was the Gene Level-Signal Space Transformation-Robust Multi-Chip Analysis (SST-RMA) and the differential gene expression analysis was based on the ANOVA method adjusted to the Empirical Bayes Statistics for Differential Expression (eBayes) [30]. The differential gene expression between individuals with or without VTE was performed by means of Student’s *t* tests. For the main endpoint, analyses were stratified according to Lauren subtype (IT or DT). Within each subtype, genes with *p*-value < 0.05 and |Fold change| > 1 were selected, using the false discovery rate (FDR) < 10% criterion to adjust for multiplicity. Among the resulting genes, the final selection focused on those that maintained the same sense of expression between thrombosis patients and controls in both subtypes. The association of the expression of the resulting genes with thrombotic risk in the overall cohort was estimated by means of conditional logistic regression adjusted for histopathological subtype.

As regards the secondary aim, the differential gene expression analysis between thrombosis and controls was performed after stratifying by each TCGA subtype. Given the exploratory nature of this objective, genes yielding a *p*-value < 0.05, not adjusted for multiplicity, and a |Fold change| > 1.5 were selected. Descriptive heat maps showing differentially expressed genes within each TCGA subtype were represented. Moreover, for each TCGA category, these genes involvement in known biological routes was examined in an attempt to identify possible interactions with hemostasis. This was done by means of the open-source Reactome Pathway Database [31], using Analysis Tools. Analyses were performed with the Partek Genomic Suites v7.18.0723 and R v4.1 software, including the survival and oligo package [32,33].

## 3. Results

### 3.1. Patients

Table 1 lists patient baseline characteristics before and after PSM, which is effective in reducing absolute standardized differences for all categories, except a slightly higher percentage of males or tumors having >2 metastatic sites in cases with thrombosis.

### 3.2. Screening Differential Gene Expression Stratified by Histopathological Subtype

**Figure 1:**
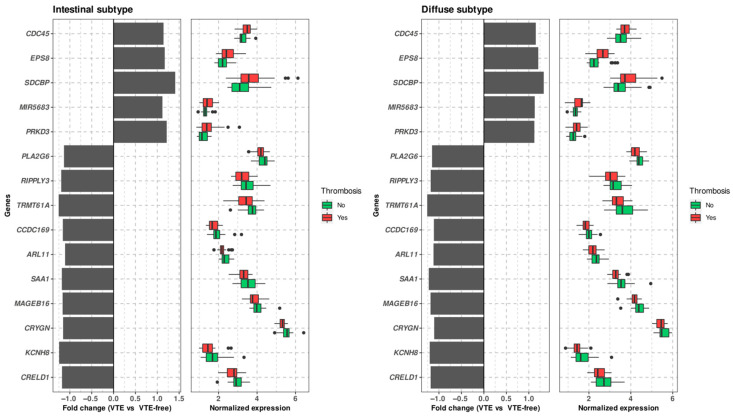
Selected genes from the stratified analysis by Lauren histopathological subtypes. Differential expression of these genes between individuals with thrombosis and controls is shown. These genes also had an adjusted *p*-value < 0.05 in both analyses. VTE: Venous thromboembolism.

To begin with, we assessed gene expression in individuals with or without VTE, factoring in Lauren subtype as stratification factor. The diffuse subtype samples comprised 27 controls and 19 cases with thrombosis, whereas the intestinal subtype consisted of 22 controls and 29 patients with thrombosis. The analysis uncovered 15 genes that were differentially expressed in both subtypes with the same expression trend between VTE patients and controls (Table 2 and Table 3). Ten of them were underexpressed in tissue samples from subjects with thrombosis: *CRELD1*, *KCNH8*, *CRYGN*, *MAGEB16*, *SAA1*, *ARL11*, *CCDC169*, *TRMT61A*, *RIPPLY3* and *PLA2G6*, whereas five were overexpressed: *PRKD3*, *MIR5683*, *SDCBP*, *EPS8* and *CDC45*. Figure 1 illustrates the differential expression and fold change between thrombotic patients and controls of the 15 genes in both the IT and DT subtypes.

### 3.3. Conditional Logistic Regression by Histopathology in the Overall Cohort

**Figure 2:**
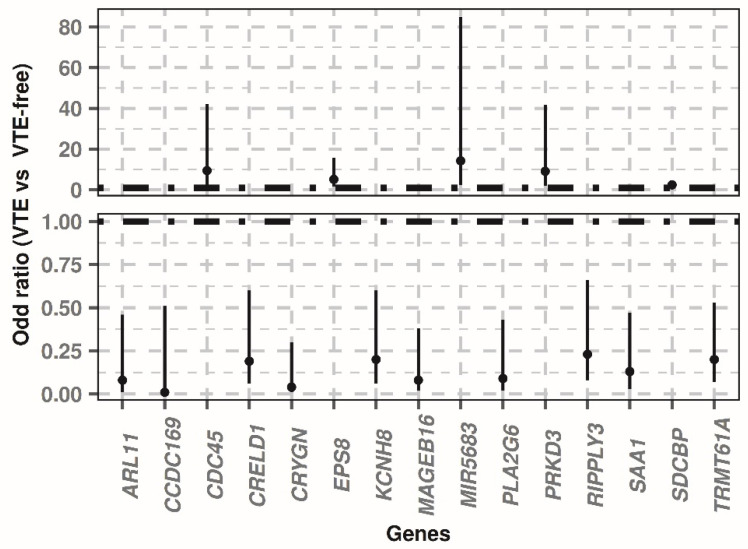
Odd ratios resulting from the conditional logistic regressions by histopathological stratum. The black and dashed line by dot–dash indicates the 1 value on both Y-axis sections. The odd ratios above 1 on this axis indicated that the greater the gene expression, the greater associated thrombotic risk in the overall cohort, whereas values of less than 1 indicated that the greater the expression, the lower the associated risk. In the 15 genes, the confidence intervals (vertical black solid lines) of the odd ratios do not cross the value of 1, which points to all the regressions being significant. VTE: Venous thromboembolism.

When we subjected the expression of the 15 previously-named genes to a conditional logistic regression by histopathological stratum to estimate their association with thrombotic risk in the entire cohort, we discovered that the 10 underexpressed genes in patients with VTE were significantly associated with a lower thrombotic risk as their expression increased (odd ratios VTE vs VTE-free < 1; *p*-value < 0.01), while the five overexpressed genes in patients with VTE correlated significantly with elevated thrombotic risk as their expression increased (odd ratios VTE vs VTE-free > 1; *p*-value < 0.05). Table 4 displays the *p*-value and odd ratios with respect to expression and thrombotic risk in the overall cohort for the logistic regression of each gene, in addition to the confidence interval of odd ratios. Figure 2 compares the odd ratios of the regressions carried out for each gene.

### 3.4. Classification of the Samples in the TCGA Categories and Differential Gene Expression Screening within Each Category

**Figure 3:**
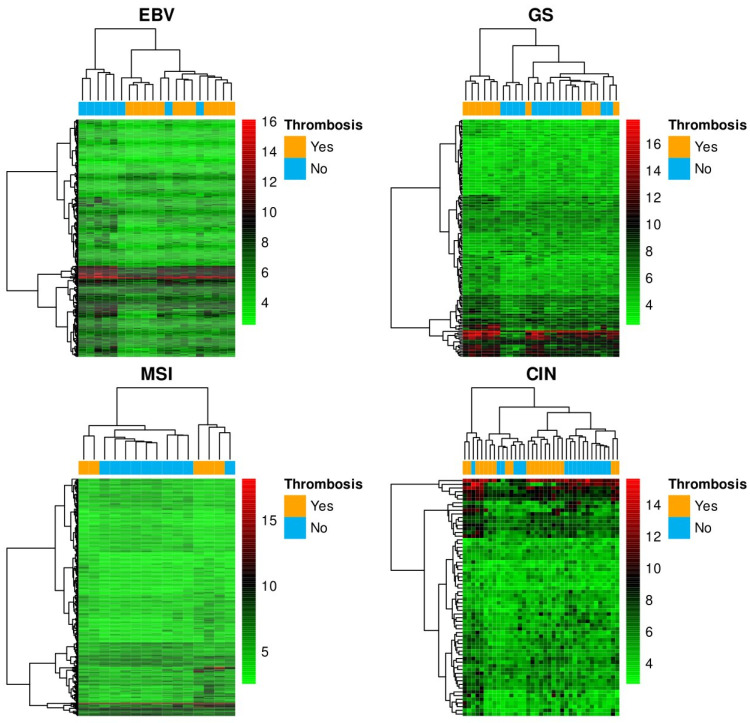
Descriptive heat maps of the differential gene expression between patients with thrombosis and controls within each TCGA category. The dendrogram on the left represents differentially expressed genes between individuals with thrombosis and controls, whereas the one on top represents the samples. In the rows corresponding to the expression of each gene, the shade of red indicates high relative expression with respect to the shade of green. EBV: Epstein–Barr associated tumors; GS: genetically stable tumors; MSI: tumors with microsatellite instability; CIN: chromosomally unstable tumors.

We classified all 97 samples into TCGA subtypes and, in the end, 20 tumors were EBV, 15 were MSI, 25 were GS and 37 were CIN subtype, that contained 12, 5, 11 and 20 VTEs, respectively. Figure 3 illustrates the descriptive heat maps of the differential gene expression between patients with VTE and controls in each category. In MSI tumors, 7 out of 452 differentially expressed genes (Table S2) were related to hemostatic pathways according to REACTOME (Table 5): DGKI, HBD, IGLV10-54, IGHA1, KIF25, GNAQ and RAP1B. As regards EBV tumors, 13 out of 409 differentially expressed genes between cases and controls (Table S3) were associated to hemostasis according to REACTOME (Table 5): ACP1, CEACAM3, EHD1, HISTH2H3A-C, IGKV3D-20, IGLV2-18, 6 kinesin genes and NHLRC2. In CIN tumors, 2 out of 64 differentially expressed genes in patients with VTE compared to controls (Table S4) were associated with hemostatic pathways (Table 5): F9 and LRRC16A. Finally, among GS tumors, 14 out of 154 differentially expressed genes (Table S5) were associated with hemostasis (Table 5): GNAS, PPP2R1B and 12 immunoglobulin genes.

## 4. Discussion

In this study, we have analyzed the genes that are differentially expressed in advanced gastric tumors with or without VTE to select those which are unconditioned by Lauren subtype differences. The tissue samples belong to the national AGAMENON-SEOM gastric cancer registry, with an 8.2% cumulative incidence of thrombosis and impact on survival end points [8]. The reason for conducting this analysis is the lack of detailed knowledge regarding the molecular mechanisms of thrombogenesis in this population, resulting in the absence of useful predictive biomarkers of thrombotic risk to complement clinical models. The inability to discern VTE means that, despite the reduction of thrombotic risk with thromboprophylaxis with a subhazard ratio 0.43 (95% CI, 0.18–0.99), the projection on the cumulative scale is modest [8].

The molecular heterogeneity of gastric cancer is the fundamental hurdle to investigating biological networks linked with thrombosis, to the extent to which each subtype could interact differentially with the hemostatic system. It has become clear in recent years that the Lauren histopathological subtypes comprise different biological entities, with disparate prognoses, pattern of dissemination and treatment response [13,14]. More recently, the TCGA has identified four molecular categories to stratify patients in clinical trials of targeted therapies [17].

This analysis has enabled us to identify genes involved in thrombosis that are common to both intestinal and diffuse gastric cancer subtypes. Taking into account these genes, SAA1, underexpressed in patients with VTE, is particularly salient. SAA1 codes for the serum protein amyloid A1, which interacts with multifarious proteins and receptors. SAA1 has been connected suppressing the microbial-induced inflammation and tissue damage [34], a protective action in gastric cancer, considering that it is often associated with infections, such as Helicobacter pylori infection [35]. Given this anti-inflammatory activity, SAA1 could contribute to avoid thrombosis, inasmuch as inflammatory mechanisms can lead to the development of thrombosis [36]. Nevertheless, there are also other studies that support this gene’s proinflammatory activity [37]; therefore, its involvement in the inflammation-thrombosis nexus remains subject to debate. The most conspicuous overexpressed genes in patients with VTE include PRKD3, EPS8 and MIR5683. PRKD3 has been correlated with gastric cancer progression through the activation of anaerobic glycolysis (Warburg effect) [38], a process that might also be linked to the appearance of venous thrombosis, since it has been reported that the erythrocytes in fresh venous thrombi, in comparison with normal blood, contained high levels of metabolites derived from anaerobic glycolysis, such as lactic acid [39]. EPS8 codes for a protein responsible for regulating blood vessel permeability. In this context, EPS8 expression has been reported to promote the internalization and ubiquitination of vascular endothelium-cadherin from the endothelial membrane [40], which diminishes its stabilizing function of junctions between endothelial cells. This phenomenon promotes increased vascular permeability and thereby facilitates the transmigration of leukocytes from the blood vessel lumina [41] that, as they accumulate on the vascular wall, damage the tissue that can cause procoagulant factors to be released to the lumina [42]. For its part, MIR5683 codes for a microRNA whose association with thrombosis has yet to be reported. Nonetheless, one potential target of this miRNA, according to the TargetScanHuman database, is the tissue factor pathway inhibitor (TFPI), a membrane bound or secreted protein by endothelial cells that inhibits the tissue Factor/FVIIa catalytic complex [43].

The reader must be mindful of the fact that our primary objective here was to dilucidate causal mechanisms implicated in thrombosis, more than to discern potential predictors that might be directly applicable. That being said, our results point toward these 15 genes as possible candidates for predictive factors; this must be validated in an independent cohort. In parallel, we assessed differential gene expression between cases with VTE and controls within each TCGA category and were able to identify genes linked to hemostasis that may be specific to each subtype.

Similar to our study, there are others that also focus on finding thrombosis-related genetic agents in cancer, although they do not emphasize stratifying for subtypes. For instance, in the project conducted by Ünlü et al., a series of genes and pathways related to thrombosis in the context of colorectal cancer were identified, whose role in inflammation and platelet function increase were highlighted [44]. Another example would be the study carried out by Sussman et al., that reported a series of differentially expressed genes in subjects with lung cancer who had suffered a venous thromboembolic event, and that underscored those pathways related with the complement, inflammation and the KRAS signaling [45].

Our article has certain limitations. To begin with, the nested case-control design from a complete cohort is efficient to the purpose of this translational study, enabling valid odd ratios to be gleaned. Nevertheless, this design means that the number of subjects included in certain subtypes may be inadequately represented with respect to a real-world cohort and that cumulative incidence rates in each molecular subgroup cannot be estimated directly. Secondly, categorizing patients into the TCGA classification is based on transcriptomic techniques and gene expression analysis, but fails to factor in the somatic alterations or methylation patterns, as in the TCGA study. While concordance with the multiomic classification in the original study is reasonable [17], the possibility of misclassification in a small percentage of cases is possible. Finally, these findings must be validated in an external cohort, so as to be able to identify individual biomarkers or molecular patterns capable of predicting thrombotic risk.

In conclusion, despite the vast molecular heterogeneity of gastric cancer, we have detected genes related to thrombosis present in both Lauren subtypes. On the other hand, our results also suggest that there may be thrombogenic mechanisms that are promoted by specific genes in each TCGA molecular subtype. Should these genetic factors be validated in the future, they could be complemented with existing clinical models to bolster the ability to predict thrombotic risk in individuals with AGA.

## Figures and Tables

**Table 1:** Baseline characteristics of patients with and without thrombosis. We show said characteristics in the cohort from AGAMENON registry before applying Propensity Score Matching, and in our selected cohort after applying such matching. Standardized differences or D* were applied to assess the balance diagnostics.

	Before PSM ^1^			After PSM ^1^		
Characteristics	Thrombosis	No Thrombosis	D* ^2^	Thrombosis	No Thrombosis	D* ^2^
	(*N* = 211)	(*N* = 1918)		(*N* = 48)	(*N* = 49)	
Median age (range), years	64 (20–89)	64 (22–85)	−0.08	62 (30–84)	60 (38–82)	−0.05
Sex: males, N (%)	146 (69.2)	1363 (71.1)	4.15	36 (75)	34 (69.4)	−12.5
HER2+ ^3^, N (%)	45 (21.3)	338 (17.6)	−9.35	11 (22.9)	10 (20.4)	−1.19
ECOG-PS ^4^						
0, N (%)	51 (24.2)	418 (21.8)	−5.70	11 (22.9)	11 (22.4)	−1.19
≥1, N (%)	160 (75.8)	1500 (78.2)	5.70	37 (77.0)	38 (77.5)	0.95
Number of metastatic sites: >2, N (%)	74 (35.1)	525 (27.4)	−16.8	20 (41.7)	17 (34.7)	−14.4
Histological grade,						
Poorly differentiated, N (%)	93 (43.6)	777 (40.5)	−6.28	25 (52.1)	26 (53.1)	2.00
Chemotherapy Regimen,						
Anthracicline-based, N (%)	48 (22.7)	401 (20.9)	−4.36	11 (22.9)	11 (22.4)	−1.19
Cisplatin-based, N (%)	57 (27.0)	379 (19.8)	−19.0	13 (27.1)	12 (24.5)	−5.94
Docetaxel-based, N (%)	21 (10.0)	224 (11.7)	5.46	7 (14.6)	8 (16.3)	4.07
Oxaliplatin-based, N (%)	69 (32.7)	756 (39.0)	13.1	11 (22.9	11 (22.4)	−1.19
Use of trastuzumab	42 (19.9)	289 (15.1)	−12.6	11 (22.9)	10 (20.4)	−6.07
Signet-ring cells	76 (36.0)	561 (29.2)	−14.5	20 (41.7)	20 (40.8)	−1.82
Charlson index (≥2)	283 (14.8)	33 (15.6)	2.22	7 (14.6)	6 (12.2)	−7.04

^1^ PSM: propensity score matching; ^2^ D*: standardized difference; ^3^ HER2+: patients with HER2 protein expression; ^4^ ECOG-PS: Eastern Cooperative Oncology Group performance status.

**Table 2:** Differentially expressed genes between patients with venous thromboembolism and controls, in the diffuse subtype.

Gene Name ^1^	Gene Symbol	Fold Change (VTE ^2^ vs. Control)	*p*-Value (VTE ^2^ vs. Control)	Adjusted *p*-Value (VTE ^2^ vs. Control)
Cysteine Rich with EGF Like Domains 1	CRELD1	−1.18471	0.048	0.049
Potassium Voltage-Gated Channel Subfamily H Member 8	KCNH8	−1.20454	0.029	0.048
Crystallin Gamma N	CRYGN	−1.10497	0.048	0.049
Melanoma-Associated Antigen B16	MAGEB16	−1.18731	0.006	0.048
Serum Amyloid A-1 Protein	SAA1	−1.2262	0.011	0.048
ADP Ribosylation Factor Like GTPase 11	ARL11	−1.12071	0.042	0.048
Coiled-Coil Domain Containing 169	CCDC169	−1.11201	0.040	0.048
TRNA Methyltransferase 61A	TRMT61A	−1.26067	0.023	0.048
Ripply Transcriptional Repressor 3	RIPPLY3	−1.18346	0.038	0.048
Phospholipase A2 Group VI	PLA2G6	−1.15315	0.017	0.048
Protein Kinase D3	PRKD3	1.12272	0.031	0.048
MicroRNA 5683	MIR5683	1.13071	0.015	0.048
Syndecan Binding Protein	SDCBP	1.33127	0.034	0.048
Epidermal Growth Factor Receptor Pathway Substrate 8	EPS8	1.20927	0.032	0.048
Cell Division Cycle 45	CDC45	1.15432	0.042	0.048

^1^ These 15 genes maintained their expression trend across thrombotic patients and controls in both Lauren subtypes; ^2^ VTE: Venous thromboembolism.

**Table 3:** Differentially expressed genes between patients with venous thromboembolism and controls, in the intestinal subtype.

Gene Name ^1^	Gene Symbol	Fold Change (VTE ^2^ vs. Control)	*p*-Value (VTE ^2^ vs. Control)	Adjusted *p*-Value (VTE ^2^ vs. Control)
Cysteine Rich with EGF Like Domains 1	CRELD1	−1.1796	0.028	0.041
Potassium Voltage-Gated Channel Subfamily H Member 8	KCNH8	−1.2452	0.024	0.041
Crystallin Gamma N	CRYGN	−1.1545	0.004	0.041
Melanoma-Associated Antigen B16	MAGEB16	−1.16573	0.022	0.041
Serum Amyloid A-1 Protein	SAA1	−1.18331	0.029	0.041
ADP Ribosylation Factor Like GTPase 11	ARL11	−1.10932	0.031	0.041
Coiled-Coil Domain Containing 169	CCDC169	−1.16057	0.027	0.041
TRNA Methyltransferase 61A	TRMT61A	−1.25583	0.012	0.041
Ripply Transcriptional Repressor 3	RIPPLY3	−1.19727	0.044	0.044
Phospholipase A2 Group VI	PLA2G6	−1.13422	0.032	0.041
Protein Kinase D3	PRKD3	1.21549	0.023	0.041
MicroRNA 5683	MIR5683	1.11792	0.044	0.044
Syndecan Binding Protein	SDCBP	1.41051	0.039	0.044
Epidermal Growth Factor Receptor Pathway Substrate 8	EPS8	1.17085	0.033	0.041
Cell Division Cycle 45	CDC45	1.14306	0.015	0.041

^1^ These 15 genes maintained their expression trend across thrombotic patients and controls in both Lauren subtypes; ^2^ VTE: Venous thromboembolism.

**Table 4:** Results of the conditional logistic regression by histopathological stratum.

			95% Confidence Interval ^3^	
Gene Expression	*p*-Value ^1^	Odd Ratio (VTE ^4^ vs. VTE-Free) ^2^	Lower	Upper
Expression of *CRELD1*	0.005	0.19	0.06	0.6
Expression of *KCNH8*	0.004	0.2	0.06	0.6
Expression of *CRYGN*	0.002	0.04	0.01	0.3
Expression of *MAGEB16*	0.002	0.08	0.02	0.38
Expression of *SAA1*	0.002	0.13	0.03	0.47
Expression of *ARL11*	0.005	0.08	0.01	0.46
Expression of *CCDC169*	0.006	0.01	0.02	0.51
Expression of *TRMT61A*	0.002	0.2	0.07	0.53
Expression of *RIPPLY3*	0.006	0.23	0.08	0.66
Expression of *PLA2G6*	0.003	0.09	0.02	0.43
Expression of *PRKD3*	0.004	9.11	1.98	41.8
Expression of *MIR5683*	0.004	14.25	2.4	84.89
Expression of *SDCBP*	0.006	2.44	1.29	4.6
Expression of *EPS8*	0.004	5.15	1.69	15.73
Expression of *CDC45*	0.003	9.45	2.12	42.04

^1^ The *p*-value of each regression is presented, as well as ^2^ the odd ratios that express the relation between the expression of each gene and thrombotic risk in the full cohort. Similarly, ^3^ the confidence interval (95%) of each odd ratio is shown. ^4^ VTE: Venous thromboembolism.

**Table 5:** Within each TCGA category, differentially expressed genes between patients with venous thromboembolism and controls that were associated to hemostatic pathways according to REACTOME software. Fold change quantifies differences in gene expression when comparing thrombotic patients respecting to controls. *p*-value indicates significance grade of gene expression differences between thrombotic cases and controls. Column on the right shows specific pathways related to hemostasis to which genes are associated, according to REACTOME software.

	^5^ ID	^6^ T Avg (log2)	^7^ N Avg (log2)	Fold Change	*p*-Value	Gene Symbol	Description	REACTOME Hemostasis
GS ^1^	TC2000007915.hg.1	11.85	8.96	7.41	0.046	*GNAS*	GNAS complex locus	Platelet homeostasis
	TC1400010444.hg.1	10.64	8.46	4.53	0.046	*IGHA1*	Immunoglobulin heavy constant alpha 1	Cell surface interactions at the vascular wall
	TC1400010798.hg.1	6.37	5.19	2.27	0.034	*IGHA2*	Immunoglobulin heavy constant alpha 2 (A2m marker)	Cell surface interactions at the vascular wall
	TSUnmapped00000647.hg.1	5.13	4.5	1.55	0.013	*IGKV1-17*	Immunoglobulin kappa variable 1-17 [Source:HGNC Symbol; Acc:HGNC:5733]	Cell surface interactions at the vascular wall
	TSUnmapped00000816.hg.1	5.32	4.44	1.84	0.001	*IGKV1-33*	Immunoglobulin kappa variable 1-33 [Source:HGNC Symbol; Acc:HGNC:5737]	Cell surface interactions at the vascular wall
	TSUnmapped00000665.hg.1	6.98	5.59	2.62	0.034	*IGKV3-20*	Immunoglobulin kappa variable 3-20 [Source:HGNC Symbol; Acc:HGNC:5817]	Cell surface interactions at the vascular wall
	TC2200009222.hg.1	9.73	7.75	3.94	0.020	*IGLC3*	Immunoglobulin lambda constant 3 (Kern-Oz+ marker)	Cell surface interactions at the vascular wall
	TC2200006821.hg.1	6.27	5.59	1.6	0.036	*IGLC6*	Immunoglobulin lambda constant 6 (Kern + Oz− marker, gene/pseudogene)	Cell surface interactions at the vascular wall
	TC2200009219.hg.1	7.62	6.09	2.89	0.040	*IGLC1; IGLC2; IGLV3-1*	Immunoglobulin lambda constant 1; immunoglobulin lambda constant 2; immunoglobulin lambda variable 3-1	Cell surface interactions at the vascular wall
	TC2200009214.hg.1	4.74	4.07	1.59	0.019	*IGLV2-18*	Immunoglobulin lambda variable 2-18	Cell surface interactions at the vascular wall
	TC0400010961.hg.1	8.16	6.01	4.44	0.038	*JCHAIN*	Joining chain of multimeric IgA and IgM	Cell surface interactions at the vascular wall
	TC1100012303.hg.1	3.86	4.5	−1.56	0.031	*PPP2R1B*	Protein phosphatase 2, regulatory subunit A, beta	Platelet homeostasis
CIN ^2^	TC0X00008578.hg.1	4.56	3.87	1.61	0.038	*F9*	Coagulation factor IX	Clotting cascade
	TC0600007231.hg.1	5.77	5.15	1.53	0.006	*LRRC16A*	Leucine rich repeat containing 16A	Factors involved in megakaryocyte development and platelet production
MSI ^3^	TC0700012731.hg.1	3.55	2.95	1.51	0.003	*DGKI*	Diacylglycerol kinase, iota	Platelet activation, signaling and aggregation
	TC1100009918.hg.1	5.63	6.43	−1.74	0.013	*HBD*	Hemoglobin, delta	Factors involved in megakaryocyte development and platelet production
	TC2200006766.hg.1	5.28	4.37	1.88	0.009	*IGLV10-54*	Immunoglobulin lambda variable 10-54	Cell surface interactions at the vascular wall
	TC1400010444.hg.1	10.49	7.82	6.35	0.038	*IGHA1*	Immunoglobulin heavy constant alpha 1	Cell surface interactions at the vascular Wall
	TC0600010241.hg.1	7.84	8.59	−1.68	0.020	*KIF25*	Kinesin family member 25	Factors involved in megakaryocyte development and platelet production
	TC0900010485.hg.1	6.28	3.3	7.88	0.002	*GNAQ*	Guanine nucleotide binding protein (G protein), q polypeptide	Signal amplification; Thrombin signalling through proteinase activated receptors
	TC1200008107.hg.1	5.12	4.11	2.01	0.046	*RAP1B*	RAP1B, member of RAS oncogene family	Platelet aggregation (Plug formation)
EBV ^4^	TC1900008161.hg.1	4.65	5.38	−1.66	0.008	*CEACAM3*	Carcinoembryonic antigen-related cell adhesion molecule 3	Cell surface interactions at the vascular wall
	TC1100011190.hg.1	6.28	7.03	−1.68	0.029	*EHD1*	EH domain containing 1	Factors involved in megakaryocyte development and platelet production
	TC0100015701.hg.1	3.79	4.4	−1.53	0.001	*HIST2H3A; HIST2H3C*	Histone cluster 2, H3a; histone cluster 2, H3c	Factors involved in megakaryocyte development and platelet production
	TC0200008393.hg.1	5.45	6.19	−1.66	0.026	*IGKV3D-20*	Immunoglobulin kappa variable 3D-20	Cell surface interactions at the vascular Wall
	TC2200009214.hg.1	4.16	4.82	−1.58	0.013	*IGLV2-18*	Immunoglobulin lambda variable 2-18	Cell surface interactions at the vascular Wall
	TC0300007223.hg.1	3.33	4.3	−1.96	0.0002	*KIF15*	Kinesin family member 15	Factors involved in megakaryocyte development and platelet production
	TC1100010418.hg.1	3.34	3.95	−1.53	0.001	*KIF18A*	Kinesin family member 18A	Factors involved in megakaryocyte development and platelet production
	TC0500008777.hg.1	4.39	5.08	−1.61	0.025	*KIF20A*	Kinesin family member 20A	Factors involved in megakaryocyte development and platelet production
	TC1600007425.hg.1	3.23	3.9	−1.59	0.001	*KIF22*	Kinesin family member 22	Factors involved in megakaryocyte development and platelet production
	TC1500007699.hg.1	4.36	5.03	−1.59	0.038	*KIF23*	Kinesin family member 23	Factors involved in megakaryocyte development and platelet production
	TC0900010582.hg.1	3.63	4.33	−1.62	0.010	*KIF27*	Kinesin family member 27	Factors involved in megakaryocyte development and platelet production
	TC1000008961.hg.1	5.91	6.62	−1.64	0.010	*NHLRC2*	NHL repeat containing 2	Platelet activation, signaling and aggregation
	TC0200006440.hg.1	4.67	6.37	−3.24	0.004	*ACP1*	Acid phosphatase 1, soluble	Factors involved in development and platelet production

^1^ GS: genetically stable tumors; ^2^ CIN: chromosomal instability tumors; ^3^ MSI: tumors with microsatellite instability; ^4^ EBV: Epstein–Barr associated tumors; ^5^ ID: Transcript identifier from Clariom D Human array; ^6^ T Avg (log2): Logarithm to base 2 of expression levels average among patients with thrombosis; ^7^ N Avg (log2): Logarithm to base 2 of expression levels average among patients without thrombosis.

## Supplementary Materials

The following supporting information can be downloaded at: https://www.mdpi.com/article/10.3390/biomedicines10010148/s1, Table S1: Overexpressed and underexpressed genes in each gastric cancer molecular category according to TCGA study. Overexpression and underexpression of 80 genes were characteristics of the different categories. Figure S1: Normalized expression of genes from TCGA project in the samples from the study. Table S2: Differentially expressed genes between patients with venous thromboembolism and controls in microsatellite instability tumors. Table S3: Differentially expressed genes between patients with venous thromboembolism and controls in Epstein-Barr virus-positive tumors. Table S4: Differentially expressed genes between patients with venous thromboembolism and controls in chromosomal instability tumors. Table S5: Differentially expressed genes between patients with venous thromboembolism and controls in genomically stable tumors.
