# Supplementary material for: Identification of Thrombosis-Related Genes in Patients with Advanced Gastric Cancer: Data from AGAMENON-SEOM Registry
Source: Biomedicines. 2022 Jan 11;10(1):148. doi: 10.3390/biomedicines10010148 (PMC8773420; doi:10.3390/biomedicines10010148)
Supplement: Supplementary file 1 [file biomedicines-10-00148-s001.zip › biomedicines-1533227-supplementary/Table S1.pdf]

Table S1.

Gene	Expression	Category
ACTG2	Over	GS
APOD	Under	MSI
AQP5	Over	CIN
ARID1A	Under	EBV
	Under	MSI
AURKA	Under	GS
BPIFB1	Under	MSI
ERBB3	Over	MSI
C7	Over	GS
CAMK2N2	Over	GS
CCL18	Over	MSI
CCL20	Under	GS
CCL5	Under	CIN
CCND2	Under	CIN
CD274	Over	EBV
CDH17	Under	EBV
CDKN2A	Over	CIN
	Under	EBV
CLDN3	Under	EBV
CNN1	Over	GS
CST1	Under	GS
CXCL1	Over	MSI

Gene	Expression	Category
CXCL10	Over	EBV
CXCL11	Over	EBV
CXCL13	Under	CIN
CXCL14	Under	MSI
CXCL17	Over	EBV
CXCL9	Over	EBV
	Under	CIN
IDO1	Over	EBV
DES	Over	GS
DKK1	Over	EBV
EGFR	Over	CIN
ERBB2	Over	EBV
F5	Over	CIN
FAM3B	Over	CIN
FGFR2	Over	CIN
FUT3	Under	EBV
GABRP	Under	CIN
GPA33	Over	MSI
GZMA	Under	CIN
GZMK	Under	CIN
HABP2	Under	MSI
HOXC10	Over	CIN

Gene	Expression	Category
B2M	Under	MSI
HLA-B	Under	MSI
ITGB1	Over	GS
ITGB3	Over	GS
JAK2	Over	EBV
KLK6	Over	CIN
KRT7	Over	CIN
	Under	EBV
MET	Over	CIN
MIA	Over	EBV
MLH1	Under	MSI
MMP1	Under	GS
MMP12	Over	MSI
	Under	GS
MUC2	Over	MSI
MUC3A	Under	EBV
MUC6	Over	MSI
MYH11	Over	GS
NKG7	Under	CIN
OGN	Over	GS
PCSK1N	Over	CIN
	Under	MSI
PDCD1LG2	Over	EBV

Gene	Expression	Category
PGC	Under	MSI
PI3	Under	GS
PPP1R1B	Over	MSI
	Under	EBV
PTEN	Under	EBV
REG4	Under	EBV
RPL22L1	Over	MSI
	Under	CIN
SCNN1A	Under	EBV
SMAD4	Under	EBV
ALDH1A1	Under	MSI
SYNM	Over	GS
SYT13	Under	MSI
TFF3	Under	EBV
THBS4	Over	GS
TP53	Over	CIN
TPX2	Under	GS
TRIB3	Under	GS
UBE2C	Under	GS
UGT2A3	Over	EBV
WFDC2	Over	CIN
	Under	EBV
	Under	MSI
