# Supplementary material for: Identification of Thrombosis-Related Genes in Patients with Advanced Gastric Cancer: Data from AGAMENON-SEOM Registry
Source: Biomedicines. 2022 Jan 11;10(1):148. doi: 10.3390/biomedicines10010148 (PMC8773420; doi:10.3390/biomedicines10010148)
Supplement: Supplementary file 1 [file biomedicines-10-00148-s001.zip › biomedicines-1533227-supplementary/Table S2.pdf]

Table S2.

ID	T Avg (log2)	N Avg (log2)	Fold Change	P-value	Gene Symbol	Description
TC1500008043.hg.1	5.3	4.61	1.61	0.001	*ABHD17C*	Abhydrolase domain containing 17C
TC1000011904.hg.1	4.77	3.31	2.75	0.001	*ABLIM1*	Actin binding LIM protein 1
TC0300013635.hg.1	6.33	5.35	1.97	0.024	*ACAP2*	ArfGAP with coiled-coil, ankyrin repeat and PH domains 2
TC0200009596.hg.1	7.98	7.25	1.65	0.047	*ACVR2A*	Activin A receptor type IIA
TC1500007056.hg.1	4.75	3.64	2.16	0.002	*ADAL*	Adenosine deaminase-like
TC0100015921.hg.1	5.01	3.73	2.43	0.018	*ADAR*	Adenosine deaminase, RNA-specific
TC0600009669.hg.1	6.96	4.14	7.06	0.011	*ADGRG6*	Adhesion G protein-coupled receptor G6
TC0400011409.hg.1	4.5	3.61	1.85	0.050	*ADH1C*	Alcohol dehydrogenase 1C (class I), gamma polypeptide
TC0100006486.hg.1	4.3	3.7	1.51	0.010	*AGRN*	Agrin
TC0X00008264.hg.1	3.29	4.33	-2.06	0.003	*AKAP14*	A kinase (PRKA) anchor protein 14
TC1900010696.hg.1	4.52	3.66	1.81	0.033	*AKT2*	V-akt murine thymoma viral oncogene homolog 2
TC0200014934.hg.1	3.53	2.94	1.51	0.006	*ALDH7A1P2*	Aldehyde dehydrogenase 7 family member A1 pseudogene 2 [Source:HGNC Symbol;Acc:HGNC:879]
TC1100011739.hg.1	5.07	4.15	1.89	0.029	*ALG8*	ALG8, alpha-1,3-glucosyltransferase
TC1700007102.hg.1	4.25	3.41	1.79	0.007	*ALKBH5*	AlkB homolog 5, RNA demethylase
TC0100018249.hg.1	5.39	6.27	-1.84	0.029	*AMY2B; ACTG1P4*	Amylase, alpha 2B (pancreatic); actin gamma 1 pseudogene 4
TC0200016464.hg.1	4.78	3.91	1.83	0.002	*APLF*	Aprataxin and PNKP like factor
TC0400009024.hg.1	5.51	4.59	1.89	0.006	*ARFIP1*	ADP-ribosylation factor interacting protein 1
TC1000010980.hg.1	4.66	3.99	1.6	0.049	*ASCC1*	Activating signal cointegrator 1 complex subunit 1
TC0700011876.hg.1	6.27	7.03	-1.69	0.046	*ASNS*	Asparagine synthetase (glutamine-hydrolyzing)
TC0200011965.hg.1	4.33	3.6	1.66	0.002	*ATAD2B*	ATPase family, AAA domain containing 2B
TC0600012731.hg.1	4.69	3.77	1.89	0.030	*ATG5*	Autophagy related 5
TC0500012666.hg.1	4.58	3.78	1.74	0.023	*ATP10B*	ATPase, class V, type 10B
TC0X00010966.hg.1	4.69	3.85	1.79	0.008	*ATP11C*	ATPase, class VI, type 11C
TC1900010482.hg.1	3.84	4.44	-1.51	0.048	*ATP4A*	ATPase, H+/K+ exchanging, alpha polypeptide
TC0900010071.hg.1	6.26	5.41	1.81	0.004	*ATP5A1P8*	ATP synthase, H+ transporting, mitochondrial F1 complex, alpha subunit 1 pseudogene 8 [Source:HGNC Symbol;Acc:HGNC:37666]
TC1300006699.hg.1	4.02	3.2	1.76	0.013	*ATP5EP2*	ATP synthase, H+ transporting, mitochondrial F1 complex, epsilon subunit

						pseudogene 2
TC0300012670.hg.1	4.58	5.18	-1.52	0.047	*ATR*	ATR serine/threonine kinase
TC1200006445.hg.1	4.52	3.37	2.22	0.041	*B4GALNT3*	Beta-1,4-N-acetyl-galactosaminyl transferase 3
TC2100007198.hg.1	4.97	4.38	1.5	0.034	*BACE2*	Beta-site APP-cleaving enzyme 2
TC1300007587.hg.1	4.07	3.32	1.69	0.021	*BCAS2P3*	Breast carcinoma amplified sequence 2 pseudogene 3 [Source:HGNC Symbol;Acc:HGNC:39596]
TC0100009788.hg.1	6.56	5.79	1.71	0.010	*BCL9*	B-cell CLL/lymphoma 9
TC0400012952.hg.1	3.9	3.27	1.55	0.021	*BDH2*	3-hydroxybutyrate dehydrogenase, type 2
TC1000012522.hg.1	7.26	6.32	1.92	0.020	*BEND7*	BEN domain containing 7
TC1900010105.hg.1	6.07	6.75	-1.6	0.039	*BNIP3P19*	BCL2/adenovirus E1B 19kDa interacting protein 3 pseudogene 19 [Source:HGNC Symbol;Acc:HGNC:49699]
TC1900010183.hg.1	4.19	4.79	-1.52	0.047	*BNIP3P32*	BCL2/adenovirus E1B 19kDa interacting protein 3 pseudogene 32 [Source:HGNC Symbol;Acc:HGNC:49712]
TC0300007303.hg.1	5.54	4.89	1.57	0.017	*BOLA2P2*	BolA family member 2 pseudogene 2 [Source:HGNC Symbol;Acc:HGNC:51438]
TSUnmapped00000266.hg.1	3.77	3.17	1.51	0.008	*BORCS5*	BLOC-1 related complex subunit 5
TC2100008165.hg.1	4.9	4.12	1.72	0.029	*BRWD1*	Bromodomain and WD repeat domain containing 1
TC1100012418.hg.1	4.23	4.84	-1.53	0.038	*BUD13*	BUD13 homolog
TC0800010086.hg.1	3.83	3.22	1.53	0.016	*BUD31P1*	BUD31 homolog pseudogene 1 [Source:HGNC Symbol;Acc:HGNC:51561]
TC1800006891.hg.1	4.27	3.68	1.5	0.036	*C18orf8*	Chromosome 18 open reading frame 8
TC0400012821.hg.1	3.71	4.38	-1.59	0.049	*C4orf22*	Chromosome 4 open reading frame 22
TC0800010575.hg.1	3.52	4.33	-1.75	0.035	*CA8*	Carbonic anhydrase VIII
TC0200012159.hg.1	4.8	3.89	1.87	0.016	*CAPN13*	Calpain 13
TC0100017449.hg.1	7.38	6.67	1.64	0.031	*CAPN8*	Calpain 8
TC0100014573.hg.1	4.08	4.71	-1.55	0.047	*CASP3P1*	Caspase 3 pseudogene 1 [Source:HGNC Symbol;Acc:HGNC:43596]
TC0400006938.hg.1	6.18	5.15	2.04	0.026	*CC2D2A*	Coiled-coil and C2 domain containing 2A
TC0600014167.hg.1	5	4.16	1.8	0.019	*CCDC162P*	Coiled-coil domain containing 162, pseudogene
TC0300007557.hg.1	4.6	5.63	-2.04	0.044	*CCDC66*	Coiled-coil domain containing 66
TC1600010598.hg.1	4.25	5.06	-1.76	0.041	*CCDC79*	Coiled-coil domain containing 79
TC0900009914.hg.1	8.99	9.58	-1.51	0.019	*CCL27*	Chemokine (C-C motif) ligand 27
TC2100007854.hg.1	4.65	3.51	2.2	3.01E-05	*CCT8*	Chaperonin containing TCP1, subunit 8 (theta)
TC1100009202.hg.1	3.93	4.54	-1.52	0.004	*CD3G*	CD3g molecule, gamma (CD3-TCR complex)
TC0600012452.hg.1	5.2	4.22	1.98	1E-04	*CEP162*	Centrosomal protein 162kDa
TC1500008053	3.13	3.83	-1.63	0.022	*CFAP161*	Cilia and flagella associated protein 161

.hg.1						
TC0100011064 .hg.1	5.18	4.2	1.98	0.016	*CFH*	Complement factor H
TC0200014990 .hg.1	5.03	4.37	1.58	0.041	*CIR1*	Corepressor interacting with RBPJ, 1
TC0500012555 .hg.1	5.68	6.46	-1.71	0.035	*CIR1P1*	Corepressor interacting with RBPJ, 1 pseudogene 1 [Source:HGNC Symbol;Acc:HGNC:44011]
TC1500007067 .hg.1	6.8	5.16	3.12	0.021	*CKMT1A*	Creatine kinase, mitochondrial 1A
TC0100015952 .hg.1	4.9	4.04	1.81	0.024	*CLK2*	CDC like kinase 2
TC0500009856 .hg.1	5.27	4.26	2.02	0.028	*CLPTM1L*	CLPTM1-like
TC0100016748 .hg.1	4.91	4.12	1.73	0.013	*CLPTM1LP1*	CLPTM1L pseudogene 1 [Source:HGNC Symbol;Acc:HGNC:49023]
TC0100012816 .hg.1	5.16	4.02	2.2	0.030	*CLSTN1*	Calsyntenin 1
TC0400010614 .hg.1	3.27	4.03	-1.68	0.013	*CNGA1*	Cyclic nucleotide gated channel alpha 1
TC1700010327 .hg.1	5.5	4.51	1.99	0.034	*COPRS*	Coordinator of PRMT5, differentiation stimulator
TC0900010969 .hg.1	4.88	3.77	2.15	4E-04	*CORO2A*	Coronin, actin binding protein, 2A
TC0600014345 .hg.1	4.81	5.54	-1.66	0.010	*COX5BP2*	Cytochrome c oxidase subunit Vb pseudogene 2 [Source:HGNC Symbol;Acc:HGNC:2271]
TC0800011059 .hg.1	3.8	4.39	-1.51	0.032	*COX6B1P6*	Cytochrome c oxidase subunit VIb polypeptide 1 (ubiquitous) pseudogene 6 [Source:HGNC Symbol;Acc:HGNC:37676]
TC0800008300 .hg.1	5.01	4.41	1.52	0.025	*CPQ*	Carboxypeptidase Q
TC0X00008754 .hg.1	4.83	5.42	-1.51	0.020	*CSAG3*	CSAG family, member 3
TC1000010468 .hg.1	4.17	3.29	1.85	0.041	*CXCL12*	Chemokine (C-X-C motif) ligand 12
TC0600014148 .hg.1	4.02	4.83	-1.76	0.022	*CYB5R4*	Cytochrome b5 reductase 4
TC0300010227 .hg.1	3.67	4.28	-1.53	0.024	*CYCSP11*	Cytochrome c, somatic pseudogene 11 [Source:HGNC Symbol;Acc:HGNC:24385]
TC1200008364 .hg.1	3.3	4.15	-1.79	0.01	*CYCSP30*	Cytochrome c, somatic pseudogene 30
TC0900008277 .hg.1	4.77	4.11	1.59	0.017	*CYLC2*	Cylicin, basic protein of sperm head cytoskeleton 2
TC0500008849 .hg.1	5.52	4.41	2.15	0.033	*CYSTM1*	Cysteine-rich transmembrane module containing 1
TC0Y00007219 .hg.1	4.5	3.46	2.05	0.001	*DAZ4; DAZ1*	Deleted in azoospermia 4; deleted in azoospermia 1
TC1400009538 .hg.1	4.11	3.4	1.64	4E-04	*DCAF5*	DDB1 and CUL4 associated factor 5
TC1800007340 .hg.1	4.04	4.89	-1.8	0.009	*DCC*	DCC netrin 1 receptor
TC0200006540 .hg.1	3.83	4.6	-1.7	0.014	*DCDC2C*	Doublecortin domain containing 2C
TC1100008991 .hg.1	4.88	5.96	-2.1	0.028	*DDX10*	DEAD (Asp-Glu-Ala-Asp) box polypeptide 10
TC2000007072 .hg.1	4.36	3.77	1.5	0.049	*DEFB123*	Defensin, beta 123
TC2000008118 .hg.1	4.89	4.14	1.69	0.016	*DEFB128*	Defensin, beta 128
TC0700012731	3.55	2.95	1.51	0.003	*DGKI*	Diacylglycerol kinase, iota

.hg.1						
TC2200008704 .hg.1	3.92	4.7	-1.72	0.004	*DMC1*	DNA meiotic recombinase 1
TC0100011755 .hg.1	4.5	3.83	1.59	0.036	*DNAH14*	Dynein, axonemal, heavy chain 14
TC1500010067 .hg.1	6.13	6.83	-1.63	0.029	*DNM1P34*	Dynamin 1 pseudogene 34
TC1000008539 .hg.1	3.64	4.37	-1.66	0.024	*DNTT*	DNA nucleotidylexotransferase
TC1500009797 .hg.1	5.48	4.29	2.27	0.013	*DPP8*	Dipeptidyl-peptidase 8
TC1400006926 .hg.1	3.99	4.68	-1.62	0.023	*DPPA3P2*	Developmental pluripotency associated 3 pseudogene 2
TC1800008952 .hg.1	4.29	3.57	1.65	0.022	*DSEL*	Dermatan sulfate epimerase-like
TC1500010740 .hg.1	6.06	4.31	3.36	0.002	*DTWD1*	DTW domain containing 1
TC1100007729 .hg.1	4.77	3.82	1.92	0.028	*DTX4*	Deltex 4, E3 ubiquitin ligase
TC1000008891 .hg.1	6.09	5.47	1.54	0.047	*DUSP5*	Dual specificity phosphatase 5
TC0300009252 .hg.1	2.99	3.62	-1.54	0.049	*DYNLL1P5*	Dynein, light chain, LC8-type 1 pseudogene 5 [Source:HGNC Symbol;Acc:HGNC:49656]
TC0300009690 .hg.1	4.03	4.81	-1.72	0.041	*EIF2B5*	Eukaryotic translation initiation factor 2B, subunit 5 epsilon, 82kDa
TC0600012072 .hg.1	6	4.66	2.54	0.008	*ELOVL5*	ELOVL fatty acid elongase 5
TC0400007978 .hg.1	6.2	7.23	-2.04	0.039	*ENOPH1*	Enolase-phosphatase 1
TC0600013165 .hg.1	5.36	4.64	1.65	0.020	*EPB41L2*	Erythrocyte membrane protein band 4.1-like 2
TC0900011160 .hg.1	5.47	4.87	1.51	0.026	*EPB41L4B*	Erythrocyte membrane protein band 4.1 like 4B
TC1200010038 .hg.1	5.73	4.58	2.23	0.043	*EPS8*	Epidermal growth factor receptor pathway substrate 8
TC1300008760 .hg.1	6.55	4.73	3.53	0.049	*EPSTI1*	Epithelial stromal interaction 1 (breast)
TC1900008727 .hg.1	3.74	3.15	1.51	0.001	*ERVV-2*	Endogenous retrovirus group V, member 2
TC0800008263 .hg.1	4.32	3.49	1.77	0.004	*ESRP1*	Epithelial splicing regulatory protein 1
TC0200013046 .hg.1	4.27	3.42	1.8	0.011	*EXOC6B*	Exocyst complex component 6B
TC1600010861 .hg.1	5.03	3.38	3.13	0.002	*FA2H*	Fatty acid 2-hydroxylase
TC0X00011399 .hg.1	4.14	3.43	1.63	0.022	*FAM127B*	Family with sequence similarity 127, member B
TC0200014691 .hg.1	3.91	4.57	-1.59	0.010	*FAM133DP*	Family with sequence similarity 133, member A pseudogene
TC0X00009737 .hg.1	6.44	4.76	3.2	0.031	*FAM156A*	Family with sequence similarity 156, member A
TC0500013220 .hg.1	3.72	4.42	-1.62	0.024	*FAM170A*	Family with sequence similarity 170, member A
TC1500010929 .hg.1	5.91	5.11	1.73	0.049	*FAM174B*	Family with sequence similarity 174, member B
TC1000009217 .hg.1	4.6	3.35	2.37	0.039	*FAM175B*	Family with sequence similarity 175, member B
TC1400010594 .hg.1	4.07	3.31	1.7	0.001	*FAM177A1*	Family with sequence similarity 177, member A1
TC0800012272 .hg.1	4.01	3.23	1.72	0.032	*FAM66A*	Family with sequence similarity 66, member A

TC0500010019 .hg.1	4.3	5.03	-1.66	0.046	*FASTKD3*	FAST kinase domains 3
TC1200009489 .hg.1	4.21	3.6	1.53	0.045	*FBRSL1*	Fibrosin-like 1
TC1100013021 .hg.1	5.23	5.89	-1.58	0.008	*FEN1*	Flap structure-specific endonuclease 1
TC0500010651 .hg.1	3.97	4.73	-1.7	0.044	*FGF10*	Fibroblast growth factor 10
TC0700011050 .hg.1	4.29	3.71	1.5	0.025	*FIGNL1*	Fidgetin-like 1
TC1100013030 .hg.1	4.21	3.55	1.58	0.012	*FLRT1*	Fibronectin leucine rich transmembrane protein 1
TSUnmapped00000529.hg.1	5.35	4.51	1.79	0.020	*FMN1*	Formin 1
TC0X00006616 .hg.1	3.65	3	1.57	0.003	*FRMPD4*	FERM and PDZ domain containing 4
TC0100013305 .hg.1	4.47	3.7	1.71	0.001	*FUCA1*	Fucosidase, alpha-L- 1, tissue
TC0200010473 .hg.1	3.27	3.86	-1.5	0.015	*FZD7*	Frizzled class receptor 7
TC0600006918 .hg.1	5.51	4.64	1.82	0.047	*GCNT2*	Glucosaminyl (N-acetyl) transferase 2, I-branching enzyme (I blood group)
TC1600011573 .hg.1	3.79	4.8	-2.01	0.016	*GCSH*	Glycine cleavage system protein H (aminomethyl carrier)
TC0600008324 .hg.1	4.1	3.52	1.5	0.014	*GFRAL*	GDNF family receptor alpha like
TC1100010991 .hg.1	4.16	3.4	1.69	0.011	*GIF*	Gastric intrinsic factor (vitamin B synthesis)
TC0700013338 .hg.1	3.31	4.22	-1.88	1E-04	*GLCCI1*	Glucocorticoid induced 1
TC0900010485 .hg.1	6.28	3.3	7.88	0.002	*GNAQ*	Guanine nucleotide binding protein (G protein), q polypeptide
TC0300007044 .hg.1	5.73	6.48	-1.68	0.034	*GOLGA4*	Golgin A4
TC0200009938 .hg.1	6.5	5.46	2.06	0.035	*GORASP2*	Golgi reassembly stacking protein 2
TC1200006896 .hg.1	9	7.01	3.97	0.032	*GPRC5A; MIR614*	G protein-coupled receptor, class C, group 5, member A; microRNA 614
TC1200011140 .hg.1	4.05	4.68	-1.54	0.014	*GRIP1*	Glutamate receptor interacting protein 1
TC0700012490 .hg.1	3.82	3.22	1.51	0.005	*GRM8*	Glutamate receptor, metabotropic 8
TC1300009423 .hg.1	4.54	5.21	-1.59	0.041	*GRPEL2P1*	GrpE-like 2, mitochondrial (E. coli) pseudogene 1 [Source:HGNC Symbol;Acc:HGNC:41969]
TC0700011567 .hg.1	4.86	3.7	2.24	0.014	*GTF2IP7; AC005077.9*	General transcription factor IIi pseudogene 7 [Source:HGNC Symbol;Acc:HGNC:51720]
TC0100008422 .hg.1	4.18	4.8	-1.54	0.009	*GYG1P3*	Glycogenin 1 pseudogene 3 [Source:HGNC Symbol;Acc:HGNC:39711]
TC0500013237 .hg.1	5.66	4.63	2.04	0.022	*HARS2*	Histidyl-tRNA synthetase 2, mitochondrial
TC1500007035 .hg.1	4.48	3.74	1.68	0.005	*HAUS2*	HAUS augmin like complex subunit 2
TC1100009918 .hg.1	5.63	6.43	-1.74	0.013	*HBD*	Hemoglobin, delta
TC1500006675 .hg.1	9.39	7.86	2.89	0.047	*HERC2P9*	Hect domain and RLD 2 pseudogene 9
TC0400008105 .hg.1	5.46	6.08	-1.53	0.011	*HERC6*	HECT and RLD domain containing E3 ubiquitin protein ligase family member 6
TC1200007627 .hg.1	4.29	3.44	1.8	0.003	*HIGD1C*	HIG1 hypoxia inducible domain family, member 1C

TC1700011087.hg.1	4.03	4.82	-1.73	0.031	*HILS1*	Histone linker H1 domain, spermatid-specific 1, pseudogene
TC0800012285.hg.1	4.02	4.72	-1.62	0.022	*HMBOX1*	Homeobox containing 1
TSUnmapped00000682.hg.1	3.77	4.43	-1.58	0.027	*HMBS*	Hydroxymethylbilane synthase
TC0X00008724.hg.1	4.92	5.53	-1.52	0.033	*HMGB3*	High mobility group box 3
TC0700007445.hg.1	5.41	6.23	-1.76	0.007	*HMGN1P19*	High mobility group nucleosome binding domain 1 pseudogene 19 [Source:HGNC Symbol;Acc:HGNC:39363]
TC2100006905.hg.1	5.18	5.89	-1.64	0.021	*HMGN1P2*	High mobility group nucleosome binding domain 1 pseudogene 2 [Source:HGNC Symbol;Acc:HGNC:4985]
TC1500009685.hg.1	4.62	5.36	-1.67	0.019	*HMGN1P26*	High mobility group nucleosome binding domain 1 pseudogene 26 [Source:HGNC Symbol;Acc:HGNC:39370]
TC0300009228.hg.1	7.72	8.53	-1.75	0.014	*HMGN2P13*	High mobility group nucleosomal binding domain 2 pseudogene 13 [Source:HGNC Symbol;Acc:HGNC:33557]
TC0900011118.hg.1	4.53	5.37	-1.79	0.003	*HMGN2P32*	High mobility group nucleosomal binding domain 2 pseudogene 32 [Source:HGNC Symbol;Acc:HGNC:39403]
TC1000008268.hg.1	5.9	6.72	-1.77	0.007	*HMGN2P8*	High mobility group nucleosomal binding domain 2 pseudogene 8 [Source:HGNC Symbol;Acc:HGNC:31662]
TC0600007357.hg.1	4.4	3.58	1.77	2.17E-05	*HNRNPA1P1*	Heterogeneous nuclear ribonucleoprotein A1 pseudogene 1 [Source:HGNC Symbol;Acc:HGNC:13957]
TSUnmapped00000646.hg.1	3.25	4.01	-1.7	0.005	*HNRNPCL3*	Heterogeneous nuclear ribonucleoprotein C-like 3 [Source:HGNC Symbol;Acc:HGNC:51235]
TC0X00007451.hg.1	7.01	7.6	-1.51	0.045	*HNRNPDP1*	Heterogeneous nuclear ribonucleoprotein D pseudogene 1 [Source:HGNC Symbol;Acc:HGNC:5038]
TC1000007852.hg.1	5.49	6.53	-2.06	0.012	*HNRNPH3*	Heterogeneous nuclear ribonucleoprotein H3 (2H9)
TC1200012637.hg.1	3.51	4.31	-1.75	0.018	*HOXC10*	Homeobox C10
TC0400012458.hg.1	4.66	3.31	2.54	0.004	*HPGD*	Hydroxyprostaglandin dehydrogenase 15-(NAD)
TC1800007829.hg.1	4.24	4.86	-1.54	0.043	*HSBP1L1*	Heat shock factor binding protein 1-like 1
TC0300008012.hg.1	4.48	3.88	1.51	0.019	*HTR1F*	5-hydroxytryptamine (serotonin) receptor 1F, G protein-coupled
TC1200007050.hg.1	4.33	3.71	1.54	0.013	*IAPP*	Islet amyloid polypeptide
TC0600012413.hg.1	4.92	4.16	1.69	0.009	*IBTK*	Inhibitor of Bruton agammaglobulinemia tyrosine kinase
TC0100018323.hg.1	4.61	3.9	1.64	0.008	*IER5*	Immediate early response 5
TC0200010745.hg.1	6.15	5.33	1.77	0.003	*IGFBP2*	Insulin like growth factor binding protein 2
TC1400010444.hg.1	10.49	7.82	6.35	0.038	*IGHA1*	Immunoglobulin heavy constant alpha 1
TC1400010546.hg.1	3.25	3.96	-1.63	0.015	*IGHV1-68*	Immunoglobulin heavy variable 1-68 (pseudogene)
TC1400010538.hg.1	3.07	3.85	-1.71	0.024	*IGHV3-63*	Immunoglobulin heavy variable 3-63 (pseudogene)
TC1400010526.hg.1	6.17	6.8	-1.55	0.003	*IGHVIII-51-1*	Immunoglobulin heavy variable (III)-51-1 (pseudogene)
TC2200006766	5.28	4.37	1.88	0.009	*IGLV10-54*	Immunoglobulin lambda variable 10-54

.hg.1						
TC0400011756 .hg.1	3.47	4.11	-1.56	0.007	*IL21*	Interleukin 21
TC0500012120 .hg.1	4.43	3.79	1.56	0.028	*IL9*	Interleukin 9
TC1100010451 .hg.1	4.41	3.78	1.55	0.035	*IMMP1L*	Inner mitochondrial membrane peptidase subunit 1
TC0100014501 .hg.1	3.36	4.04	-1.61	0.015	*INSL5*	Insulin-like 5
TC0200016166 .hg.1	3.81	4.47	-1.59	0.031	*IQCA1*	IQ motif containing with AAA domain 1
TC0200011980 .hg.1	5.51	3.64	3.65	0.001	*ITSN2*	Intersectin 2
TC0200016727 .hg.1	4.19	4.87	-1.6	0.003	*IWS1*	IWS1 homolog (S. cerevisiae)
TC0800010258 .hg.1	4.52	3.92	1.51	0.047	*KAT6A*	K(lysine) acetyltransferase 6A
TC0400010205 .hg.1	4.11	4.71	-1.52	0.028	*KCNIP4*	Kv channel interacting protein 4
TC0600011733 .hg.1	6.68	5.09	3.01	0.001	*KCNK5*	Potassium channel, two pore domain subfamily K, member 5
TC1800008338 .hg.1	6.18	5.53	1.57	0.023	*KCTD1*	Potassium channel tetramerization domain containing 1
TC0900009788 .hg.1	3.36	4.17	-1.76	0.028	*KCTD10P1*	Potassium channel tetramerization domain containing 10 pseudogene 1 [Source:HGNC Symbol;Acc:HGNC:49807]
TC0700013510 .hg.1	5.7	4.82	1.84	0.042	*KDELR2*	KDEL (Lys-Asp-Glu-Leu) endoplasmic reticulum protein retention receptor 2
TC1600007317 .hg.1	4.2	3.5	1.62	0.024	*KIAA0556*	KIAA0556
TC0200013600 .hg.1	6.12	6.72	-1.51	0.033	*KIAA1211L*	KIAA1211-like
TC0600008346 .hg.1	3.31	4.42	-2.16	0.016	*KIAA1586*	KIAA1586
TC0600010241 .hg.1	7.84	8.59	-1.68	0.020	*KIF25*	Kinesin family member 25
TC1000009742 .hg.1	4.42	3.79	1.55	0.012	*KIN*	Kin17 DNA and RNA binding protein
TC1700010641 .hg.1	2.97	3.82	-1.8	0.003	*KRT23*	Keratin 23, type I
TC2100007910 .hg.1	4.23	3.45	1.72	0.041	*KRTAP21-2*	Keratin associated protein 21-2
TC0200009537 .hg.1	4.69	4	1.61	0.023	*KYNU*	Kynureninase
TC1600006557 .hg.1	4.01	3.35	1.58	0.011	*LA16c-395F10.2; sharru*	Novel transcript, antisense to IFT140; Transcript Identified by AceView
TC0X00008467 .hg.1	3.51	4.32	-1.75	0.014	*LARP1BP3*	La ribonucleoprotein domain family member 1B pseudogene 3 [Source:HGNC Symbol;Acc:HGNC:44085]
TC1100010400 .hg.1	4.29	3.7	1.5	0.045	*LGR4*	Leucine-rich repeat containing G protein-coupled receptor 4
TC1900012043 .hg.1	4.83	4.11	1.65	0.019	*LILRA4*	Leukocyte immunoglobulin-like receptor, subfamily A (with TM domain), member 4
TC0X00006455 .hg.1	4.23	5.01	-1.71	0.001	*LINC00106*	Long intergenic non-protein coding RNA 106
TC0Y00006456 .hg.1	4.1	4.99	-1.85	3E-04	*LINC00106; myby*	Homo sapiens long intergenic non-protein Coding RNA 106 (LINC00106), long non-coding RNA.; long intergenic non-protein coding RNA 106 [Source:HGNC Symbol;Acc:HGNC:31843]; long intergenic non-protein coding RNA 106 [Source:HGNC

						Symbol;Acc:31843]; Transcript Identified by AceView
TC0100006682.hg.1	7.28	7.91	-1.55	0.022	*LINC00337*	Long intergenic non-protein coding RNA 337
TC0200013535.hg.1	4.28	5.11	-1.77	0.006	*LMAN2L*	Lectin, mannose-binding 2-like
TC1100008502.hg.1	4.37	5.22	-1.8	0.006	*LOC100506127; RP11-111M22.2; LOC387790*	Putative uncharacterized protein FLJ37770-like; Transcript Identified by AceView, Entrez Gene ID(s) 387790; novel transcript
TC1100006992.hg.1	3.4	4.21	-1.76	0.043	*LOC494141*	Solute carrier family 25, member 51 pseudogene; Transcript Identified by AceView, Entrez Gene ID(s) 494141, RefSeq ID(s) NR_026541; Transcript Identified by AceView, Entrez Gene ID(s) 494141, RefSeq ID(s) NR_026564
TC1300006630.hg.1	4.05	3.25	1.74	7.44E-05	*LSP1P1*	Lymphocyte-specific protein 1 pseudogene 1 [Source:HGNC Symbol;Acc:HGNC:39716]
TC0100009474.hg.1	4.85	4.1	1.67	0.018	*MAGI3*	Membrane associated guanylate kinase, WW and PDZ domain containing 3
TC0X00010130.hg.1	6.81	4.82	3.97	0.015	*MAGT1*	Magnesium transporter 1
TC0200007048.hg.1	3.95	3.22	1.66	0.001	*MAPRE3*	Microtubule-associated protein, RP/EB family, member 3
TC1800008842.hg.1	3.4	4.12	-1.65	0.004	*MC4R*	Melanocortin 4 receptor
TC1900011657.hg.1	5.04	5.71	-1.58	0.027	*MCEMP1*	Mast cell-expressed membrane protein 1
TC0600013004.hg.1	3.65	4.25	-1.51	0.026	*MCM9*	Minichromosome maintenance 9 homologous recombination repair factor
TC0500011483.hg.1	6.08	4.91	2.25	0.036	*MCTP1*	Multiple C2 domains, transmembrane 1
TC0300013068.hg.1	4.56	3.8	1.7	0.018	*MECOM*	MDS1 and EVI1 complex locus
TC0500011418.hg.1	8.12	9.85	-3.31	0.05	*MEF2C*	Myocyte enhancer factor 2C
TC0300013896.hg.1	5.25	4.38	1.83	0.048	*MFSD1*	Major facilitator superfamily domain containing 1
TC1000008351.hg.1	4.23	3.51	1.65	0.006	*MINPP1*	Multiple inositol-polyphosphate phosphatase 1
TC0500012757.hg.1	4.61	3.68	1.92	4E-04	*MIR103A1*	MicroRNA 103a-1
TC0700012578.hg.1	5.49	6.16	-1.59	0.020	*MIR182*	MicroRNA 182
TC0X00010859.hg.1	4.3	3.4	1.87	0.003	*MIR19B2*	MicroRNA 19b-2
TC0100016507.hg.1	4.09	3.4	1.61	0.040	*MIR488*	MicroRNA 488
TC1400008277.hg.1	3.09	3.77	-1.6	0.006	*MIR495*	MicroRNA 495
TC0400009324.hg.1	5.82	4.56	2.39	0.017	*MIR548T*	MicroRNA 548t
TC0300009475.hg.1	4.25	3.6	1.57	0.003	*MIR6828*	MicroRNA 6828
TC1600007980.hg.1	4.63	5.71	-2.1	0.026	*MIR6863*	MicroRNA 6863
TC1200011450.hg.1	6.18	6.8	-1.54	0.002	*MKRN9P*	Makorin ring finger protein 9, pseudogene
TC0X00006949.hg.1	4.09	4.7	-1.53	0.047	*MOB1AP2*	MOB kinase activator 1A pseudogene 2 [Source:HGNC Symbol;Acc:HGNC:42639]
TC0600013883.hg.1	5.95	6.66	-1.64	0.019	*MPC1*	Mitochondrial pyruvate carrier 1

TC1400007491 .hg.1	5.4	6.45	-2.06	3.20E-05	*MPP5*	Membrane protein, palmitoylated 5
TC0600011822 .hg.1	4.12	4.84	-1.65	0.011	*MRPS10*	Mitochondrial ribosomal protein S10
TC2200008939 .hg.1	3.81	4.99	-2.26	0.020	*MRPS18CP6*	Mitochondrial ribosomal protein S18C pseudogene 6 [Source:HGNC Symbol;Acc:HGNC:29747]
TC1100007763 .hg.1	5.83	5.08	1.68	0.038	*MS4A3*	Membrane-spanning 4-domains, subfamily A, member 3 (hematopoietic cell-specific)
TC1100007776 .hg.1	5.09	4.45	1.56	0.017	*MS4A8*	Membrane-spanning 4-domains, subfamily A, member 8
TC0700007879 .hg.1	4.45	3.68	1.72	0.014	*MTCO3P41*	MT-CO3 pseudogene 41 [Source:HGNC Symbol;Acc:HGNC:52144]
TC0800008314 .hg.1	15.61	16.48	-1.83	0.021	*MTDH*	Metadherin
TC1600011147 .hg.1	5.79	5.2	1.5	0.024	*MTHFSD*	Methenyltetrahydrofolate synthetase domain containing
TC0X00009882 .hg.1	3.8	4.43	-1.54	0.005	*MTMR8*	Myotubularin related protein 8
TC0900006541 .hg.1	4.31	3.57	1.67	0.027	*MTND1P11*	Mitochondrially encoded NADH:ubiquinone oxidoreductase core subunit 1 pseudogene 11 [Source:HGNC Symbol;Acc:HGNC:42060]
TC0400010098 .hg.1	5.93	6.6	-1.59	0.017	*MTND2P31*	Mitochondrially encoded NADH:ubiquinone oxidoreductase core subunit 2 pseudogene 31 [Source:HGNC Symbol;Acc:HGNC:42132]
TC0500012092 .hg.1	5.29	4.64	1.57	0.046	*MTND3P25*	Mitochondrially encoded NADH:ubiquinone oxidoreductase core subunit 3 pseudogene 25 [Source:HGNC Symbol;Acc:HGNC:52172]
TC0X00008361 .hg.1	6.99	5.21	3.44	0.002	*MTND4LP1*	Mitochondrially encoded NADH:ubiquinone oxidoreductase core subunit 4L pseudogene 1 [Source:HGNC Symbol;Acc:HGNC:31347]
TC0X00008360 .hg.1	6.68	5.72	1.95	0.040	*MTND4P24; MTCO1P53*	Mitochondrially encoded NADH:ubiquinone oxidoreductase core subunit 4 pseudogene 24 [Source:HGNC Symbol;Acc:HGNC:42220]; MT-CO1 pseudogene 53 [Source:HGNC Symbol;Acc:HGNC:52118]
TC0300011941 .hg.1	3.73	4.44	-1.64	0.004	*MYH15*	Myosin, heavy chain 15
TC0600007060 .hg.1	4.89	4.19	1.63	0.028	*MYLIP; MIR4639*	Myosin regulatory light chain interacting protein; microRNA 4639
TC1300007125 .hg.1	3.67	4.46	-1.73	0.016	*NAP1L4P3*	Nucleosome assembly protein 1-like 4 pseudogene 3 [Source:HGNC Symbol;Acc:HGNC:39738]
TC0100015162 .hg.1	4.55	5.37	-1.76	0.044	*NBPF4*	Neuroblastoma breakpoint family, member 4
TC0100016656 .hg.1	4.15	3.48	1.58	0.002	*NCF2*	Neutrophil cytosolic factor 2
TC0200016757 .hg.1	6.93	4.84	4.26	0.026	*NCKAP1*	NCK-associated protein 1
TC1600007520 .hg.1	4.33	3.59	1.68	0.004	*NDUFA3P6*	NADH:ubiquinone oxidoreductase subunit A3 pseudogene 6 [Source:HGNC Symbol;Acc:HGNC:45055]
TC1300007586 .hg.1	3.21	3.88	-1.59	0.001	*NIPA2P5*	Non imprinted in Prader-Willi/Angelman syndrome 2 pseudogene 5 [Source:HGNC Symbol;Acc:HGNC:42045]
TC0900010445 .hg.1	5.75	5.09	1.58	0.009	*NMRK1*	Nicotinamide riboside kinase 1

TC0600011272.hg.1	4.64	5.24	-1.51	0.044	*NOP56P1*	NOP56 ribonucleoprotein pseudogene 1 [Source:HGNC Symbol;Acc:HGNC:13962]
TC1400006859.hg.1	3.91	3.05	1.81	0.030	*NPAS3*	Neuronal PAS domain protein 3
TC1500009671.hg.1	4.64	5.26	-1.54	0.011	*NPM1P47*	Nucleophosmin 1 (nucleolar phosphoprotein B23, numatrin) pseudogene 47 [Source:HGNC Symbol;Acc:HGNC:45226]
TC0700010512.hg.1	4.14	3.38	1.7	0.031	*NPVF*	Neuropeptide VF precursor
TC1500008442.hg.1	4.77	3.71	2.08	0.011	*NR2F2*	Nuclear receptor subfamily 2, group F, member 2
TC0X00010401.hg.1	3.68	4.34	-1.58	0.013	*NXF3*	Nuclear RNA export factor 3
TC0700006632.hg.1	5.53	6.21	-1.6	0.007	*OCM*	Oncomodulin
TC0900008869.hg.1	5.02	5.92	-1.86	0.009	*ODF2*	Outer dense fiber of sperm tails 2
TC1100007760.hg.1	3.94	4.59	-1.57	0.034	*OOSP1*	Oocyte secreted protein 1, pseudogene
TC0300013566.hg.1	4.49	3.39	2.15	0.004	*OPA1-AS1*	OPA1 antisense RNA 1
TC1100012172.hg.1	4.46	3.71	1.68	0.003	*OR2AL1P*	Olfactory receptor, family 2, subfamily AL, member 1 pseudogene
TC0100018121.hg.1	4.61	3.76	1.8	0.005	*OR2T12*	Olfactory receptor, family 2, subfamily T, member 12
TC1100010824.hg.1	4.35	3.31	2.06	2.01E-05	*OR4C11*	Olfactory receptor, family 4, subfamily C, member 11
TC1100010939.hg.1	3.68	4.42	-1.66	0.019	*OR5B1P*	Olfactory receptor, family 5, subfamily B, member 1 pseudogene
TC1100010864.hg.1	3.63	4.32	-1.61	0.019	*OR5M10*	Olfactory receptor, family 5, subfamily M, member 10
TC1100012640.hg.1	3.92	4.93	-2.01	0.001	*OR6X1*	Olfactory receptor, family 6, subfamily X, member 1
TC1100006955.hg.1	5.17	4.24	1.91	0.008	*OR7E14P*	Olfactory receptor, family 7, subfamily E, member 14 pseudogene
TC1100012667.hg.1	3.13	3.94	-1.76	0.038	*OR8B3*	Olfactory receptor, family 8, subfamily B, member 3
TC0100007184.hg.1	5.46	6.27	-1.75	0.002	*OTUD3*	OTU deubiquitinase 3
TC1100013141.hg.1	3.95	4.54	-1.51	0.046	*OVCH2*	Ovochymase 2 (gene/pseudogene)
TC0300008559.hg.1	3.23	3.84	-1.53	0.009	*PARP15*	Poly(ADP-ribose) polymerase family member 15
TC0Y00007283.hg.1	5	4.09	1.87	0.013	*PARP4P1*	Poly(ADP-ribose) polymerase family member 4 pseudogene 1 [Source:HGNC Symbol;Acc:HGNC:18500]
TC0X00007834.hg.1	3.82	3.2	1.53	0.001	*PCDH11X*	Protocadherin 11 X-linked
TC0500008875.hg.1	4.19	5.26	-2.1	0.037	*PCDHB2*	Protocadherin beta 2
TC0400011404.hg.1	5.08	4.02	2.08	0.029	*PCNAP1*	Proliferating cell nuclear antigen pseudogene 1
TC1000008904.hg.1	4.47	3.68	1.74	0.003	*PDCD4; MIR4680*	Programmed cell death 4 (neoplastic transformation inhibitor); microRNA 4680
TC1600010740.hg.1	8.47	7.06	2.65	0.036	*PDXDC2P*	Pyridoxal-dependent decarboxylase domain containing 2, pseudogene
TC2100007321.hg.1	11.65	9.58	4.18	0.038	*PDXK*	Pyridoxal (pyridoxine, vitamin B6) kinase
TC1400007259.hg.1	4.09	3.35	1.68	0.022	*PELI2*	Pellino E3 ubiquitin protein ligase family member 2

TC1100007795.hg.1	7.96	4.47	11.28	0.002	*PGA3; PGA4*	Pepsinogen 3, group I (pepsinogen A); pepsinogen 4, group I (pepsinogen A)
TC1100013016.hg.1	7.33	4.37	7.77	0.004	*PGA4*	Pepsinogen 4, group I (pepsinogen A)
TC1100013017.hg.1	9.25	4.56	25.79	0.009	*PGA5*	Pepsinogen 5, group I (pepsinogen A)
TC0100012379.hg.1	3.12	3.96	-1.8	0.014	*PGBD2*	PiggyBac transposable element derived 2
TC0X00009124.hg.1	4.85	5.63	-1.72	0.006	*PIGA*	Phosphatidylinositol glycan anchor biosynthesis class A
TC1500007303.hg.1	6.34	5.36	1.97	0.027	*PIGB*	Phosphatidylinositol glycan anchor biosynthesis class B
TC0X00009317.hg.1	3.23	3.96	-1.65	0.036	*PIGFP3*	Phosphatidylinositol glycan anchor biosynthesis class F pseudogene 3 [Source:HGNC Symbol;Acc:HGNC:45151]
TC0500011672.hg.1	7.62	6.64	1.98	0.015	*PJA2*	Praja ring finger 2, E3 ubiquitin protein ligase
TC0200007399.hg.1	7.53	5.83	3.24	0.027	*PLEKHH2*	Pleckstrin homology domain containing, family H (with MyTH4 domain) member 2
TC0300014061.hg.1	3.87	4.54	-1.59	0.009	*PLSCR2*	Phospholipid scramblase 2
TC0500007722.hg.1	4.28	5.23	-1.93	0.034	*PMCHL2*	Pro-melanin-concentrating hormone-like 2, pseudogene
TC0200010791.hg.1	4.28	3.64	1.57	0.016	*PNKD; MIR6810*	Paroxysmal nonkinesigenic dyskinesia; microRNA 6810
TC1400009108.hg.1	4.41	3.62	1.72	0.046	*POLE2*	Polymerase (DNA directed), epsilon 2, accessory subunit
TC1400008557.hg.1	4.16	5.01	-1.8	0.020	*POTEG; POTEM*	POTE ankyrin domain family, member G; POTE ankyrin domain family, member M
TC0100018219.hg.1	5.56	4.6	1.94	0.029	*PPCS; CCDC30*	Phosphopantothenoylcysteine synthetase; coiled-coil domain containing 30
TC0X00006725.hg.1	3.99	3.28	1.63	0.006	*PPEF1*	Protein phosphatase, EF-hand calcium binding domain 1
TC1400007354.hg.1	11.2	6.92	19.43	0.025	*PPM1A*	Protein phosphatase, Mg2+/Mn2+ dependent, 1A
TC2200007940.hg.1	3.27	3.95	-1.6	0.016	*PPP1R26P2*	Protein phosphatase 1 regulatory subunit 26 pseudogene 2 [Source:HGNC Symbol;Acc:HGNC:42016]
TSUnmapped00000660.hg.1	4.16	3.52	1.55	0.022	*PRAMEF18*	PRAME family member 18
TSUnmapped00000570.hg.1	4.81	4.19	1.55	0.027	*PRAMEF27*	PRAME family member 27 [Source:HGNC Symbol;Acc:HGNC:51234]
TC1200012760.hg.1	4.98	5.77	-1.73	0.026	*PRR4*	Proline rich 4 (lacrimal)
TC0Y00006767.hg.1	3.71	4.35	-1.57	0.028	*PRY2; PRY*	PTPN13-like, Y-linked 2; PTPN13-like, Y-linked
TC0600014110.hg.1	3.57	4.24	-1.59	0.022	*PSMB9*	Proteasome subunit beta 9
TC0700007018.hg.1	4.58	3.94	1.56	0.032	*PSMC1P2*	Proteasome (prosome, macropain) 26S subunit, ATPase, 1 pseudogene 2 [Source:HGNC Symbol;Acc:HGNC:39777]
TC0700007623.hg.1	7.6	8.33	-1.66	0.019	*PSPHP1*	Phosphoserine phosphatase pseudogene 1 [Source:HGNC Symbol;Acc:HGNC:9578]
TC1400009422.hg.1	4.41	5.06	-1.57	0.014	*RAB15*	RAB15, member RAS oncogene family
TC1000007103.hg.1	5.9	4.49	2.66	0.013	*RAB18*	RAB18, member RAS oncogene family
TC1200012663.hg.1	3.72	4.47	-1.68	0.022	*RAB3IP*	RAB3A interacting protein
TC0300012482.hg.1	5.32	4.7	1.55	0.014	*RAB6B*	RAB6B, member RAS oncogene family

TC1200008107 .hg.1	5.12	4.11	2.01	0.046	*RAP1B*	RAP1B, member of RAS oncogene family
TC0500008014 .hg.1	5.09	4.41	1.61	0.031	*RASA1*	RAS p21 protein activator (GTPase activating protein) 1
TC0900010565 .hg.1	4.45	3.72	1.65	0.026	*RASEF*	RAS and EF-hand domain containing
TC1200011435 .hg.1	4.42	3.7	1.65	0.037	*RASSF9*	Ras association (RalGDS/AF-6) domain family (N-terminal) member 9
TC2000009026 .hg.1	5.03	3.94	2.13	0.049	*RBL1*	Retinoblastoma-like 1
TC1100013096 .hg.1	3.63	4.49	-1.81	0.012	*RBM7*	RNA binding motif protein 7
TC0Y00007216 .hg.1	3.58	4.17	-1.51	0.015	*RBMY2WP*	RNA binding motif protein, Y-linked, family 2, member W pseudogene [Source:HGNC Symbol;Acc:HGNC:23900]
TC0800007957 .hg.1	4.26	3.16	2.14	0.040	*RDH10*	Retinol dehydrogenase 10 (all-trans)
TC0600009171 .hg.1	4.22	5.07	-1.79	0.023	*RFPL4B*	Ret finger protein-like 4B
TC0X00007122 .hg.1	5.12	5.89	-1.7	0.047	*RGN*	Regucalcin
TC1200006730 .hg.1	6.55	5.62	1.9	6.03E-06	*RIMKLB*	Ribosomal modification protein rimK-like family member B
TC0600011001 .hg.1	5.67	4.81	1.82	0.044	*RN7SL128P; AL136303.2*	RNA, 7SL, cytoplasmic 128, pseudogene [Source:HGNC Symbol;Acc:HGNC:46144]
TC1400006540 .hg.1	4	3.38	1.54	0.025	*RNASE2*	Ribonuclease, RNase A family, 2 (liver, eosinophil-derived neurotoxin)
TC0X00006663 .hg.1	4.16	3.55	1.53	0.025	*RP1-122K4.2; sportoy*	Novel transcript; Transcript Identified by AceView
TC0100010809 .hg.1	3.96	3.27	1.62	0.036	*RP11-46A10.2; vorbu*	Putative novel transcript; Transcript Identified by AceView
TC1400008920 .hg.1	3.8	4.47	-1.59	0.025	*RPL12P6*	Ribosomal protein L12 pseudogene 6 [Source:HGNC Symbol;Acc:HGNC:23538]
TC2100007851 .hg.1	4.53	5.19	-1.58	0.034	*RPL23P2*	Ribosomal protein L23 pseudogene 2
TC1200011103 .hg.1	3.79	4.69	-1.86	0.029	*RPL7P39*	Ribosomal protein L7 pseudogene 39 [Source:HGNC Symbol;Acc:HGNC:36214]
TC0X00007850 .hg.1	3.44	4.34	-1.86	0.007	*RPL7P55*	Ribosomal protein L7 pseudogene 55 [Source:HGNC Symbol;Acc:HGNC:36105]
TC1700011000 .hg.1	4.53	5.15	-1.54	0.008	*RPL9P28*	Ribosomal protein L9 pseudogene 28
TC1100013147 .hg.1	4.94	3.93	2.02	0.007	*RPS13; SNORD14B*	Ribosomal protein S13; small nucleolar RNA, C/D box 14B
TC0900011136 .hg.1	5.25	5.97	-1.65	0.004	*RPS15AP27*	Ribosomal protein S15a pseudogene 27 [Source:HGNC Symbol;Acc:HGNC:37011]
TC2000007273 .hg.1	5.09	4.39	1.62	0.048	*RPS27AP3*	Ribosomal protein S27a pseudogene 3
TC0600008593 .hg.1	4.06	5.01	-1.94	0.001	*RPS6P7*	Ribosomal protein S6 pseudogene 7
TC0300011525 .hg.1	4.85	4.08	1.71	0.046	*RYBP*	RING1 and YY1 binding protein
TC0200014819 .hg.1	4.44	5.29	-1.8	0.018	*SCN9A*	Sodium channel, voltage gated, type IX alpha subunit
TC0900010070 .hg.1	6.18	7.1	-1.89	0.041	*SDR42E1P1*	Short chain dehydrogenase/reductase family 42E, member 1 pseudogene 1 [Source:HGNC Symbol;Acc:HGNC:50378]
TC0900010151 .hg.1	8.1	9.33	-2.36	0.017	*SDR42E1P2*	Short chain dehydrogenase/reductase family 42E, member 1 pseudogene 2 [Source:HGNC Symbol;Acc:HGNC:51833]
TC0100018473 .hg.1	3.7	3.11	1.51	0.008	*SEC22B*	Salzman2013 ALT_ACCEPTOR, CDS, coding, INTERNAL, OVCODE, OVEXON best

						transcript NM_004892; Salzman2013 ANNOTATED, CDS, coding, OVCODE, OVEXON, UTR3 best transcript NM_004892
TC2000006833 .hg.1	4.58	3.34	2.35	0.006	*SEC23B*	Sec23 homolog B, COPII coat complex component
TC1400009842 .hg.1	6.07	5.26	1.76	0.022	*SEL1L*	Sel-1 suppressor of lin-12-like (C. elegans)
TC1500010783 .hg.1	4.27	3.46	1.76	0.013	*SEMA4B*	Sema domain, immunoglobulin domain (Ig), transmembrane domain (TM) and short cytoplasmic domain, (semaphorin) 4B
TC1500007777 .hg.1	4.17	3.26	1.87	0.001	*SENP8*	SUMO/sentrin peptidase family member, NEDD8 specific
TC0400011729 .hg.1	4.44	5.18	-1.67	0.004	*SETP12*	SET pseudogene 12 [Source:HGNC Symbol;Acc:HGNC:42931]
TC0600013231 .hg.1	8.52	5.44	8.47	0.047	*SGK1*	Serum/glucocorticoid regulated kinase 1
TC0200010897 .hg.1	5.26	4.27	2	0.034	*SGPP2*	Sphingosine-1-phosphate phosphatase 2
TC0500007943 .hg.1	4.25	4.92	-1.59	0.044	*SHFM1P1*	Split hand/foot malformation (ectrodactyly) type 1 pseudogene 1 [Source:HGNC Symbol;Acc:HGNC:24454]
TC0400007901 .hg.1	5.99	4.48	2.84	0.004	*SHROOM3*	Shroom family member 3
TC1200010046 .hg.1	4	3.38	1.53	0.004	*SLC15A5*	Solute carrier family 15, member 5
TC0900008482 .hg.1	4.48	3.66	1.77	0.002	*SLC31A2*	Solute carrier family 31 (copper transporter), member 2
TC0300012881 .hg.1	5.73	4.77	1.94	0.012	*SLC33A1*	Solute carrier family 33 (acetyl-CoA transporter), member 1
TC0600014154 .hg.1	4.32	3.64	1.6	0.049	*SLC35A1*	Solute carrier family 35 (CMP-sialic acid transporter), member A1
TC0200013943 .hg.1	5.49	3.65	3.58	2E-04	*SLC35F5*	Solute carrier family 35, member F5
TC1800008474 .hg.1	5.43	3.88	2.93	0.002	*SLC39A6*	Solute carrier family 39 (zinc transporter), member 6
TC1700008867 .hg.1	3.88	3.29	1.5	0.042	*SLC9A3R1; MIR3615*	Solute carrier family 9, subfamily A (NHE3, cation proton antiporter 3), member 3 regulator 1; microRNA 3615
TC0400008909 .hg.1	6.12	5.18	1.92	0.028	*SMAD1*	SMAD family member 1
TC1800009283 .hg.1	4.53	3.53	2.01	0.002	*SMAD2*	SMAD family member 2
TC0100015991 .hg.1	5.82	4.42	2.64	0.030	*SMG5*	SMG5 nonsense mediated mRNA decay factor
TC1700008915 .hg.1	4.72	4.01	1.63	0.002	*SMIM6*	Small integral membrane protein 6
TC2000007318 .hg.1	7.12	5.25	3.66	0.014	*SNORA71E; SNORA60; SNHG11*	Small nucleolar RNA, H/ACA box 71E; small nucleolar RNA, H/ACA box 60; small nucleolar RNA host gene 11
TC0100013552 .hg.1	5.15	4.27	1.84	0.004	*SNORD103C*	Small nucleolar RNA, C/D box 103C
TC1500006601 .hg.1	4.21	4.91	-1.62	0.047	*SNORD115-27*	Small nucleolar RNA, C/D box 115-27
TC1500006609 .hg.1	8.78	9.9	-2.17	0.014	*SNORD115-45*	Small nucleolar RNA, C/D box 115-45
TC0200010482 .hg.1	7.34	8.68	-2.54	0.017	*SNORD11B*	Small nucleolar RNA, C/D box 11B
TC0100007504 .hg.1	4.38	5.06	-1.61	0.034	*SNRPEP7*	Small nuclear ribonucleoprotein polypeptide E pseudogene 7 [Source:HGNC Symbol;Acc:HGNC:43572]
TC1700008454 .hg.1	4.31	5.03	-1.65	0.049	*SNRPGP17*	Small nuclear ribonucleoprotein polypeptide G pseudogene 17

						[Source:HGNC Symbol;Acc:HGNC:49372]
TC0600012067 .hg.1	3.34	3.97	-1.55	0.032	*SOD1P1*	Superoxide dismutase 1, soluble pseudogene 1 [Source:HGNC Symbol;Acc:HGNC:45134]
TC1100008447 .hg.1	4.58	3.63	1.93	0.021	*SPCS2*	Signal peptidase complex subunit 2
TC0700008568 .hg.1	6.15	5.16	1.98	0.025	*SRRT*	Serrate, RNA effector molecule
TC0700009130 .hg.1	3.52	4.28	-1.7	0.002	*SSMEM1*	Serine-rich single-pass membrane protein 1
TC0X00007176 .hg.1	4.24	4.87	-1.54	0.002	*SSX1*	Synovial sarcoma, X breakpoint 1
TC1700011813 .hg.1	5.61	4.54	2.09	0.023	*ST6GALNAC1*	ST6 (alpha-N-acetyl-neuraminyl-2,3-beta-galactosyl-1,3)-N-acetylgalactosaminide alpha-2,6-sialyltransferase 1
TC1700010717 .hg.1	3.61	4.44	-1.78	0.008	*STAT5B*	Signal transducer and activator of transcription 5B
TC0100013323 .hg.1	4.12	3.18	1.91	0.007	*STPG1*	Sperm-tail PG-rich repeat containing 1
TC0600012785 .hg.1	4.66	3.73	1.9	0.029	*SUMO2P8*	SUMO2 pseudogene 8 [Source:HGNC Symbol;Acc:HGNC:39018]
TC1400008628 .hg.1	6.27	7.1	-1.78	0.030	*SUPT16H*	SPT16 homolog, facilitates chromatin remodeling subunit
TC0900007066 .hg.1	4.76	5.4	-1.55	0.034	*SYF2P2*	SYF2 pre-mRNA-splicing factor pseudogene 2 [Source:HGNC Symbol;Acc:HGNC:38139]
TC1700009040 .hg.1	5.81	4.72	2.14	0.004	*SYNGR2*	Synaptogyrin 2
TC0Y00006674 .hg.1	3.47	4.11	-1.56	0.027	*TAF9P1*	TATA-box binding protein associated factor 9 pseudogene 1 [Source:HGNC Symbol;Acc:HGNC:31743]
TC1300007340 .hg.1	3.6	4.31	-1.63	0.008	*TARDBPP2*	TAR DNA binding protein pseudogene 2 [Source:HGNC Symbol;Acc:HGNC:39848]
TC0400011978 .hg.1	5.11	4.41	1.63	0.029	*TBC1D9*	TBC1 domain family, member 9 (with GRAM domain)
TC1100009395 .hg.1	4.38	3.75	1.55	0.044	*TBRG1*	Transforming growth factor beta regulator 1
TC1700011354 .hg.1	3.72	4.39	-1.59	2E-04	*TBX2-AS1*	TBX2 antisense RNA 1
TC0X00010380 .hg.1	6.8	7.47	-1.59	0.031	*TCEAL6*	Transcription elongation factor A (SII)-like 6
TC0X00010406 .hg.1	4.14	4.8	-1.58	0.011	*TCEAL8*	Transcription elongation factor A (SII)-like 8
TC1100012561 .hg.1	2.95	3.58	-1.56	0.018	*TCEB1P22*	Transcription elongation factor B (SIII), polypeptide 1 pseudogene 22
TC0600007565 .hg.1	4.52	3.82	1.62	0.001	*TCF19*	Transcription factor 19
TC0500007684 .hg.1	3.74	4.41	-1.59	0.045	*TCONS_l2_00023407*	Salzman2013 ANNOTATED, INTERNAL, ncRNA, OVERLAPTX, OVEXON best transcript TCONS_l2_00023407
TC0400010618 .hg.1	3.56	4.17	-1.53	0.038	*TEC*	Tec protein tyrosine kinase
TC0400008318 .hg.1	4.64	3.96	1.59	0.020	*TET2*	Tet methylcytosine dioxygenase 2
TC0300006925 .hg.1	5.79	4.83	1.95	0.033	*TGFBR2*	Transforming growth factor beta receptor II
TC1400008748 .hg.1	3.76	4.37	-1.52	0.030	*TGM1*	Transglutaminase 1
TC0400007876 .hg.1	4.83	3.43	2.65	0.006	*THAP6*	THAP domain containing 6
TC0300006940 .hg.1	4.64	5.34	-1.63	0.031	*THRAP3P1*	Thyroid hormone receptor associated protein 3 pseudogene 1 [Source:HGNC Symbol;Acc:HGNC:32386]

TC0700010281 .hg.1	5.58	6.29	-1.63	0.015	*THSD7A*	Thrombospondin type 1 domain containing 7A
TC1300008829 .hg.1	3.12	3.76	-1.56	0.010	*TIMM9P3*	TIMM9 pseudogene 3 [Source:HGNC Symbol;Acc:HGNC:39930]
TC0400012309 .hg.1	3.2	3.93	-1.66	0.014	*TKTL2*	Transketolase-like 2
TC0300009136 .hg.1	4.03	3.29	1.67	0.019	*TM4SF1-AS1*	TM4SF1 antisense RNA 1
TC0100014947 .hg.1	4.42	3.71	1.64	0.024	*TMED5*	Transmembrane p24 trafficking protein 5
TC0400011746 .hg.1	4.44	5.04	-1.52	0.025	*TMEM155*	Transmembrane protein 155
TC0900011035 .hg.1	6.21	4.61	3.04	0.037	*TMEM246*	Transmembrane protein 246
TC0600012316 .hg.1	4.89	3.62	2.41	0.012	*TMEM30A*	Transmembrane protein 30A
TC1500009185 .hg.1	5.76	4.82	1.92	0.042	*TMEM87A*	Transmembrane protein 87A
TC1100010051 .hg.1	4.6	3.66	1.91	0.002	*TMEM9B*	TMEM9 domain family, member B
TC0600011960 .hg.1	5.06	4.26	1.74	0.004	*TNFRSF21*	Tumor necrosis factor receptor superfamily, member 21
TC0100018549 .hg.1	8.67	9.28	-1.52	0.017	*TNNT2*	Troponin T type 2 (cardiac)
TC0100016391 .hg.1	4.35	4.96	-1.53	0.042	*TOP1P1*	Topoisomerase (DNA) I pseudogene 1
TC0100010791 .hg.1	5.11	4.18	1.9	0.013	*TOR1AIP1*	Torsin A interacting protein 1
TC1400006640 .hg.1	7.51	8.22	-1.63	0.037	*TRAJ16*	T cell receptor alpha joining 16
TC1400006648 .hg.1	5.54	6.74	-2.3	0.020	*TRAJ2*	T cell receptor alpha joining 2 (non-functional)
TC1400006633 .hg.1	7.68	8.39	-1.64	0.017	*TRAJ33*	T cell receptor alpha joining 33
TC1400006622 .hg.1	12.98	13.77	-1.72	0.012	*TRAJ59*	T cell receptor alpha joining 59 (non-functional)
TC1400006604 .hg.1	3.73	4.32	-1.51	0.020	*TRAV32*	T cell receptor alpha variable 32 (pseudogene)
TC0700009470 .hg.1	7.49	6.53	1.95	0.030	*TRBJ1-4*	T cell receptor beta joining 1-4
TC0300014075 .hg.1	4.47	3.48	1.99	0.001	*TRIM59*	Tripartite motif containing 59
TC0900010958 .hg.1	4.21	3.39	1.77	0.025	*TRMO*	tRNA methyltransferase O
TC0200014577 .hg.1	4.35	3.74	1.53	0.005	*TXNP5*	Thioredoxin pseudogene 5 [Source:HGNC Symbol;Acc:HGNC:49485]
TC0100009859 .hg.1	6.46	7.37	-1.87	0.032	*U1*	U1 spliceosomal RNA [Source:RFAM;Acc:RF00003]
TC1700011264 .hg.1	3.59	4.38	-1.72	0.010	*U3*	Small nucleolar RNA U3 [Source:RFAM;Acc:RF00012]
TC0200014570 .hg.1	3.62	4.25	-1.55	0.013	*UBBP3*	Ubiquitin B pseudogene 3 [Source:HGNC Symbol;Acc:HGNC:12466]
TC0200010152 .hg.1	6.11	5.22	1.85	0.013	*UBE2E3*	Ubiquitin-conjugating enzyme E2E 3
TC1500010281 .hg.1	4.53	3.42	2.16	0.011	*UBE2Q2P12*	Ubiquitin conjugating enzyme E2Q family member 2 pseudogene 12 [Source:HGNC Symbol;Acc:HGNC:49523]
TC1500008140 .hg.1	5.24	4.55	1.61	0.045	*UBE2Q2P8*	Ubiquitin conjugating enzyme E2Q family member 2 pseudogene 8 [Source:HGNC Symbol;Acc:HGNC:49521]
TC1300008497 .hg.1	4.74	3.94	1.73	0.006	*UBL3*	Ubiquitin-like 3

TC0400012804 .hg.1	3.69	4.44	-1.68	0.022	*UGT2B25P*	UDP glucuronosyltransferase 2 family, polypeptide B25 pseudogene [Source:HGNC Symbol;Acc:HGNC:12549]
TC1200011671 .hg.1	4.23	5.04	-1.75	0.022	*UHRF1BP1L*	UHRF1 binding protein 1-like
TC1600009037 .hg.1	5.11	5.78	-1.59	0.016	*UNKL*	Unkempt family zinc finger-like
TC0100011913 .hg.1	3.94	4.82	-1.84	0.001	*URB2*	URB2 ribosome biogenesis 2 homolog (S. cerevisiae)
TC0X00007821 .hg.1	3.24	3.93	-1.62	0.013	*USP12PX*	Ubiquitin specific peptidase 12 pseudogene, X-linked [Source:HGNC Symbol;Acc:HGNC:21410]
TC0800009637 .hg.1	3.71	4.31	-1.51	0.008	*USP17L2*	Ubiquitin specific peptidase 17-like family member 2
TC1900008982 .hg.1	4.04	4.87	-1.77	0.027	*USP29*	Ubiquitin specific peptidase 29
TC1700010328 .hg.1	5.19	4.5	1.62	0.043	*UTP6*	UTP6, small subunit (SSU) processome component, homolog (yeast)
TC0300011706 .hg.1	3.75	4.45	-1.62	0.021	*VGLL3*	Vestigial-like family member 3
TC1500009669 .hg.1	7.24	6.6	1.55	0.036	*VPS13C*	Vacuolar protein sorting 13 homolog C (S. cerevisiae)
TC0300008374 .hg.1	4.31	4.94	-1.55	0.013	*VPS26AP1*	VPS26A pseudogene 1 [Source:HGNC Symbol;Acc:HGNC:44611]
TC0800006436 .hg.1	5.27	4.47	1.74	0.010	*WBP1LP3*	WW domain binding protein 1-like pseudogene 3 [Source:HGNC Symbol;Acc:HGNC:43958]
TC0100014534 .hg.1	5.88	4.66	2.34	0.021	*WLS*	Jeck2013 ALT_ACCEPTOR, ALT_DONOR, coding, INTERNAL, intronic, OVERLAPTX best transcript NM_024911
TC0X00008330 .hg.1	5.83	5.17	1.57	0.046	*XIAP*	X-linked inhibitor of apoptosis, E3 ubiquitin protein ligase
TC1200008002 .hg.1	5.75	6.37	-1.54	0.006	*XPOT*	Exportin, tRNA
TC0X00007501 .hg.1	4.97	4.31	1.58	0.016	*YIPF6*	Yip1 domain family member 6
TC0700012761 .hg.1	4.28	4.89	-1.53	0.007	*ZC3HAV1*	Zinc finger CCCH-type, antiviral 1
TSUnmapped0 0000274.hg.1	5.18	6	-1.77	0.004	*ZNF197*	Zinc finger protein 197
TC1900012028 .hg.1	7.94	8.56	-1.54	0.011	*ZNF320*	Zinc finger protein 320
TC1900007049 .hg.1	8.4	6.18	4.66	0.048	*ZNF440*	Zinc finger protein 440
TC1900011690 .hg.1	5.51	4.22	2.45	0.004	*ZNF493*	Zinc finger protein 493
TC0300007221 .hg.1	4.43	5.24	-1.76	0.006	*ZNF501*	Zinc finger protein 501
TC1800009086 .hg.1	3.89	4.73	-1.79	0.007	*ZNF516*	Zinc finger protein 516
TC1000008230 .hg.1	3.63	4.38	-1.68	0.003	*ZNF519P1*	Zinc finger protein 519 pseudogene 1 [Source:HGNC Symbol;Acc:HGNC:50785]
TC1900009729 .hg.1	4.42	5.32	-1.87	0.005	*ZNF563*	Zinc finger protein 563
TC1500007554 .hg.1	4.63	4.01	1.54	0.021	*ZNF609*	Zinc finger protein 609
TC1900011877 .hg.1	5.44	4.74	1.63	0.048	*ZNF625*	Zinc finger protein 625
TC1900011334 .hg.1	4.08	4.94	-1.83	0.038	*ZNF677*	Zinc finger protein 677
TC0400009681 .hg.1	5.72	3.96	3.39	0.014	*ZNF721; ABCA11P*	Zinc finger protein 721; ATP binding cassette subfamily A member 11,

						pseudogene
TC0900010925.hg.1	5.68	4.5	2.26	4E-04	*ZNF782*	Zinc finger protein 782
TC0200010193.hg.1	3.98	3.3	1.61	0.013	*ZNF804A*	Zinc finger protein 804A
TC1000010686.hg.1	4.26	5.11	-1.8	0.047	*ZWINT*	ZW10 interacting kinetochore protein
