# Supplementary material for: Identification of Thrombosis-Related Genes in Patients with Advanced Gastric Cancer: Data from AGAMENON-SEOM Registry
Source: Biomedicines. 2022 Jan 11;10(1):148. doi: 10.3390/biomedicines10010148 (PMC8773420; doi:10.3390/biomedicines10010148)
Supplement: Supplementary file 1 [file biomedicines-10-00148-s001.zip › biomedicines-1533227-supplementary/Table S3.pdf]

Table S3.

ID	T Avg (log2)	N Avg (log2)	Fold Change	P-value	Gene Symbol	Description
TC0200006440.hg.1	4.67	6.37	-3.24	0.004	*ACP1*	Acid phosphatase 1, soluble
TC0300009609.hg.1	7.15	8.02	-1.82	0.015	*ACTL6A*	Actin-like 6A
TC0500012032.hg.1	6.19	6.91	-1.65	0.041	*AFF4*	AF4/FMR2 family, member 4
TC0200010975.hg.1	4.22	5.45	-2.35	0.021	*AGFG1; MIR5703*	ArfGAP with FG repeats 1; microRNA 5703
TC0300012886.hg.1	4.32	5.03	-1.64	0.033	*ALG1L15P*	Asparagine-linked glycosylation 1-like 15, pseudogene [Source:HGNC Symbol;Acc:HGNC:44384]
TC1200012202.hg.1	4.22	5.05	-1.79	0.018	*ANAPC5*	Anaphase promoting complex subunit 5
TC2100008543.hg.1	3.92	5.26	-2.53	0.015	*ANKRD20A11P*	Ankyrin repeat domain 20 family, member A11, pseudogene
TC1300010015.hg.1	3.87	4.63	-1.69	0.012	*ANKRD20A9P; RNU6-55P*	Ankyrin repeat domain 20 family, member A9, pseudogene; RNA, U6 small nuclear 55, pseudogene
TC0700007198.hg.1	5.6	7	-2.63	0.033	*ANLN*	Anillin actin binding protein
TC0100013035.hg.1	3.72	5.02	-2.47	0.029	*ANO7P1*	Anoctamin 7 pseudogene 1
TC1100007363.hg.1	5.02	5.88	-1.82	0.002	*API5*	Apoptosis inhibitor 5
TC1300009714.hg.1	9.8	11.51	-3.26	0.018	*ARGLU1*	Arginine and glutamate rich 1
TC1500010707.hg.1	3.82	4.44	-1.53	0.004	*ARHGAP11B*	Rho GTPase activating protein 11B
TC1400007307.hg.1	7.41	8.02	-1.52	0.022	*ARID4A*	AT rich interactive domain 4A (RBP1-like)
TC1600011494.hg.1	7.47	8.68	-2.32	0.018	*ARL6IP1*	ADP-ribosylation factor like GTPase 6 interacting protein 1
TC0700013380.hg.1	5.1	5.75	-1.58	0.028	*ASL*	Argininosuccinate lyase
TC2000007117.hg.1	5.03	5.91	-1.84	0.024	*ASXL1*	Additional sex combs like transcriptional regulator 1
TC0300012000.hg.1	7.29	8.22	-1.91	0.027	*ATG3*	Autophagy related 3
TC0200012282.hg.1	5.21	6.11	-1.86	0.036	*ATL2*	Atlastin GTPase 2
TC0500012523.hg.1	4.47	5.11	-1.56	0.039	*ATOX1*	Antioxidant 1 copper chaperone
TC0900007451.hg.1	7.28	6.53	1.68	0.047	*ATP5A1P10*	ATP synthase, H+ transporting, mitochondrial F1 complex, alpha subunit 1 pseudogene 10
TC1200012798.hg.1	6.16	7.15	-1.98	0.005	*ATP5G2*	ATP synthase, H+ transporting, mitochondrial Fo complex subunit C2 (subunit 9)

TC1700009960.hg.1	7.26	6.6	1.58	0.030	*ATPAF2*	ATP synthase mitochondrial F1 complex assembly factor 2
TC2000009550.hg.1	5.14	6.08	-1.92	0.024	*AURKA*	Aurora kinase A
TC0700007905.hg.1	4.43	5.09	-1.59	0.037	*AUTS2*	Autism susceptibility candidate 2
TC0800012447.hg.1	7.4	9.84	-5.4	0.030	*AZIN1*	Antizyme inhibitor 1
TC0700007137.hg.1	3.72	5.62	-3.73	3E-04	*BBS9*	Bardet-Biedl syndrome 9
TSUnmapped00000366.hg.1	4.17	3.58	1.51	1E-04	*BCL2L14*	BCL2-like 14 (apoptosis facilitator)
TC1200006862.hg.1	5.8	6.53	-1.67	0.012	*BCL2L14*	BCL2-like 14 (apoptosis facilitator)
TC1000012522.hg.1	6.2	7.39	-2.29	0.013	*BEND7*	BEN domain containing 7
TC0700008454.hg.1	7.89	8.93	-2.06	0.032	*BHLHA15*	Basic helix-loop-helix family, member a15
TC0500012162.hg.1	5.25	6.71	-2.74	0.002	*BRD8*	Bromodomain containing 8
TC1300009458.hg.1	4.06	3.41	1.56	0.010	*BRK1P2*	BRICK1, SCAR/WAVE actin-nucleating complex subunit pseudogene 2 [Source:HGNC Symbol;Acc:HGNC:42021]
TC0600007307.hg.1	4.48	5.3	-1.77	0.030	*BTN2A1*	Butyrophilin, subfamily 2, member A1
TC1100012227.hg.1	5.99	5.4	1.5	0.047	*C11orf65*	Chromosome 11 open reading frame 65
TC1200008370.hg.1	5.39	7.15	-3.39	0.048	*C12orf29*	Chromosome 12 open reading frame 29
TC1400006674.hg.1	3.55	4.25	-1.62	0.015	*C14orf119*	Chromosome 14 open reading frame 119
TC1600008144.hg.1	3.44	4.53	-2.13	0.004	*C16orf70*	Chromosome 16 open reading frame 70
TC0100017722.hg.1	4.37	5.87	-2.83	0.038	*C1orf131*	Chromosome 1 open reading frame 131
TC0200016590.hg.1	4.1	4.91	-1.75	0.024	*C2orf47*	Chromosome 2 open reading frame 47
TC0300007657.hg.1	4.14	3.43	1.63	0.003	*C3orf14*	Chromosome 3 open reading frame 14
TC0600011487.hg.1	4.6	3.97	1.55	0.038	*C6orf10*	Chromosome 6 open reading frame 10
TC0900009253.hg.1	5.71	6.34	-1.55	0.014	*C9orf142*	Chromosome 9 open reading frame 142
TC0100010686.hg.1	4.61	6.2	-3.02	0.001	*CACYBP*	Calcyclin binding protein
TC0100014201.hg.1	3.35	3.97	-1.54	0.002	*CC2D1B*	Coiled-coil and C2 domain containing 1B
TC0500009787.hg.1	3.89	4.96	-2.11	0.007	*CCDC127*	Coiled-coil domain containing 127
TC1100010397.hg.1	7.13	8.23	-2.14	0.014	*CCDC34*	Coiled-coil domain containing 34
TC1500010869.hg.1	3.91	3.3	1.53	0.034	*CCPG1; MIR628*	Cell cycle progression 1; microRNA 628
TC1300007161.hg.1	4.87	4.21	1.58	0.048	*CDADC1*	Cytidine and dCMP deaminase domain containing 1
TC0100009215.hg.1	4.88	4.26	1.53	0.003	*CDC14A*	Cell division cycle 14A
TC2000006559.hg.1	4.3	4.99	-1.62	0.038	*CDC25B*	Cell division cycle 25B
TC1900008161.hg.1	4.65	5.38	-1.66	0.008	*CEACAM3*	Carcinoembryonic antigen-related cell adhesion molecule 3

TC1100010741.hg.1	7.04	8.15	-2.15	0.045	*CELF1*	CUGBP, Elav-like family member 1
TC1100008847.hg.1	4.66	5.47	-1.75	0.001	*CEP57*	Centrosomal protein 57kDa
TC1700008603.hg.1	4.86	5.66	-1.74	0.005	*CEP95*	Centrosomal protein 95kDa
TC2000006868.hg.1	3.89	4.51	-1.54	0.006	*CFAP61*	Cilia and flagella associated protein 61
TC1400007204.hg.1	3.48	4.13	-1.57	0.046	*CGRRF1*	Cell growth regulator with ring finger domain 1
TC1200009742.hg.1	5.36	6.34	-1.98	0.036	*CHD4*	Chromodomain helicase DNA binding protein 4
TC0X00010164.hg.1	5.03	6.04	-2.02	0.046	*CHMP1B2P*	Charged multivesicular body protein 1B2, pseudogene
TC0200016703.hg.1	7.04	7.9	-1.82	0.029	*CHMP3*	Charged multivesicular body protein 3
TC0100010100.hg.1	6.17	7.75	-2.99	0.041	*CKS1B*	CDC28 protein kinase regulatory subunit 1B
TC1800007680.hg.1	10.08	11.32	-2.37	0.034	*CNDP2*	CNDP dipeptidase 2 (metallopeptidase M20 family)
TC1000008758.hg.1	4.48	5.89	-2.65	0.029	*CNNM2*	Cyclin and CBS domain divalent metal cation transport mediator 2
TC2100007446.hg.1	6.7	8.03	-2.52	0.013	*COL6A1*	Collagen, type VI, alpha 1
TC0300008820.hg.1	5.8	4.79	2.01	0.019	*COL6A6*	Collagen, type VI, alpha 6
TC0300012596.hg.1	5.54	6.2	-1.58	0.016	*COPB2*	Coatomer protein complex subunit beta 2 (beta prime)
TC1100006772.hg.1	3.39	4	-1.53	0.005	*COX6CP5*	Cytochrome c oxidase subunit VIc pseudogene 5 [Source:HGNC Symbol;Acc:HGNC:42791]
TC0500009488.hg.1	3.13	3.86	-1.65	0.002	*CREBRF*	CREB3 regulatory factor
TC2000007627.hg.1	6.21	8	-3.46	0.009	*CSE1L*	CSE1 chromosome segregation 1-like (yeast)
TC0100015754.hg.1	6.08	7.44	-2.57	0.028	*CTSK*	Cathepsin K
TC0700009585.hg.1	3.51	4.18	-1.59	0.004	*CUL1*	Cullin 1
TC1100012208.hg.1	3.39	4.09	-1.62	0.024	*CWF19L2*	CWF19-like 2, cell cycle control (S. pombe)
TC1100007699.hg.1	4.65	3.81	1.79	0.006	*CYCSP26*	Cytochrome c, somatic pseudogene 26
TC1900011679.hg.1	4.26	4.95	-1.62	0.004	*CYP4F3*	Cytochrome P450, family 4, subfamily F, polypeptide 3
TC0400010167.hg.1	5.04	5.73	-1.62	0.044	*DCAF16*	DDB1 and CUL4 associated factor 16
TC1700012089.hg.1	4.98	5.74	-1.69	0.014	*DCXR*	Dicarbonyl/L-xylulose reductase
TC0800007330.hg.1	4.21	4.82	-1.53	0.001	*DDHD2*	DDHD domain containing 2
TC1600011424.hg.1	3.13	3.76	-1.55	0.029	*DDX19B*	DEAD (Asp-Glu-Ala-Asp) box polypeptide 19B
TC1700010501.hg.1	3.89	4.59	-1.62	0.026	*DDX52*	DEAD (Asp-Glu-Ala-Asp) box polypeptide 52
TC0800009486.hg.1	3.34	3.99	-1.57	0.041	*DEFB107A; DEFB107B*	Defensin, beta 107A; defensin, beta 107B
TC1100007220.hg.1	4.1	5.61	-2.86	0.004	*DEPDC7*	DEP domain containing 7
TC0600011375.hg.1	3.74	4.33	-1.51	3E-04	*DHX16*	DEAH (Asp-Glu-Ala-His) box polypeptide 16

TC1100009043.hg.1	6.13	5.39	1.67	0.023	*DIXDC1*	DIX domain containing 1
TC0500008431.hg.1	5.01	6.22	-2.31	0.028	*DMXL1*	Dmx-like 1
TC1000010990.hg.1	4.53	5.28	-1.68	0.011	*DNAJB12*	DnaJ (Hsp40) homolog, subfamily B, member 12
TC0200010840.hg.1	4.55	5.8	-2.38	0.037	*DNAJB2*	DnaJ (Hsp40) homolog, subfamily B, member 2
TC1000007842.hg.1	6.07	5.35	1.65	0.017	*DNAJC19P1*	DnaJ heat shock protein family (Hsp40) member C19 pseudogene 1 [Source:HGNC Symbol;Acc:HGNC:45064]
TC0700012122.hg.1	4.9	6.25	-2.56	0.044	*DNAJC2*	DnaJ (Hsp40) homolog, subfamily C, member 2
TC1200007277.hg.1	6.14	7.07	-1.91	0.027	*DNM1L*	Dynamin 1-like
TC0700008755.hg.1	4.12	3.48	1.56	0.033	*DUS4L*	Dihydrouridine synthase 4-like
TC1400006461.hg.1	8.41	9.96	-2.93	0.002	*DUXAP10; LINC01296*	Double homeobox A pseudogene 10; long intergenic non-protein coding RNA 1296
TC0200010567.hg.1	5.6	6.62	-2.03	0.046	*EEF1B2; SNORA41*	Eukaryotic translation elongation factor 1 beta 2; small nucleolar RNA, H/ACA box 41
TC0100012242.hg.1	4.53	3.85	1.61	0.013	*EFCAB2*	EF-hand calcium binding domain 2
TC0100010111.hg.1	3.85	4.51	-1.58	0.030	*EFNA1*	Ephrin-A1
TC1900011736.hg.1	5.03	6.15	-2.17	0.017	*EGLN2*	Egl-9 family hypoxia-inducible factor 2
TC1100011190.hg.1	6.28	7.03	-1.68	0.029	*EHD1*	EH domain containing 1
TC1000011431.hg.1	4.85	5.65	-1.74	0.044	*EIF2S2P3*	Eukaryotic translation initiation factor 2, subunit 2 beta pseudogene 3
TSUnmapped00000397.hg.1	5.91	6.88	-1.96	0.015	*EIF3F*	Eukaryotic translation initiation factor 3 subunit F [Source:UniProtKB/Swiss-Prot;Acc:O00303]
TC0800008531.hg.1	4.75	5.36	-1.53	0.027	*EMC2*	ER membrane protein complex subunit 2
TC0100015743.hg.1	4.26	5.46	-2.3	0.031	*ENSA*	Endosulfine alpha
TC0800008546.hg.1	4.13	5.27	-2.21	0.032	*ENY2*	Enhancer of yellow 2 homolog (Drosophila)
TC1200010038.hg.1	5.35	4.55	1.74	0.012	*EPS8*	Epidermal growth factor receptor pathway substrate 8
TC2000007219.hg.1	3.72	4.35	-1.54	0.044	*ERGIC3*	ERGIC and golgi 3
TC0100016035.hg.1	4.89	5.69	-1.74	0.049	*ETV3*	Ets variant 3
TC0700012959.hg.1	4.31	5.18	-1.83	0.025	*EZH2*	Enhancer of zeste 2 polycomb repressive complex 2 subunit
TC0500009591.hg.1	5.79	7.17	-2.6	4E-04	*FAF2*	Fas associated factor family member 2
TC2200007614.hg.1	4.94	5.67	-1.66	0.030	*FAM118A*	Family with sequence similarity 118, member A
TC0X00009737.hg.1	4.71	5.9	-2.28	0.019	*FAM156A*	Family with sequence similarity 156, member A
TC0900009333.hg.1	3.69	4.58	-1.86	0.002	*FAM157B*	Family with sequence similarity 157, member B
TC0400011208.hg.1	4.21	5.06	-1.81	0.036	*FAM175A*	Family with sequence similarity 175, member A
TC0600013007.hg.1	5	4.01	1.98	0.004	*FAM184A*	Family with sequence similarity 184, member A
TC0500012980.hg.1	6.25	6.93	-1.6	0.001	*FAM193B*	Family with sequence similarity 193, member B

TC1800008162.hg.1	3.66	4.77	-2.16	0.001	*FAM210A*	Family with sequence similarity 210, member A
TC0100015550.hg.1	4.36	5.12	-1.69	0.016	*FAM72C*	Family with sequence similarity 72, member C
TC0800006646.hg.1	4.27	4.89	-1.54	0.030	*FAM86B3P*	Family with sequence similarity 86, member A pseudogene
TC0300008652.hg.1	4.41	5.82	-2.66	0.016	*FAM86JP*	Family with sequence similarity 86, member A pseudogene
TC1900010675.hg.1	6.16	7.15	-1.98	0.017	*FBL*	Fibrillarin
TC1200009489.hg.1	3.5	4.14	-1.56	0.003	*FBRSL1*	Fibrosin-like 1
TC0800012396.hg.1	3.57	4.35	-1.72	0.018	*FBXO16*	F-box protein 16
TC1900008543.hg.1	5.51	6.11	-1.51	0.040	*FCGRT*	Fc fragment of IgG, receptor, transporter, alpha
TC0400012795.hg.1	6.25	7.18	-1.91	0.021	*FIP1L1*	Factor interacting with PAPOLA and CPSF1
TC0900011252.hg.1	3.8	4.39	-1.51	0.043	*FKBP15*	FK506 binding protein 15
TC1600009535.hg.1	7.55	8.17	-1.53	0.047	*FOPNL*	FGFR1OP N-terminal like
TC2000007058.hg.1	5.05	6.39	-2.52	0.008	*FRG1BP*	FSHD region gene 1 family member B, pseudogene
TC0200012078.hg.1	4.7	5.71	-2.01	0.019	*FTH1P3*	Ferritin, heavy polypeptide 1 pseudogene 3
TC1600008454.hg.1	5.65	5.05	1.51	0.046	*GABARAPL2*	GABA(A) receptor-associated protein like 2
TC0300011740.hg.1	3.28	3.87	-1.5	0.019	*GAPDHP50*	Glyceraldehyde 3 phosphate dehydrogenase pseudogene 50 [Source:HGNC Symbol;Acc:HGNC:38556]
TC1900007436.hg.1	3.94	5.24	-2.46	0.016	*GATAD2A; MIR640*	GATA zinc finger domain containing 2A; microRNA 640
TC1600011573.hg.1	3.98	4.66	-1.6	0.007	*GCSH*	Glycine cleavage system protein H (aminomethyl carrier)
TC1700007638.hg.1	4.32	5.03	-1.63	0.017	*GGNBP2*	Gametogenetin binding protein 2
TC2200009236.hg.1	5.87	6.8	-1.9	0.011	*GGT1*	Gamma-glutamyltransferase 1
TC1200010844.hg.1	5.1	5.98	-1.85	0.025	*GLYCAM1*	Glycosylation dependent cell adhesion molecule 1 (pseudogene)
TC0100013748.hg.1	3.39	4.04	-1.57	0.003	*GNL2*	Guanine nucleotide binding protein-like 2 (nucleolar)
TC1500010846.hg.1	4.53	5.24	-1.64	0.035	*GOLGA8K; ULK4P1*	Golgin A8 family, member K; ULK4 pseudogene 1
TC0500012378.hg.1	4.9	4.25	1.56	0.005	*GPR151*	G protein-coupled receptor 151
TC1900007829.hg.1	3.7	4.45	-1.68	0.015	*GRAMD1A*	GRAM domain containing 1A
TC1600009422.hg.1	3.99	4.59	-1.51	0.030	*GSPT1*	G1 to S phase transition 1
TC1200009250.hg.1	4.96	5.75	-1.72	0.003	*GTF2H3*	General transcription factor IIH subunit 3
TC1000006464.hg.1	4.25	5.01	-1.69	0.006	*GTPBP4*	GTP binding protein 4
TC0700008747.hg.1	3.87	5.05	-2.27	0.006	*HBP1*	HMG-box transcription factor 1
TC1500006675.hg.1	8.32	9.54	-2.33	0.031	*HERC2P9*	Hect domain and RLD 2 pseudogene 9
TC2000007232.hg.1	4.25	4.93	-1.6	0.049	*HIGD1AP16*	HIG1 hypoxia inducible domain family member 1A pseudogene 16 [Source:HGNC Symbol;Acc:HGNC:43011]

TC1100009241.hg.1	5.85	7.55	-3.24	0.026	*HINFP*	Histone H4 transcription factor
TC0600007273.hg.1	4.61	5.4	-1.74	0.046	*HIST1H1E*	Histone cluster 1, H1e
TC0100009876.hg.1	5.38	6.57	-2.28	0.004	*HIST2H2AA3; HIST2H2AA4*	Histone cluster 2, H2aa3; histone cluster 2, H2aa4
TC0100018482.hg.1	5.82	6.93	-2.16	0.013	*HIST2H2AA4; HIST2H2AA3*	Histone cluster 2, H2aa4; histone cluster 2, H2aa3
TC0100015701.hg.1	3.79	4.4	-1.53	0.001	*HIST2H3A; HIST2H3C*	Histone cluster 2, H3a; histone cluster 2, H3c
TC0600011501.hg.1	5.38	4.79	1.51	0.005	*HLA-DQB3*	Major histocompatibility complex, class II, DQ beta 3
TC0200008560.hg.1	4.98	5.57	-1.51	0.007	*HMGN1P36*	High mobility group nucleosome binding domain 1 pseudogene 36 [Source:HGNC Symbol;Acc:HGNC:39380]
TC1100008414.hg.1	5.4	6.09	-1.61	0.030	*HNRNPA1P40*	Heterogeneous nuclear ribonucleoprotein A1 pseudogene 40 [Source:HGNC Symbol;Acc:HGNC:48769]
TC1300009161.hg.1	7.5	8.19	-1.62	0.046	*HNRNPA3P5*	Heterogeneous nuclear ribonucleoprotein A3 pseudogene 5 [Source:HGNC Symbol;Acc:HGNC:39774]
TSUnmapped00000765.hg.1	4.91	4.25	1.58	0.008	*HNRNPCL4*	Heterogeneous nuclear ribonucleoprotein C-like 4 [Source:HGNC Symbol;Acc:HGNC:51333]
TC1000010438.hg.1	8.56	10.04	-2.79	0.038	*HNRNPF*	Heterogeneous nuclear ribonucleoprotein F
TC1200012638.hg.1	4.02	5.6	-2.98	0.001	*HOXC9*	Homeobox C9
TC0600009322.hg.1	3.91	4.51	-1.52	0.009	*HSF2*	Heat shock transcription factor 2
TC2100008306.hg.1	3.65	3.04	1.53	0.021	*HSF2BP*	Heat shock transcription factor 2 binding protein
TC0400009618.hg.1	5.36	5.95	-1.5	0.015	*HSP90AA4P*	Heat shock protein 90kDa alpha family class A member 4, pseudogene [Source:HGNC Symbol;Acc:HGNC:5255]
TC0600007776.hg.1	6.46	5.72	1.67	0.011	*HSPE1P11*	Heat shock protein family E (Hsp10) member 1 pseudogene 11 [Source:HGNC Symbol;Acc:HGNC:49330]
TC1100012472.hg.1	5.57	4.85	1.65	0.026	*HSPE1P18*	Heat shock protein family E (Hsp10) member 1 pseudogene 18 [Source:HGNC Symbol;Acc:HGNC:49337]
TC0300008782.hg.1	3.26	4.21	-1.93	0.014	*IFT122*	Intraflagellar transport 122
TC1400010516.hg.1	7.18	6.47	1.63	0.042	*IGHVIII-44*	Immunoglobulin heavy variable (III)-44 (pseudogene)
TC0200008393.hg.1	5.45	6.19	-1.66	0.026	*IGKV3D-20*	Immunoglobulin kappa variable 3D-20
TC0200008408.hg.1	4.27	4.95	-1.6	0.003	*IGKV3D-7*	Immunoglobulin kappa variable 3D-7
TC2200009214.hg.1	4.16	4.82	-1.58	0.013	*IGLV2-18*	Immunoglobulin lambda variable 2-18
TC1100007833.hg.1	4.12	4.73	-1.52	0.032	*INCENP*	Inner centromere protein
TC1300007791.hg.1	3.5	4.1	-1.51	0.003	*IPO5*	Importin 5
TC1600011427.hg.1	4.06	4.77	-1.64	0.020	*IST1*	Increased sodium tolerance 1 homolog (yeast)
TSUnmapped00000492.hg.1	4.04	4.75	-1.64	0.015	*KAT6B*	K(lysine) acetyltransferase 6B
TC1600006628.hg.1	3.25	4.03	-1.71	0.016	*KCTD5*	Potassium channel tetramerization domain containing 5
TC0300007223.h	3.33	4.3	-1.96	2E-04	*KIF15*	Kinesin family member 15

g.1						
TC1100010418.hg.1	3.34	3.95	-1.53	0.001	*KIF18A*	Kinesin family member 18A
TC0500008777.hg.1	4.39	5.08	-1.61	0.025	*KIF20A*	Kinesin family member 20A
TC1600007425.hg.1	3.23	3.9	-1.59	0.001	*KIF22*	Kinesin family member 22
TC1500007699.hg.1	4.36	5.03	-1.59	0.038	*KIF23*	Kinesin family member 23
TC0900010582.hg.1	3.63	4.33	-1.62	0.010	*KIF27*	Kinesin family member 27
TC1100011374.hg.1	4.45	5.5	-2.07	0.008	*KMT5B*	Lysine (K)-specific methyltransferase 5B
TC1800006897.hg.1	3.94	4.62	-1.61	0.009	*LAMA3*	Laminin, alpha 3
TSUnmapped00000355.hg.1	3.57	2.97	1.51	0.009	*LEUTX*	Leucine twenty homeobox
TC1400007666.hg.1	4.29	4.99	-1.62	0.001	*LIN52*	Lin-52 DREAM MuvB core complex component
TC0400006920.hg.1	5.68	4.96	1.64	0.003	*LINC01085*	Long intergenic non-protein coding RNA 1085
TC1400008546.hg.1	8.4	9.91	-2.85	0.006	*LINC01296; DUXAP10*	Long intergenic non-protein coding RNA 1296; double homeobox A pseudogene 10
TC0800007715.hg.1	5.58	4.99	1.51	0.023	*LINC01606; RP11-513O17.2; plyshoy*	Homo sapiens long intergenic non-protein coding RNA 1606 (LINC01606), long non-coding RNA.; novel transcript; Transcript Identified by AceView; long intergenic non-protein coding RNA 1606 [Source:HGNC Symbol;Acc:HGNC:51656]
TC1700007238.hg.1	4.42	3.57	1.8	0.019	*LOC100287072*	Ribosomal protein S6 kinase, 70kDa, polypeptide 1 pseudogene
TC0900007768.hg.1	4.33	5.15	-1.76	0.035	*LOC389765*	Kinesin family member 27 pseudogene
TC0400012837.hg.1	7.89	8.72	-1.78	3E-04	*LOC645513*	Uncharacterized LOC645513; Salzman2013 ANNOTATED, ncRNA, OVEXON best transcript NR_037630; Salzman2013 ANNOTATED, ncRNA, OVCODE, OVERLAPTX, OVEXON best transcript NR_037630; Salzman2013 ANNOTATED, INTERNAL, ncRNA, OVERLAPTX, OVEXON best transcript NR_037630; Salzman2013 ANNOTATED, INTERNAL, ncRNA, OVEXON best transcript NR_037630; Transcript Identified by AceView, Entrez Gene ID(s) 645513
TC1600007141.hg.1	5.33	7.23	-3.73	0.042	*LOC81691; AC004381.6; U4atac*	Exonuclease NEF-sp; Putative RNA exonuclease NEF-sp [Source:UniProtKB/Swiss-Prot;Acc:Q96IC2]; Salzman2013 ANNOTATED, CDS, coding, OVCODE, OVERLAPTX, OVEXON, UTR3, UTR5 best transcript NM_001199053; Salzman2013 ANNOTATED, CDS, coding, INTERNAL, OVCODE, OVERLAPTX, OVEXON best transcript NM_001199053; Salzman2013 ANNOTATED, CDS, coding, OVCODE, OVERLAPTX, OVEXON, UTR3 best transcript NM_001199053; Transcript Identified by AceView, Entrez Gene ID(s) 81691; U4atac minor spliceosomal RNA [Source:RFAM;Acc:RF00618]
TC0900011192.hg.1	4.83	5.59	-1.69	0.002	*LPAR1*	Lysophosphatidic acid receptor 1

TC1200008344.hg.1	5.6	4.82	1.72	0.005	*LRRIQ1*	Leucine-rich repeats and IQ motif containing 1
TC1700010810.hg.1	6.45	7.28	-1.77	0.032	*LSM12*	LSM12 homolog
TC0200013861.hg.1	4.12	4.82	-1.62	0.011	*MALL*	Mal, T-cell differentiation protein-like
TC0500013277.hg.1	3.98	4.85	-1.83	3E-04	*MAML1*	Mastermind-like transcriptional coactivator 1
TC2000007193.hg.1	4.15	4.78	-1.55	4E-04	*MAP1LC3A*	Microtubule-associated protein 1 light chain 3 alpha
TC0200008259.hg.1	4.41	5.39	-1.98	0.016	*MAT2A*	Methionine adenosyltransferase II, alpha
TC0300013334.hg.1	6.67	7.57	-1.86	0.025	*MCCC1*	Methylcrotonoyl-CoA carboxylase 1
TC0600010867.hg.1	4.64	5.73	-2.12	0.002	*MCUR1*	Mitochondrial calcium uniporter regulator 1
TC0600013177.hg.1	6.38	7.68	-2.46	0.043	*MED23*	Mediator complex subunit 23
TC1500008521.hg.1	5.1	6.23	-2.19	0.019	*MEF2A*	Myocyte enhancer factor 2A
TC0700008542.hg.1	3.9	4.67	-1.7	0.006	*MEPCE*	Methylphosphate capping enzyme
TC1900011845.hg.1	3.8	4.57	-1.71	0.002	*MFSD12*	Major facilitator superfamily domain containing 12
TC0500010813.hg.1	5.33	7.15	-3.54	0.013	*MIER3*	Mesoderm induction early response 1, family member 3
TC1200006661.hg.1	4.5	5.85	-2.54	0.003	*MIR141*	MicroRNA 141
TC0700008475.hg.1	7.06	8.59	-2.9	0.025	*MIR3609*	MicroRNA 3609
TC1900008799.hg.1	4.53	3.93	1.51	0.034	*MIR516B1*	MicroRNA 516b-1
TC0500011685.hg.1	4.59	5.69	-2.15	0.004	*MIR548F3*	MicroRNA 548f-3
TC1600011458.hg.1	6.34	7.56	-2.34	0.027	*MIR6859-2; MIR6859-4; WASH4P*	MicroRNA 6859-2; microRNA 6859-4; WAS protein family homolog 4 pseudogene
TC1800008086.hg.1	7.77	7.14	1.55	0.036	*MIR7153*	MicroRNA 7153
TC0100015999.hg.1	6.03	5.27	1.69	0.010	*MIR9-1*	MicroRNA 9-1
TC2000008792.hg.1	4.45	3.59	1.82	0.036	*MLLT10P1*	Myeloid/lymphoid or mixed-lineage leukemia; translocated to, 10 pseudogene 1
TC0200016420.hg.1	5.55	6.82	-2.41	0.007	*MRPL33*	Mitochondrial ribosomal protein L33
TC1200009735.hg.1	6.25	7.89	-3.12	0.031	*MRPL51*	Mitochondrial ribosomal protein L51
TC0500007314.hg.1	5.77	7.26	-2.79	0.029	*MRPS30*	Mitochondrial ribosomal protein S30
TC0900012153.hg.1	4.05	4.71	-1.58	0.015	*MSANTD3*	Myb/SANT-like DNA-binding domain containing 3
TC1100012186.hg.1	3.76	4.89	-2.19	0.021	*MSANTD4*	Myb/SANT-like DNA-binding domain containing 4 with coiled-coils
TC0600007610.hg.1	4.78	6.22	-2.72	0.010	*MSH5; MSH5-SAPCD1; SAPCD1*	MutS homolog 5; MSH5-SAPCD1 readthrough (NMD candidate); suppressor APC domain containing 1
TC1700008378.hg.1	3.87	4.99	-2.16	0.047	*MSI2*	Musashi RNA binding protein 2
TC1700007779.hg.1	5.31	6.18	-1.82	0.010	*MSL1*	Male-specific lethal 1 homolog (Drosophila)

TC0100015144.hg.1	4.3	3.68	1.54	0.004	*MTATP6P14*	Mitochondrially encoded ATP synthase 6 pseudogene 14 [Source:HGNC Symbol;Acc:HGNC:52059]
TC0200009292.hg.1	3.93	3.16	1.71	0.008	*MTCO2P7*	MT-CO2 pseudogene 7 [Source:HGNC Symbol;Acc:HGNC:52023]
TC1300008439.hg.1	5.19	5.81	-1.54	0.022	*MTIF3*	Mitochondrial translational initiation factor 3
TC1500008893.hg.1	5.02	4.25	1.71	0.024	*MTMR10*	Myotubularin related protein 10
TC0300006552.hg.1	8.34	9.04	-1.62	0.016	*MTMR14*	Myotubularin related protein 14
TC0400007481.hg.1	4.74	6.2	-2.76	0.007	*MTND3P22*	Mitochondrially encoded NADH:ubiquinone oxidoreductase core subunit 3 pseudogene 22 [Source:HGNC Symbol;Acc:HGNC:52168]
TC0X00008980.hg.1	7.64	7.04	1.52	0.027	*MTND6P12*	Mitochondrially encoded NADH:ubiquinone oxidoreductase core subunit 6 pseudogene 12 [Source:HGNC Symbol;Acc:HGNC:39475]
TC0800009735.hg.1	14.08	15.14	-2.08	0.033	*MTUS1*	Microtubule associated tumor suppressor 1
TC0200010284.hg.1	4.66	5.64	-1.97	0.007	*MYO1B*	Myosin IB
TC0200008622.hg.1	7.78	7.16	1.54	0.006	*NANOGNBP1*	NANOGNB pseudogene 1
TC1200012550.hg.1	6.73	5.97	1.69	0.023	*NANOGNBP2*	NANOGNB pseudogene 2 [Source:HGNC Symbol;Acc:HGNC:42159]
TC1100009819.hg.1	6.13	8.08	-3.86	0.020	*NAP1L4*	Nucleosome assembly protein 1-like 4
TC0100009770.hg.1	6.08	6.68	-1.52	0.012	*NBPF12*	Neuroblastoma breakpoint family, member 12
TC2000010002.hg.1	3.16	4.12	-1.95	0.029	*NCOA6*	Nuclear receptor coactivator 6
TC1900011795.hg.1	5.4	6.47	-2.1	0.047	*NDUFA3*	NADH dehydrogenase (ubiquinone) 1 alpha subcomplex, 3, 9kDa
TC0900009655.hg.1	5.98	5.39	1.51	0.002	*NDUFA5P3*	NADH:ubiquinone oxidoreductase subunit A5 pseudogene 3 [Source:HGNC Symbol;Acc:HGNC:48845]
TC1700007478.hg.1	4.47	6.13	-3.17	0.001	*NF1*	Neurofibromin 1
TC1600007374.hg.1	7.73	8.93	-2.31	0.018	*NFATC2IP; MIR4517*	Nuclear factor of activated T-cells, cytoplasmic, calcineurin-dependent 2 interacting protein; microRNA 4517
TC1300009148.hg.1	3.61	4.82	-2.32	0.004	*NFYAP1*	Nuclear transcription factor Y subunit alpha pseudogene 1 [Source:HGNC Symbol;Acc:HGNC:34078]
TC1000008961.hg.1	5.91	6.62	-1.64	0.010	*NHLRC2*	NHL repeat containing 2
TC1600008226.hg.1	3.99	4.78	-1.73	0.017	*NIP7*	NIP7, nucleolar pre-rRNA processing protein
TC0100009626.hg.1	3.95	3.33	1.53	0.003	*NOTCH2P1*	Notch 2 pseudogene 1 [Source:HGNC Symbol;Acc:HGNC:44253]
TC1600011355.hg.1	5.13	6.93	-3.49	0.036	*NPIPA1*	Nuclear pore complex interacting protein family, member A1
TC1600011354.hg.1	5.25	7.19	-3.83	0.035	*NPIPA2*	Nuclear pore complex interacting protein family, member A2
TC1600011353.hg.1	5.49	7.48	-3.98	0.035	*NPIPA3*	Nuclear pore complex interacting protein family, member A3
TC1600011364.hg.1	9.27	10.32	-2.06	0.039	*NPIPB5*	Nuclear pore complex interacting protein family, member B5
TC1600011365.hg.1	6.59	8.08	-2.81	0.045	*NPIPB9*	Nuclear pore complex interacting protein family, member B9

TC0800010553.hg.1	5.29	6.77	-2.79	0.011	*NSMAF*	Neutral sphingomyelinase activation associated factor
TC1600009829.hg.1	4.44	3.73	1.64	0.028	*NSMCE1*	NSE1 homolog, SMC5-SMC6 complex component
TC0Y00006563.hg.1	5.67	4.97	1.62	0.028	*OFD1P3Y*	OFD1 pseudogene 3, Y-linked [Source:HGNC Symbol;Acc:HGNC:23875]
TC0700009520.hg.1	4.04	3.44	1.52	0.001	*OR2A41P*	Olfactory receptor, family 2, subfamily A, member 41 pseudogene
TC0200008430.hg.1	5.3	4.67	1.55	0.006	*PABPC1P6*	Poly(A) binding protein, cytoplasmic 1 pseudogene 6 [Source:HGNC Symbol;Acc:HGNC:37986]
TC0400011130.hg.1	4.72	4.12	1.51	0.001	*PAQR3*	Progestin and adipoQ receptor family member III
TC1400006513.hg.1	6.04	6.76	-1.65	0.030	*PARP2*	Poly(ADP-ribose) polymerase 2
TC1700010540.hg.1	4.07	4.89	-1.76	0.004	*PCGF2*	Polycomb group ring finger 2
TC0300013864.hg.1	5.32	6.08	-1.69	0.017	*PDIA5; MIR7110*	Protein disulfide isomerase family A, member 5; microRNA 7110
TC1600007007.hg.1	7.93	8.63	-1.62	0.042	*PDXDC1*	Pyridoxal-dependent decarboxylase domain containing 1
TC1600010740.hg.1	7.4	9.05	-3.15	0.009	*PDXDC2P*	Pyridoxal-dependent decarboxylase domain containing 2, pseudogene
TC2000007792.hg.1	4.18	5.12	-1.92	0.028	*PFDN4*	Prefoldin subunit 4
TC1100013016.hg.1	5.96	4.27	3.24	0.048	*PGA4*	Pepsinogen 4, group I (pepsinogen A)
TC1700010222.hg.1	8.44	7.83	1.52	0.022	*PHF12*	PHD finger protein 12
TC1000010022.hg.1	5.16	6.08	-1.88	0.026	*PIP4K2A*	Phosphatidylinositol-5-phosphate 4-kinase, type II, alpha
TC1400007495.hg.1	4.61	6.68	-4.21	0.038	*PLEKHH1*	Pleckstrin homology domain containing, family H (with MyTH4 domain) member 1
TC1400006970.hg.1	9.65	10.26	-1.53	0.001	*PNN*	Pinin, desmosome associated protein
TC0700013605.hg.1	5.37	4.58	1.74	0.033	*PNPLA8*	Patatin-like phospholipase domain containing 8
TC0200012652.hg.1	4.63	5.41	-1.72	0.022	*PNPT1*	Polyribonucleotide nucleotidyltransferase 1
TC0700010910.hg.1	3.94	4.57	-1.56	0.009	*POLD2*	Polymerase (DNA directed), delta 2, accessory subunit
TC0700011575.hg.1	5.94	6.76	-1.77	0.032	*POMZP3*	POM121 and ZP3 fusion
TC0200009331.hg.1	3.89	4.79	-1.86	0.020	*POTEE*	POTE ankyrin domain family, member E
TC0100009651.hg.1	5.37	6.16	-1.73	0.002	*PPIAL4A*	Peptidylprolyl isomerase A (cyclophilin A)-like 4A
TC0100007920.hg.1	5.56	6.41	-1.8	0.025	*PPIE*	Peptidylprolyl isomerase E (cyclophilin E)
TC0200007411.hg.1	5.07	4.31	1.69	0.007	*PPM1B*	Protein phosphatase, Mg2+/Mn2+ dependent, 1B
TC1100011157.hg.1	9.18	10.36	-2.27	0.048	*PPP1R14B*	Protein phosphatase 1, regulatory (inhibitor) subunit 14B
TC0400010585.hg.1	4.41	3.8	1.52	0.004	*PRDX4P1*	Peroxiredoxin 4 pseudogene 1 [Source:HGNC Symbol;Acc:HGNC:43598]
TC0200015080.hg.1	4.11	4.78	-1.59	0.018	*PRKRA*	Protein kinase, interferon-inducible double stranded RNA dependent activator
TC0100009902.hg.1	8.66	10.37	-3.29	0.004	*PRPF3*	Pre-mRNA processing factor 3
TC0X00011278.hg.1	4.56	5.35	-1.72	0.037	*PRRG1*	Proline rich Gla (G-carboxyglutamic acid) 1

TC1100006441.hg.1	7.61	8.72	-2.17	0.014	*PSMD13*	Proteasome 26S subunit, non-ATPase 13
TC1000009296.hg.1	4.28	5.16	-1.84	0.014	*PTPRE*	Protein tyrosine phosphatase, receptor type, E
TC0200012727.hg.1	3.27	4.02	-1.68	0.038	*PUS10*	Pseudouridylate synthase 10
TC0800008848.hg.1	4.74	5.67	-1.91	0.026	*PVT1; MIR1204*	Pvt1 oncogene (non-protein coding); microRNA 1204
TC2000009877.hg.1	4.06	3.48	1.5	0.006	*RAD21L1*	RAD21 cohesin complex component like 1
TC1400008945.hg.1	7.25	9.29	-4.11	0.016	*RALGAPA1*	Ral GTPase activating protein, alpha subunit 1 (catalytic)
TC2000009909.hg.1	3.73	4.93	-2.29	0.044	*RALY*	RALY heterogeneous nuclear ribonucleoprotein
TC0100008607.hg.1	5.01	6.01	-2	0.004	*RAVER2*	Ribonucleoprotein, PTB-binding 2
TC2000008985.hg.1	7.95	10.03	-4.24	0.012	*RBM39*	RNA binding motif protein 39
TC0X00011401.hg.1	6.25	8.28	-4.07	0.006	*RBMX; SNORD61*	RNA binding motif protein, X-linked; small nucleolar RNA, C/D box 61
TC0600006864.hg.1	4.64	5.23	-1.51	0.021	*RIOK1*	RIO kinase 1
TC0200016705.hg.1	5.7	6.72	-2.02	0.011	*RNF103-CHMP3; CHMP3*	RNF103-CHMP3 readthrough; charged multivesicular body protein 3
TC0800009714.hg.1	4.37	3.78	1.5	0.023	*RP11-13N12.1; storbly*	Novel transcript; Transcript Identified by AceView
TC1700008493.hg.1	4.93	4.35	1.5	0.001	*RP11-15E18.1; slarvoybu*	Novel transcript, antisense to APPBP2; Transcript Identified by AceView
TC0100018384.hg.1	5.61	6.26	-1.58	0.047	*RP11-206L10.10; TCONS_l2_00000968*	Jeck2013 ALT_ACCEPTOR, INTERNAL, ncRNA, OVERLAPTX, OVEXON best transcript TCONS_l2_00000968
TC0100018474.hg.1	4.57	5.78	-2.33	0.012	*RP11-403I13.8; LINC00869*	Long intergenic non-protein coding RNA 869 [Source:EntrezGene;Acc:57234]
TC0400006967.hg.1	5.85	5.19	1.58	0.013	*RP11-446J8.1; skoygu*	Novel transcript; Transcript Identified by AceView
TC1700009927.hg.1	5.52	6.17	-1.56	0.007	*RP11-45M22.5*	Novel transcript
TC0300012845.hg.1	3.82	4.48	-1.57	8.31E-05	*RP11-529G21.2; rawfeebu*	Novel transcript; Transcript Identified by AceView
TC0X00007890.hg.1	4.38	3.71	1.59	0.043	*RPA4*	Replication protein A4
TC1900011990.hg.1	5.73	6.73	-1.99	0.028	*RPL18*	Ribosomal protein L18
TC1200008677.hg.1	7.62	8.88	-2.4	0.027	*RPL18A*	Ribosomal protein L18a
TC1700007740.hg.1	4.19	4.78	-1.5	0.009	*RPL19*	Ribosomal protein L19
TC1700007272.hg.1	6.09	6.94	-1.79	0.021	*RPL21P120*	Ribosomal protein L21 pseudogene 120
TC1800007922.hg.1	5.44	6.22	-1.72	0.040	*RPL21P127*	Ribosomal protein L21 pseudogene 127
TC0800009312.hg.1	6.82	7.55	-1.66	0.031	*RPL23AP53*	Ribosomal protein L23a pseudogene 53
TC1500006663.hg.1	8.5	9.39	-1.86	0.016	*RPL41P2*	Ribosomal protein L41 pseudogene 2

TSUnmapped00000677.hg.1	5.89	6.92	-2.05	0.017	*RPL7A*	Ribosomal protein L7a
TC0100009903.hg.1	3.82	4.44	-1.54	0.025	*RPRD2*	Regulation of nuclear pre-mRNA domain containing 2
TC2000006769.hg.1	5.71	6.7	-1.99	0.022	*RPS10P2*	Ribosomal protein S10 pseudogene 2
TC2000008606.hg.1	4.33	5.22	-1.85	0.036	*RPS15AP1*	Ribosomal protein S15a pseudogene 1
TC1600009077.hg.1	3.7	4.32	-1.54	0.020	*RPS2; SNORA64; SNORA10*	Ribosomal protein S2; small nucleolar RNA, H/ACA box 64; small nucleolar RNA, H/ACA box 10
TC0800010502.hg.1	10.02	12.45	-5.4	0.009	*RPS20; SNORD54*	Ribosomal protein S20; small nucleolar RNA, C/D box 54
TC1300009638.hg.1	8.62	10.99	-5.18	0.044	*RPS26*	Homo sapiens ribosomal protein S26, mRNA (cDNA clone MGC:104291 IMAGE:4287636), complete cds.
TC1200007823.hg.1	7.11	8.22	-2.16	0.039	*RPS26*	Ribosomal protein S26
TC0X00007586.hg.1	12.02	13.41	-2.61	0.033	*RPS26P11*	Ribosomal protein S26 pseudogene 11
TC0400010344.hg.1	5.27	4.68	1.5	0.025	*RPS3AP17*	Ribosomal protein S3a pseudogene 17 [Source:HGNC Symbol;Acc:HGNC:36083]
TSUnmapped00000108.hg.1	3.76	4.36	-1.51	0.019	*RPS6KA1*	Ribosomal protein S6 kinase, 90kDa, polypeptide 1
TC0700008147.hg.1	5.28	6.23	-1.94	4E-04	*RSBN1L*	Round spermatid basic protein 1-like
TC0100012113.hg.1	3.93	3.22	1.64	0.041	*RYR2*	Ryanodine receptor 2 (cardiac)
TC1900008409.hg.1	4.72	5.56	-1.79	0.013	*SAE1*	SUMO1 activating enzyme subunit 1
TC0400009330.hg.1	4.39	4.98	-1.51	0.012	*SAP30*	Sin3A associated protein 30kDa
TC1100010077.hg.1	5.72	6.7	-1.96	0.045	*SBF2*	SET binding factor 2
TC1200010510.hg.1	4.03	5.28	-2.37	0.001	*SCAF11*	SR-related CTD-associated factor 11
TC0200015865.hg.1	3.87	3.26	1.53	0.036	*SCG2*	Secretogranin II
TC0900010151.hg.1	9.39	8.55	1.78	0.049	*SDR42E1P2*	Short chain dehydrogenase/reductase family 42E, member 1 pseudogene 2 [Source:HGNC Symbol;Acc:HGNC:51833]
TC0100013553.hg.1	6.81	7.48	-1.59	0.043	*SEPW1P*	Selenoprotein W, 1 pseudogene
TC0500007943.hg.1	4.88	4.03	1.81	0.006	*SHFM1P1*	Split hand/foot malformation (ectrodactyly) type 1 pseudogene 1 [Source:HGNC Symbol;Acc:HGNC:24454]
TC1000007846.hg.1	4.83	5.48	-1.57	0.015	*SIRT1*	Sirtuin 1
TC0600008422.hg.1	3.17	3.8	-1.55	1E-04	*SLC25A51P1*	Solute carrier family 25, member 51 pseudogene 1
TC2000009267.hg.1	3.46	4.07	-1.52	0.016	*SLC35C2*	Solute carrier family 35 (GDP-fucose transporter), member C2
TC1200011993.hg.1	5.95	6.8	-1.8	0.018	*SLC8B1*	Solute carrier family 8 (sodium/lithium/calcium exchanger), member B1
TC1600011514.hg.1	8.27	10.19	-3.79	0.018	*SMG1P2*	SMG1 pseudogene 2
TC1600011559.hg.1	4.26	5.25	-1.98	0.038	*SMG1P7*	SMG1 pseudogene 7
TC1900008593.hg.1	8.57	10.56	-3.98	0.017	*SNAR-F*	Small ILF3/NF90-associated RNA F
TC0200013161.h	9.07	10.04	-1.96	0.037	*SNAR-H*	Small ILF3/NF90-associated RNA H

g.1						
TC0200010483.hg.1	6.41	7.88	-2.76	0.007	*SNORD11*	Small nucleolar RNA, C/D box 11
TC1400008245.hg.1	4.94	7.72	-6.88	0.027	*SNORD113-1*	Small nucleolar RNA, C/D box 113-1
TC1400010673.hg.1	7.54	6.76	1.72	0.044	*SNORD114-17*	Small nucleolar RNA, C/D box 114-17
TC0200016020.hg.1	4.47	5.25	-1.72	0.001	*SNORD20*	Small nucleolar RNA, C/D box 20
TC0200007112.hg.1	5.87	8.04	-4.49	0.014	*SNORD92*	Small nucleolar RNA, C/D box 92
TC1900008121.hg.1	4.74	5.46	-1.65	0.021	*SNRPA*	Small nuclear ribonucleoprotein polypeptide A
TC1400007221.hg.1	5.37	6.08	-1.64	0.029	*SOCS4*	Suppressor of cytokine signaling 4
TC0500007617.hg.1	4.87	5.98	-2.15	0.043	*SREK1*	Splicing regulatory glutamine/lysine-rich protein 1
TC0100013497.hg.1	7.28	6.69	1.51	0.032	*SRSF4*	Serine/arginine-rich splicing factor 4
TC1200012163.hg.1	5.01	5.69	-1.61	0.032	*SRSF9*	Serine/arginine-rich splicing factor 9
TC1100009524.hg.1	5.35	6.35	-2.01	0.024	*ST14*	Suppression of tumorigenicity 14 (colon carcinoma)
TC0200014623.hg.1	4.29	5.09	-1.74	0.043	*STAM2*	Signal transducing adaptor molecule (SH3 domain and ITAM motif) 2
TC1700007883.hg.1	4.92	4.29	1.55	0.012	*STAT5A*	Signal transducer and activator of transcription 5A
TC2000007476.hg.1	6.6	7.77	-2.26	0.040	*STK4*	Serine/threonine kinase 4
TC2000009317.hg.1	5.83	6.76	-1.91	0.003	*SULF2*	Sulfatase 2
TC0800010745.hg.1	5.24	4.6	1.56	0.020	*SUMO2P20*	SUMO2 pseudogene 20 [Source:HGNC Symbol;Acc:HGNC:49355]
TC0400010132.hg.1	4.01	4.66	-1.57	0.010	*TAPT1*	Transmembrane anterior posterior transformation 1
TC0800011710.hg.1	5.04	5.99	-1.93	0.016	*TATDN1; MIR6844*	TatD DNase domain containing 1; microRNA 6844
TC0300013948.hg.1	7.96	8.84	-1.84	0.049	*TBC1D5*	TBC1 domain family, member 5
TC0700009049.hg.1	7.07	7.66	-1.51	0.003	*TCONS_l2_00026199; TCONS_l2_00026193; TCONS_l2_00026196; TCONS_l2_00026190; TCONS_l2_00026194; TCONS_l2_00026204; TCONS_l2_00026200; RP11-274B21.14; RNU6-177P*	Jeck2013 ALT_DONOR, downstream_end, ncRNA, OVEXON best transcript TCONS_l2_00026199; Jeck2013 ALT_DONOR, downstream_end, ncRNA, OVEXON best transcript TCONS_l2_00026193; Jeck2013 ALT_DONOR, downstream_end, ncRNA, OVEXON best transcript TCONS_l2_00026196; Jeck2013 ALT_DONOR, INTERNAL, ncRNA, OVEXON best transcript TCONS_l2_00026199; Jeck2013 ANNOTATED, INTERNAL, ncRNA, OVEXON best transcript TCONS_l2_00026190; Jeck2013 ANNOTATED, INTERNAL, ncRNA, OVEXON best transcript TCONS_l2_00026194; Jeck2013 ANNOTATED, ncRNA, OVEXON best transcript TCONS_l2_00026204; Jeck2013 ANNOTATED, INTERNAL, ncRNA, OVEXON best transcript TCONS_l2_00026200; novel transcript; RNA, U6 small nuclear 177, pseudogene [Source:HGNC Symbol;Acc:HGNC:47140]

TC0600013762.hg.1	5.64	6.73	-2.13	0.004	*TCP1; SNORA20; SNORA29*	T-complex 1; small nucleolar RNA, H/ACA box 20; small nucleolar RNA, H/ACA box 29
TC1200008669.hg.1	5.82	7.17	-2.54	0.008	*TDG*	Thymine DNA glycosylase
TC1900012000.hg.1	4.76	5.61	-1.81	0.033	*TEAD2*	TEA domain family member 2
TC1800006513.hg.1	6.7	7.3	-1.51	0.046	*TGIF1*	TGFB-induced factor homeobox 1
TC0200008335.hg.1	4.45	5.1	-1.57	0.016	*THNSL2*	Threonine synthase-like 2
TC1000007596.hg.1	6.67	7.51	-1.79	0.007	*TIMM23B*	Translocase of inner mitochondrial membrane 23 homolog B (yeast)
TC0600008092.hg.1	3.73	4.43	-1.63	2E-04	*TJAP1*	Tight junction associated protein 1 (peripheral)
TC0300007335.hg.1	5.38	6.62	-2.36	0.042	*TMA7*	Translation machinery associated 7 homolog
TC0Y00007172.hg.1	6.73	6.07	1.58	0.022	*TMEM167AP1*	Transmembrane protein 167A pseudogene 1 [Source:HGNC Symbol;Acc:HGNC:23956]
TC2000007380.hg.1	5.62	6.22	-1.51	0.020	*TOP1*	Topoisomerase (DNA) I
TC0300012475.hg.1	12.2	13.07	-1.82	0.012	*TOPBP1*	Topoisomerase (DNA) II binding protein 1
TC2000007089.hg.1	3.88	4.64	-1.68	0.010	*TPX2*	TPX2, microtubule-associated
TC1400006638.hg.1	4.72	3.99	1.66	0.007	*TRAJ19*	T cell receptor alpha joining 19 (non-functional)
TC1400006612.hg.1	4.35	3.76	1.5	0.049	*TRAV39*	T cell receptor alpha variable 39
TC0700013538.hg.1	4.08	3.43	1.57	0.036	*TRGJ1; TRGC2; TRGJ2; TRGV9; TRGC1; TRGJP; TRGJP2*	T cell receptor gamma joining 1; T cell receptor gamma constant 2; T cell receptor gamma joining 2; T cell receptor gamma variable 9; T cell receptor gamma constant 1; T cell receptor gamma joining P; T cell receptor gamma joining P2
TC0800008801.hg.1	6.31	6.91	-1.52	0.019	*TRIB1*	Tribbles pseudokinase 1
TC1100007287.hg.1	5.06	6.11	-2.07	0.044	*TRIM44*	Tripartite motif containing 44
TC0200013602.hg.1	3.28	3.92	-1.56	0.015	*TSGA10*	Testis specific 10
TC0500009268.hg.1	4.29	5.1	-1.75	0.023	*TTC1*	Tetratricopeptide repeat domain 1
TC2200007078.hg.1	4.46	5.46	-2	0.002	*TUG1*	Taurine up-regulated 1 (non-protein coding)
TC1800009206.hg.1	4.98	7.4	-5.35	0.024	*TXNL4A*	Thioredoxin-like 4A
TC2000007503.hg.1	3.29	4.87	-2.97	0.007	*UBE2C*	Ubiquitin-conjugating enzyme E2C
TC0300006827.hg.1	5.63	6.4	-1.71	0.046	*UBE2E2*	Ubiquitin conjugating enzyme E2E 2
TC0200010152.hg.1	5.27	6.18	-1.89	0.011	*UBE2E3*	Ubiquitin-conjugating enzyme E2E 3
TC0100015918.hg.1	3.71	4.57	-1.81	0.049	*UBE2Q1*	Ubiquitin-conjugating enzyme E2Q family member 1
TC0200006951.hg.1	3.8	4.44	-1.55	0.012	*UBXN2A*	UBX domain protein 2A
TC0600007771.hg.1	3.42	4.11	-1.62	0.001	*UHRF1BP1*	UHRF1 binding protein 1
TC1000009802.hg.1	3.88	4.5	-1.53	0.035	*UPF2*	UPF2 regulator of nonsense transcripts homolog (yeast)

TC2000008968.hg.1	3.77	4.69	-1.9	0.008	*UQCC1*	Ubiquinol-cytochrome c reductase complex assembly factor 1
TC0200012734.hg.1	7.08	8.14	-2.09	0.035	*USP34*	Ubiquitin specific peptidase 34
TC1600009326.hg.1	4.71	5.46	-1.69	0.022	*USP7*	Ubiquitin specific peptidase 7 (herpes virus-associated)
TC0Y00007141.hg.1	4.63	3.91	1.64	0.003	*USP9YP2*	Ubiquitin specific peptidase 9, Y-linked pseudogene 2 [Source:HGNC Symbol;Acc:HGNC:31742]
TC0Y00006565.hg.1	4.23	3.49	1.67	0.005	*USP9YP22*	Ubiquitin specific peptidase 9, Y-linked pseudogene 22 [Source:HGNC Symbol;Acc:HGNC:38758]
TC1800006656.hg.1	6.39	8.1	-3.29	0.034	*VAPA*	VAMP associated protein A
TC1400010649.hg.1	4.7	5.47	-1.71	0.021	*VRK1*	Vaccinia related kinase 1
TC0200007664.hg.1	4.29	5.31	-2.04	0.045	*VRK2*	Vaccinia related kinase 2
TC0900009339.hg.1	4.2	5.45	-2.39	0.004	*WASH1*	WAS protein family homolog 1
TC1100011107.hg.1	5.02	5.98	-1.95	9.44E-05	*WDR74*	WD repeat domain 74
TC1700007319.hg.1	5.07	6.67	-3.02	0.010	*WSB1*	WD repeat and SOCS box containing 1
TC1200012105.hg.1	5.42	7.31	-3.72	0.015	*WSB2*	WD repeat and SOCS box containing 2
TC0800012316.hg.1	5.07	4.25	1.76	0.025	*XKR9*	X-linked Kx blood group related 9
TC0600011878.hg.1	4.08	4.68	-1.52	0.005	*XPO5*	Exportin 5
TC1200008002.hg.1	5.85	6.74	-1.85	0.002	*XPOT*	Exportin, tRNA
TC2000009800.hg.1	4.48	5.73	-2.38	0.001	*YTHDF1*	YTH N(6)-methyladenosine RNA binding protein 1
TC0100018506.hg.1	4.36	5.03	-1.59	0.011	*YY1AP1*	YY1 associated protein 1
TC0200013899.hg.1	4.13	5.39	-2.39	0.002	*ZC3H8*	Zinc finger CCCH-type containing 8
TC0800012435.hg.1	4.62	5.29	-1.6	0.043	*ZFAND1*	Zinc finger, AN1-type domain 1
TC0600007887.hg.1	5	5.86	-1.82	0.034	*ZFAND3*	Zinc finger, AN1-type domain 3
TC1900008267.hg.1	4.34	3.7	1.55	0.027	*ZNF285B*	Zinc finger protein 285B, pseudogene
TC0600011162.hg.1	4.45	5.13	-1.6	0.010	*ZNF322*	Zinc finger protein 322
TC2000009973.hg.1	4.4	5.15	-1.68	0.036	*ZNF343*	Zinc finger protein 343
TC1900010163.hg.1	8.76	9.64	-1.84	0.028	*ZNF43*	Zinc finger protein 43
TC1900012022.hg.1	5.48	6.42	-1.92	0.049	*ZNF616*	Zinc finger protein 616
TC0300009600.hg.1	3.55	4.31	-1.7	0.012	*ZNF639*	Zinc finger protein 639
TC0100018088.hg.1	7.19	6.49	1.63	0.019	*ZNF669*	Zinc finger protein 669
TC0700011298.hg.1	4.38	6.31	-3.83	0.007	*ZNF680*	Zinc finger protein 680
TC1900011919.hg.1	4.3	5.23	-1.91	0.008	*ZNF708*	Zinc finger protein 708
TC0700007727.hg.1	3.86	4.56	-1.62	0.014	*ZNF734P*	Zinc finger protein 734, pseudogene [Source:HGNC Symbol;Acc:HGNC:32465]

TC1900011793.hg.1	7.47	8.06	-1.51	0.042	*ZNF808; RPL39P34*	Zinc finger protein 808; ribosomal protein L39 pseudogene 34
TC1000010686.hg.1	4.7	5.36	-1.58	0.016	*ZWINT*	ZW10 interacting kinetochore protein
