# Supplementary material for: Identification of Thrombosis-Related Genes in Patients with Advanced Gastric Cancer: Data from AGAMENON-SEOM Registry
Source: Biomedicines. 2022 Jan 11;10(1):148. doi: 10.3390/biomedicines10010148 (PMC8773420; doi:10.3390/biomedicines10010148)
Supplement: Supplementary file 1 [file biomedicines-10-00148-s001.zip › biomedicines-1533227-supplementary/Table S4.pdf]

Table S4.

ID	T Avg (log2)	N Avg (log2)	Fold Change	P-value	Gene Symbol	Description
TC1200010798.hg.1	7.13	5.34	3.45	0.045	*AAAS*	Achalasia, adrenocortical insufficiency, alacrimia
TC1700011731.hg.1	10.42	8.67	3.36	0.049	*ATP5H*	ATP synthase, H+ transporting, mitochondrial Fo complex subunit D
TC1800007797.hg.1	4.44	3.79	1.57	0.014	*ATP9B*	ATPase, class II, type 9B
TC1100009040.hg.1	4.87	4.06	1.75	0.043	*C11orf1*	Chromosome 11 open reading frame 1
TC1200011400.hg.1	4.77	4.06	1.64	0.001	*CCDC59*	Coiled-coil domain containing 59
TC1100010472.hg.1	5.89	5.2	1.61	0.025	*CCDC73*	Coiled-coil domain containing 73
TC0400012620.hg.1	4.3	4.94	-1.55	0.010	*CENPU*	Centromere protein U
TC1200011557.hg.1	4.2	4.98	-1.71	0.007	*CEP83*	Centrosomal protein 83kDa
TC1100007220.hg.1	4.1	5.04	-1.91	0.006	*DEPDC7*	DEP domain containing 7
TC0900006961.hg.1	5.08	4.38	1.62	0.024	*DNAJA1*	DnaJ (Hsp40) homolog, subfamily A, member 1
TC0X00008578.hg.1	4.56	3.87	1.61	0.038	*F9*	Coagulation factor IX
TC0100016841.hg.1	4.54	5.2	-1.58	0.005	*FAM204BP*	Family with sequence similarity 204 member B, pseudogene [Source:HGNC Symbol;Acc:HGNC:42011]
TC1100012029.hg.1	5.39	4.07	2.48	0.012	*FAM76B*	Family with sequence similarity 76, member B
TC0400012452.hg.1	3.7	4.29	-1.51	0.007	*FBXO8*	F-box protein 8
TSUnmapped00000392.hg.1	5.13	5.74	-1.53	0.026	*FCGBP*	Fc fragment of IgG binding protein
TC1900009404.hg.1	4.64	5.59	-1.93	0.004	*FUT6*	Fucosyltransferase 6 (alpha (1,3) fucosyltransferase)
TC0X00008316.hg.1	4.51	5.14	-1.55	9.47E-05	*GRIA3*	Glutamate receptor, ionotropic, AMPA 3
TC0500013321.hg.1	8.79	7.37	2.67	2E-04	*GTF2H2*	General transcription factor IIH subunit 2
TC0500007694.hg.1	9.05	8.35	1.63	3E-04	*GTF2H2; GTF2H2B*	General transcription factor IIH subunit 2; general transcription factor IIH subunit 2B (pseudogene)
TC1600007982.hg.1	5.77	4.97	1.74	0.002	*HERPUD1*	Homocysteine-inducible, endoplasmic reticulum stress-inducible, ubiquitin-like domain member 1
TC0500013219.hg.1	4.04	4.73	-1.61	0.003	*HSD17B4*	Hydroxysteroid (17-beta) dehydrogenase 4
TC0400007772.hg.1	5.87	5.28	1.51	0.016	*HTN1*	Histatin 1
TC0200006648.hg.1	4.35	3.63	1.65	0.009	*IAH1*	Isoamyl acetate-hydrolyzing esterase 1 homolog
TC0500011852.hg.1	4.43	5.05	-1.55	0.049	*LOX*	Lysyl oxidase

TC0600007231.hg.1	5.77	5.15	1.53	0.006	*LRRC16A*	Leucine rich repeat containing 16A
TC0300013334.hg.1	7.63	7.02	1.53	0.029	*MCCC1*	Methylcrotonoyl-CoA carboxylase 1
TC0X00006671.hg.1	4.56	3.96	1.52	0.031	*MOSPD2*	Motile sperm domain containing 2
TC1600011007.hg.1	5.25	4.56	1.61	0.002	*MPHOSPH6*	M-phase phosphoprotein 6
TC0600007610.hg.1	5.21	6.6	-2.62	0.038	*MSH5; MSH5-SAPCD1; SAPCD1*	MutS homolog 5; MSH5-SAPCD1 readthrough (NMD candidate); suppressor APC domain containing 1
TC0200016582.hg.1	5.94	5.25	1.61	0.025	*NABP1*	Nucleic acid binding protein 1
TC0100007886.hg.1	6.74	5.93	1.75	0.036	*NDUFS5*	NADH dehydrogenase (ubiquinone) Fe-S protein 5, 15kDa (NADH-coenzyme Q reductase)
TC1000011704.hg.1	5.24	3.76	2.78	0.002	*NT5C2*	5-nucleotidase, cytosolic II
TC0400009258.hg.1	7.09	6.36	1.65	0.014	*PALLD*	Palladin, cytoskeletal associated protein
TC2000007792.hg.1	4.73	4.02	1.63	0.037	*PFDN4*	Prefoldin subunit 4
TC0500008540.hg.1	4.32	3.73	1.5	0.001	*PHAX*	Phosphorylated adaptor for RNA export
TC0100017113.hg.1	7.77	11.41	-12.49	0.024	*PIGR*	Polymeric immunoglobulin receptor
TC1000010022.hg.1	6.36	5.52	1.79	0.038	*PIP4K2A*	Phosphatidylinositol-5-phosphate 4-kinase, type II, alpha
TC0200008281.hg.1	6.74	6.07	1.59	0.034	*PTCD3*	Pentatricopeptide repeat domain 3
TC1800006889.hg.1	5.31	3.79	2.87	0.040	*RIOK3*	RIO kinase 3
TC0100017230.hg.1	5.87	4.92	1.94	0.045	*RPL21P28*	Ribosomal protein L21 pseudogene 28
TC0X00011310.hg.1	6.21	5.42	1.73	0.032	*RPL36A-HNRNPH2*	RPL36A-HNRNPH2 readthrough
TC2000009194.hg.1	7.12	6.43	1.61	0.049	*RPL37AP1*	Ribosomal protein L37a pseudogene 1
TC1400009134.hg.1	5.27	6.28	-2	0.029	*SAV1*	Salvador family WW domain containing protein 1
TC0800007738.hg.1	5.97	5.2	1.71	0.019	*SDCBP*	Syndecan binding protein
TC1700008705.hg.1	12.49	11.29	2.29	0.049	*SNORA38B*	Small nucleolar RNA, H/ACA box 38B
TC0200009487.hg.1	4.64	5.31	-1.6	0.032	*SPOPL*	Speckle-type POZ protein-like
TC0X00010242.hg.1	4.93	5.74	-1.75	0.028	*SRIP2*	Sorcin pseudogene 2 [Source:HGNC Symbol;Acc:HGNC:38734]
TC0X00008332.hg.1	5.6	6.65	-2.06	0.045	*STAG2*	Stromal antigen 2
TC1700010497.hg.1	4.76	4.08	1.6	0.035	*SYNRG*	Synergin, gamma
TC0400007876.hg.1	4.22	3.54	1.6	0.046	*THAP6*	THAP domain containing 6
TC0600008262.hg.1	3.74	4.37	-1.55	1E-04	*TMEM14A*	Transmembrane protein 14A
TC0100017815.hg.1	7.06	6.3	1.7	3E-04	*TOMM20*	Translocase of outer mitochondrial membrane 20 homolog (yeast)
TC0100015899.hg.1	8.89	8.15	1.67	0.028	*TPM3*	Tropomyosin 3

TC0700010472.hg.1	6.26	5.26	2	0.004	*TRA2A*	Transformer 2 alpha homolog (Drosophila)
TC0300009625.hg.1	5.97	5.02	1.93	0.017	*TTC14*	Tetratricopeptide repeat domain 14
TC0X00008253.hg.1	7.01	4.85	4.46	0.011	*UBE2A*	Ubiquitin conjugating enzyme E2A
TC0800011171.hg.1	6.29	5.37	1.9	0.037	*UQCRB*	Ubiquinol-cytochrome c reductase binding protein
TC1900007695.hg.1	4.87	4	1.83	0.010	*URI1*	URI1, prefoldin-like chaperone
TC0100007570.hg.1	5.97	5.34	1.55	0.027	*YTHDF2*	YTH N(6)-methyladenosine RNA binding protein 2
TC1900011935.hg.1	4.98	4.38	1.51	0.009	*ZFP30*	ZFP30 zinc finger protein
TC1900010886.hg.1	5.21	4.6	1.53	0.004	*ZNF235*	Zinc finger protein 235
TC1900011791.hg.1	4.68	3.73	1.93	0.025	*ZNF613*	Zinc finger protein 613
TSUnmapped00000369.hg.1	5.43	4.77	1.58	0.001	*ZNF780B*	Zinc finger protein 780B
TC0700013615.hg.1	4.44	5.23	-1.73	0.023	*ZNF800*	Zinc finger protein 800
