# Supplementary material for: Identification of Thrombosis-Related Genes in Patients with Advanced Gastric Cancer: Data from AGAMENON-SEOM Registry
Source: Biomedicines. 2022 Jan 11;10(1):148. doi: 10.3390/biomedicines10010148 (PMC8773420; doi:10.3390/biomedicines10010148)
Supplement: Supplementary file 1 [file biomedicines-10-00148-s001.zip › biomedicines-1533227-supplementary/Table S5.pdf]

**Table S5.**

| ID | T Avg (log2) | N Avg (log2) | Fold Change | P-value | Gene Symbol | Description |
|---|---|---|---|---|---|---|
| TC1700006729.hg.1 | 6.36 | 5.56 | 1.74 | 0.029 | *ACADVL* | Acyl-CoA dehydrogenase, very long chain |
| TC2000009440.hg.1 | 5.17 | 4.56 | 1.53 | 0.020 | *ADNP* | Activity-dependent neuroprotector homeobox |
| TC0100018006.hg.1 | 6.53 | 5.65 | 1.84 | 0.028 | *ADSS* | Adenylosuccinate synthase |
| TC0700010355.hg.1 | 13.99 | 9.87 | 17.36 | 0.011 | *AGR2* | Anterior gradient 2, protein disulphide isomerase family member |
| TC1200011770.hg.1 | 4.58 | 5.24 | -1.58 | 0.037 | *ALDH1L2* | Aldehyde dehydrogenase 1 family, member L2 |
| TC1200012707.hg.1 | 5.62 | 4.81 | 1.75 | 0.044 | *ALDH2* | Aldehyde dehydrogenase 2 family (mitochondrial) |
| TC0500009555.hg.1 | 5.07 | 5.78 | -1.63 | 0.022 | *ARL2BPP6* | ADP ribosylation factor like GTPase 2 binding protein pseudogene 6 [Source:HGNC Symbol;Acc:HGNC:39450] |
| TC0X00007951.hg.1 | 5.72 | 4.98 | 1.67 | 0.006 | *ARMCX3* | Armadillo repeat containing, X-linked 3 |
| TC0600009868.hg.1 | 6.84 | 5.94 | 1.87 | 0.042 | *ARMT1* | Acidic residue methyltransferase 1 |
| TC0100015756.hg.1 | 8.65 | 9.47 | -1.76 | 0.046 | *ARNT* | Aryl hydrocarbon receptor nuclear translocator |
| TC2000007117.hg.1 | 5.43 | 4 | 2.69 | 0.008 | *ASXL1* | Additional sex combs like transcriptional regulator 1 |
| TC1900010105.hg.1 | 6.19 | 6.8 | -1.52 | 0.019 | *BNIP3P19* | BCL2/adenovirus E1B 19kDa interacting protein 3 pseudogene 19 [Source:HGNC Symbol;Acc:HGNC:49699] |
| TC0600013306.hg.1 | 3.79 | 4.44 | -1.57 | 0.011 | *BTF3L4P3* | Basic transcription factor 3-like 4 pseudogene 3 [Source:HGNC Symbol;Acc:HGNC:39647] |
| TC1100012418.hg.1 | 4.44 | 5.04 | -1.52 | 0.009 | *BUD13* | BUD13 homolog |
| TC1400007155.hg.1 | 3.95 | 3.36 | 1.51 | 0.031 | *C14orf166* | Chromosome 14 open reading frame 166 |
| TC0600009406.hg.1 | 4.99 | 4.31 | 1.6 | 0.013 | *C6orf58* | Chromosome 6 open reading frame 58 |
| TC0900007547.hg.1 | 4.1 | 3.46 | 1.56 | 0.041 | *C9orf85* | Chromosome 9 open reading frame 85 |
| TC1200010812.hg.1 | 7.35 | 6.72 | 1.55 | 0.022 | *CALCOCO1* | Calcium binding and coiled-coil domain 1 |
| TC0300006702.hg.1 | 5.57 | 4.88 | 1.61 | 0.002 | *CAPN7* | Calpain 7 |
| TC2000009544.hg.1 | 4.67 | 5.31 | -1.56 | 0.045 | *CBLN4* | Cerebellin 4 precursor |
| TC1000012485.hg.1 | 3.27 | 3.93 | -1.59 | 0.033 | *CC2D2B* | Coiled-coil and C2 domain containing 2B |
| TC0200014004.hg.1 | 4.95 | 3.8 | 2.22 | 0.025 | *CCDC93* | Coiled-coil domain containing 93 |
| TC0600009112.hg.1 | 4.67 | 4 | 1.59 | 0.017 | *CDC40* | Cell division cycle 40 |
| TC1900006524.hg.1 | 4.67 | 5.31 | -1.56 | 0.026 | *CIRBP* | Cold inducible RNA binding protein |
| TC0200007115.hg.1 | 3.48 | 4.36 | -1.84 | 0.014 | *CLIP4* | CAP-GLY domain containing linker protein family, member 4 |
| TC2200006623.hg.1 | 6.39 | 5.75 | 1.56 | 0.033 | *COMT; MIR4761* | Catechol-O-methyltransferase; microRNA 4761 |
| TC0300007389.hg.1 | 3.92 | 4.76 | -1.79 | 0.031 | *COX6CP14* | Cytochrome c oxidase subunit VIc pseudogene 14 [Source:HGNC Symbol;Acc:HGNC:49366] |

| | | | | | | |
|---|---|---|---|---|---|---|
| TC1800008822.hg.1 | 4.63 | 5.27 | -1.55 | 0.004 | *CPLX4* | Complexin 4 |
| TC1700012270.hg.1 | 3.52 | 4.11 | -1.51 | 0.026 | *CRHR1-IT1* | CRHR1 intronic transcript 1 |
| TC0100015353.hg.1 | 7.83 | 6.73 | 2.15 | 0.049 | *CSDE1* | Cold shock domain containing E1, RNA binding |
| TC1100006840.hg.1 | 6.32 | 5.38 | 1.92 | 0.023 | *CTR9* | CTR9 homolog, Paf1/RNA polymerase II complex component |
| TC0600011857.hg.1 | 4.8 | 4.2 | 1.52 | 0.029 | *CUL7* | Cullin 7 |
| TC1700009565.hg.1 | 4.74 | 4 | 1.67 | 0.007 | *DERL2* | Derlin 2 |
| TC0700013392.hg.1 | 5.96 | 5.3 | 1.57 | 0.034 | *DTX2P1-UPK3BP1-PMS2P11* | DTX2P1-UPK3BP1-PMS2P11 readthrough transcribed pseudogene |
| TC1700010798.hg.1 | 3.94 | 3.31 | 1.54 | 0.032 | *DUSP3* | Dual specificity phosphatase 3 |
| TC0200007401.hg.1 | 7.07 | 5.85 | 2.33 | 0.008 | *DYNC2LI1* | Dynein, cytoplasmic 2, light intermediate chain 1 |
| TC0700010444.hg.1 | 17.37 | 15.68 | 3.24 | 0.044 | *EEF1A1P6* | Eukaryotic translation elongation factor 1 alpha 1 pseudogene 6 [Source:HGNC Symbol;Acc:HGNC:3201] |
| TC0400012956.hg.1 | 4.61 | 3.63 | 1.97 | 0.023 | *ELOVL6* | ELOVL fatty acid elongase 6 |
| TC2100006925.hg.1 | 4.59 | 5.18 | -1.51 | 0.047 | *EXOSC3P1* | Exosome component 3 pseudogene 1 [Source:HGNC Symbol;Acc:HGNC:33989] |
| TC1100009445.hg.1 | 6.61 | 5.95 | 1.58 | 0.025 | *FAM118B* | Family with sequence similarity 118, member B |
| TC1300007152.hg.1 | 5.71 | 4.81 | 1.87 | 0.018 | *FNDC3A* | Fibronectin type III domain containing 3A |
| TC0900010236.hg.1 | 9.56 | 8.56 | 2 | 0.021 | *FRG1JP* | FSHD region gene 1 family member J, pseudogene |
| TC0900008314.hg.1 | 3.55 | 4.22 | -1.59 | 0.023 | *FSD1L* | Fibronectin type III and SPRY domain containing 1-like |
| TC2000007915.hg.1 | 11.85 | 8.96 | 7.41 | 0.046 | *GNAS* | GNAS complex locus |
| TC0600011173.hg.1 | 10.36 | 9.14 | 2.33 | 0.038 | *GUSBP2* | Glucuronidase, beta pseudogene 2 |
| TC0500013319.hg.1 | 6.16 | 5.14 | 2.02 | 0.040 | *GUSBP9* | Glucuronidase, beta pseudogene 9 |
| TC1900011113.hg.1 | 5.59 | 4.92 | 1.6 | 0.011 | *GYS1* | Glycogen synthase 1 (muscle) |
| TC0500012282.hg.1 | 3.43 | 4.09 | -1.59 | 0.010 | *HDAC3* | Histone deacetylase 3 |
| TC0200016347.hg.1 | 12.45 | 10.52 | 3.81 | 0.021 | *HDLBP* | High density lipoprotein binding protein |
| TC0700011799.hg.1 | 3.76 | 4.6 | -1.78 | 0.006 | *HEPACAM2* | HEPACAM family member 2 |
| TC0600007290.hg.1 | 4.87 | 4.22 | 1.57 | 0.038 | *HIST1H2BH* | Histone cluster 1, H2bh |
| TC0600011139.hg.1 | 3.28 | 4.01 | -1.66 | 4E-04 | *HIST1H4G* | Histone cluster 1, H4g |
| TC0500006636.hg.1 | 3.83 | 4.5 | -1.59 | 0.003 | *HMGB3P3* | High mobility group box 3 pseudogene 3 [Source:HGNC Symbol;Acc:HGNC:39102] |
| TC1300007250.hg.1 | 8.25 | 7.6 | 1.57 | 0.035 | *HNRNPA1L2* | Heterogeneous nuclear ribonucleoprotein A1-like 2 |
| TC0200015578.hg.1 | 7.59 | 6.55 | 2.07 | 0.047 | *IDH1* | Isocitrate dehydrogenase 1 (NADP+) |
| TC1400010444.hg.1 | 10.64 | 8.46 | 4.53 | 0.046 | *IGHA1* | Immunoglobulin heavy constant alpha 1 |
| TC1400010798.hg.1 | 6.37 | 5.19 | 2.27 | 0.034 | *IGHA2* | Immunoglobulin heavy constant alpha 2 (A2m marker) |

| | | | | | | |
|---|---|---|---|---|---|---|
| TC1400010521.hg.1 | 5.2 | 4.59 | 1.53 | 0.009 | *IGHV3-47* | Immunoglobulin heavy variable 3-47 (pseudogene) |
| TSUnmapped00000647.hg.1 | 5.13 | 4.5 | 1.55 | 0.013 | *IGKV1-17* | Immunoglobulin kappa variable 1-17 [Source:HGNC Symbol;Acc:HGNC:5733] |
| TSUnmapped00000553.hg.1 | 4.69 | 4.03 | 1.59 | 0.004 | *IGKV1-27* | Immunoglobulin kappa variable 1-27 [Source:HGNC Symbol;Acc:HGNC:5735] |
| TC0200013397.hg.1 | 4.54 | 3.87 | 1.6 | 0.013 | *IGKV1-27* | Immunoglobulin kappa variable 1-27 |
| TSUnmapped00000816.hg.1 | 5.32 | 4.44 | 1.84 | 0.002 | *IGKV1-33* | Immunoglobulin kappa variable 1-33 [Source:HGNC Symbol;Acc:HGNC:5737] |
| TSUnmapped00000621.hg.1 | 4.7 | 3.96 | 1.68 | 0.010 | *IGKV1-6* | Immunoglobulin kappa variable 1-6 [Source:HGNC Symbol;Acc:HGNC:5742] |
| TSUnmapped00000665.hg.1 | 6.98 | 5.59 | 2.62 | 0.034 | *IGKV3-20* | Immunoglobulin kappa variable 3-20 [Source:HGNC Symbol;Acc:HGNC:5817] |
| TC2200009222.hg.1 | 9.73 | 7.75 | 3.94 | 0.020 | *IGLC3* | Immunoglobulin lambda constant 3 (Kern-Oz+ marker) |
| TC2200006821.hg.1 | 6.27 | 5.59 | 1.6 | 0.036 | *IGLC6* | Immunoglobulin lambda constant 6 (Kern+Oz- marker, gene/pseudogene) |
| TC2200009219.hg.1 | 7.62 | 6.09 | 2.89 | 0.041 | *IGLL5; MIR5571; IGLC1; IGLC2; IGLV2-5; IGLV3-1; IGLJ1* | Immunoglobulin lambda-like polypeptide 5; microRNA 5571; immunoglobulin lambda constant 1 (Mcg marker); immunoglobulin lambda constant 2 (Kern-Oz- marker); immunoglobulin lambda variable 2-5 (pseudogene); immunoglobulin lambda variable 3-1; immunoglobulin lambda joining 1 |
| TC2200009214.hg.1 | 4.74 | 4.07 | 1.59 | 0.019 | *IGLV2-18* | Immunoglobulin lambda variable 2-18 |
| TC0400010961.hg.1 | 8.16 | 6.01 | 4.44 | 0.038 | *JCHAIN* | Joining chain of multimeric IgA and IgM |
| TC1600008971.hg.1 | 7.34 | 6.6 | 1.67 | 0.046 | *JMJD8* | Jumonji domain containing 8 |
| TC0600009426.hg.1 | 3.81 | 4.54 | -1.66 | 0.023 | *LAMA2* | Laminin, alpha 2 |
| TC1900010625.hg.1 | 8.02 | 5.93 | 4.25 | 0.027 | *LGALS4* | Lectin, galactoside-binding, soluble, 4 |
| TC0900007439.hg.1 | 4 | 4.64 | -1.56 | 0.008 | *LOC101928381; yasero* | Homo sapiens uncharacterized LOC101928381 (LOC101928381), long non-coding RNA.; Transcript Identified by AceView |
| TC0900007768.hg.1 | 4.34 | 5 | -1.58 | 0.028 | *LOC389765* | Kinesin family member 27 pseudogene |
| TC0X00007092.hg.1 | 4.24 | 5.01 | -1.71 | 0.001 | *LOC392452* | Mitochondrial fission factor pseudogene; Transcript Identified by AceView, Entrez Gene ID(s) 392452 |
| TC2200009352.hg.1 | 5.94 | 5.04 | 1.86 | 0.042 | *LOC400927; CSNK1E* | TPTE and PTEN homologous inositol lipid phosphatase pseudogene; Transcript Identified by AceView, Entrez Gene ID(s) 1454; 400927 |
| TC0100014605.hg.1 | 3.72 | 4.44 | -1.65 | 0.007 | *LRRIQ3* | Leucine-rich repeats and IQ motif containing 3 |
| TC0100017137.hg.1 | 4.78 | 4.16 | 1.55 | 0.005 | *MIR29B2; MIR29C* | MicroRNA 29b-2; microRNA 29c |
| TC0400009143.hg.1 | 3.51 | 4.13 | -1.54 | 0.046 | *MIR3688-2* | MicroRNA 3688-2 |
| TC1900008807.hg.1 | 3.88 | 4.54 | -1.58 | 0.030 | *MIR516A1* | MicroRNA 516a-1 |
| TC1300006474.hg.1 | 5.42 | 4.78 | 1.56 | 0.001 | *MPHOSPH8* | M-phase phosphoprotein 8 |
| TC0200012072.hg.1 | 7 | 6.36 | 1.56 | 0.034 | *MPV17* | MpV17 mitochondrial inner membrane protein |

| | | | | | | |
|---|---|---|---|---|---|---|
| TC2000007751.hg.1 | 6.76 | 7.38 | -1.54 | 0.001 | *MRPS33P4* | Mitochondrial ribosomal protein S33 pseudogene 4 [Source:HGNC Symbol;Acc:HGNC:29767] |
| TC0200008326.hg.1 | 8.06 | 7.09 | 1.96 | 0.012 | *MTATP8P2* | Mitochondrially encoded ATP synthase 8 pseudogene 2 [Source:HGNC Symbol;Acc:HGNC:44572] |
| TC1800008620.hg.1 | 7.36 | 6.51 | 1.8 | 0.036 | *MTCO2P2* | MT-CO2 pseudogene 2 [Source:HGNC Symbol;Acc:HGNC:25354] |
| TC1000010266.hg.1 | 3.8 | 4.47 | -1.58 | 0.008 | *MTND4LP11* | Mitochondrially encoded NADH:ubiquinone oxidoreductase core subunit 4L pseudogene 11 [Source:HGNC Symbol;Acc:HGNC:42245] |
| TC0100012126.hg.1 | 5.39 | 6.03 | -1.56 | 0.019 | *MTND6P15* | Mitochondrially encoded NADH:ubiquinone oxidoreductase core subunit 6 pseudogene 15 [Source:HGNC Symbol;Acc:HGNC:39478] |
| TC0400007682.hg.1 | 9.36 | 10.21 | -1.81 | 0.004 | *MTND6P16* | Mitochondrially encoded NADH:ubiquinone oxidoreductase core subunit 6 pseudogene 16 [Source:HGNC Symbol;Acc:HGNC:39479] |
| TC1600006884.hg.1 | 5.46 | 6.18 | -1.65 | 0.006 | *MTND6P33* | Mitochondrially encoded NADH:ubiquinone oxidoreductase core subunit 6 pseudogene 33 [Source:HGNC Symbol;Acc:HGNC:52175] |
| TC1700007296.hg.1 | 8.63 | 7.45 | 2.27 | 0.031 | *MTRNR2L1* | MT-RNR2-like 1 |
| TC0500011282.hg.1 | 10.66 | 8.83 | 3.54 | 0.027 | *MTRNR2L2* | MT-RNR2-like 2 |
| TC1100010087.hg.1 | 10.17 | 8.36 | 3.51 | 0.036 | *MTRNR2L8; MIR4485* | MT-RNR2-like 8; microRNA 4485 |
| TC1300010030.hg.1 | 13.17 | 10 | 8.98 | 0.048 | *N4BP2L2* | NEDD4 binding protein 2-like 2 |
| TC0400011687.hg.1 | 5.13 | 5.81 | -1.6 | 0.010 | *NDUFS5P5* | NADH:ubiquinone oxidoreductase subunit S5 pseudogene 5 [Source:HGNC Symbol;Acc:HGNC:44043] |
| TC1800009236.hg.1 | 5.52 | 6.15 | -1.55 | 0.003 | *OACYLP* | O-acyltransferase like, pseudogene [Source:HGNC Symbol;Acc:HGNC:44362] |
| TC1100007760.hg.1 | 4.29 | 4.91 | -1.54 | 0.045 | *OOSP1* | Oocyte secreted protein 1, pseudogene |
| TC0900008295.hg.1 | 3.87 | 4.49 | -1.54 | 0.004 | *OR13D1* | Olfactory receptor, family 13, subfamily D, member 1 |
| TC0100018124.hg.1 | 4.46 | 5.34 | -1.84 | 0.034 | *OR2M7* | Olfactory receptor, family 2, subfamily M, member 7 |
| TC1100013141.hg.1 | 4.15 | 4.8 | -1.57 | 0.007 | *OVCH2* | Ovochymase 2 (gene/pseudogene) |
| TC0300013804.hg.1 | 5.81 | 5.12 | 1.61 | 0.015 | *OXSM* | 3-oxoacyl-ACP synthase, mitochondrial |
| TC0300007004.hg.1 | 4.08 | 4.84 | -1.7 | 0.011 | *PDCD6IP* | Programmed cell death 6 interacting protein |
| TC1100012303.hg.1 | 3.86 | 4.5 | -1.56 | 0.031 | *PPP2R1B* | Protein phosphatase 2, regulatory subunit A, beta |
| TC1400010596.hg.1 | 4.58 | 3.96 | 1.54 | 0.009 | *PSMA6* | Proteasome subunit alpha 6 |
| TSUnmapped00000597.hg.1 | 8.87 | 7.75 | 2.17 | 0.045 | *PSMC4* | Proteasome 26S subunit, ATPase 4 [Source:HGNC Symbol;Acc:HGNC:9551] |
| TC0700008141.hg.1 | 5.3 | 3.46 | 3.6 | 0.014 | *PTPN12* | Protein tyrosine phosphatase, non-receptor type 12 |
| TC0200007205.hg.1 | 7.26 | 7.93 | -1.59 | 0.023 | *RASGRP3* | RAS guanyl releasing protein 3 (calcium and DAG-regulated) |
| TC0300013828.hg.1 | 10.05 | 8.36 | 3.22 | 0.048 | *RBM6* | RNA binding motif protein 6 |

| | | | | | | |
|---|---|---|---|---|---|---|
| TC0100010306.hg.1 | 3.68 | 4.26 | -1.5 | 0.015 | *RP11-404F10.2; skokawbo; teegee* | Novel transcript, antisense to CD48; Transcript Identified by AceView |
| TC1600008386.hg.1 | 3.75 | 4.36 | -1.52 | 0.022 | *RP11-44L9.2; waraw* | Novel transcript; Transcript Identified by AceView |
| TC1000011943.hg.1 | 4.21 | 4.95 | -1.67 | 0.004 | *RPL12P26* | Ribosomal protein L12 pseudogene 26 [Source:HGNC Symbol;Acc:HGNC:36811] |
| TC0800007315.hg.1 | 4.9 | 5.5 | -1.52 | 0.034 | *RPL12P48* | Ribosomal protein L12 pseudogene 48 [Source:HGNC Symbol;Acc:HGNC:51940] |
| TC1900011780.hg.1 | 8.14 | 6.67 | 2.77 | 0.027 | *RPL13A; SNORD35A; SNORD34; SNORD33; SNORD32A* | Ribosomal protein L13a; small nucleolar RNA, C/D box 35A; small nucleolar RNA, C/D box 34; small nucleolar RNA, C/D box 33; small nucleolar RNA, C/D box 32A |
| TC0100008954.hg.1 | 6.22 | 5.51 | 1.64 | 0.023 | *RPL36AP10* | Ribosomal protein L36a pseudogene 10 [Source:HGNC Symbol;Acc:HGNC:36175] |
| TC1200006986.hg.1 | 3.8 | 4.52 | -1.65 | 0.001 | *RPL7P40* | Ribosomal protein L7 pseudogene 40 [Source:HGNC Symbol;Acc:HGNC:36031] |
| TC1200008112.hg.1 | 4.13 | 4.83 | -1.63 | 0.005 | *RPL7P42* | Ribosomal protein L7 pseudogene 42 [Source:HGNC Symbol;Acc:HGNC:35543] |
| TC0600009488.hg.1 | 11.35 | 9.27 | 4.23 | 0.022 | *RPS12* | Ribosomal protein S12 |
| TC0600007693.hg.1 | 10.08 | 9.36 | 1.65 | 0.039 | *RPS18* | Ribosomal protein S18 |
| TC0200006536.hg.1 | 6.2 | 5.3 | 1.86 | 0.037 | *RPS7* | Ribosomal protein S7 |
| TC1900006764.hg.1 | 4.73 | 3.95 | 1.72 | 0.043 | *SAFB* | Scaffold attachment factor B |
| TC1400009002.hg.1 | 5.85 | 4.55 | 2.48 | 0.015 | *SEC23A* | Sec23 homolog A, COPII coat complex component |
| TC1500010729.hg.1 | 5.5 | 4.73 | 1.71 | 0.032 | *SERF2* | Small EDRK-rich factor 2 |
| TC0300014076.hg.1 | 6.5 | 7.23 | -1.67 | 0.028 | *SERPINI2* | Serpin peptidase inhibitor, clade I (pancpin), member 2 |
| TC0100009195.hg.1 | 4.63 | 3.68 | 1.93 | 0.003 | *SLC35A3* | Solute carrier family 35 (UDP-N-acetylglucosamine (UDP-GlcNAc) transporter), member A3 |
| TC0800010737.hg.1 | 3.82 | 4.47 | -1.57 | 0.001 | *SLCO5A1* | Solute carrier organic anion transporter family, member 5A1 |
| TC1700012286.hg.1 | 3.55 | 4.17 | -1.54 | 0.040 | *SMG8* | SMG8 nonsense mediated mRNA decay factor |
| TC1000011804.hg.1 | 6.09 | 6.77 | -1.61 | 0.005 | *SNRPGP12* | Small nuclear ribonucleoprotein polypeptide G pseudogene 12 [Source:HGNC Symbol;Acc:HGNC:39331] |
| TC1000009833.hg.1 | 5.43 | 4.77 | 1.58 | 0.037 | *SNRPGP5* | Small nuclear ribonucleoprotein polypeptide G pseudogene 5 [Source:HGNC Symbol;Acc:HGNC:39324] |
| TC0600013119.hg.1 | 4.78 | 5.45 | -1.58 | 0.015 | *SOGA3; KIAA0408* | SOGA family member 3; KIAA0408 |
| TC0800008371.hg.1 | 3.7 | 4.29 | -1.5 | 0.029 | *SPAG1* | Sperm associated antigen 1 |
| TC1200007561.hg.1 | 5.22 | 4.22 | 2.01 | 0.022 | *SPATS2* | Spermatogenesis associated, serine-rich 2 |
| TC1600006633.hg.1 | 12.13 | 9.79 | 5.07 | 0.040 | *SRRM2* | Serine/arginine repetitive matrix 2 |
| TC0100010537.hg.1 | 7.67 | 6.96 | 1.63 | 0.007 | *SUMO1P2* | SUMO1 pseudogene 2 [Source:HGNC Symbol;Acc:HGNC:33149] |
| TC0600009478.hg.1 | 4.4 | 5.13 | -1.66 | 0.027 | *TAAR7P* | Trace amine associated receptor 7, pseudogene |
| TC0300010274.hg.1 | 3.96 | 4.65 | -1.61 | 0.003 | *TAMM41* | TAM41 mitochondrial translocator assembly and maintenance homolog |

| | | | | | | |
|---|---|---|---|---|---|---|
| TC1200009927.hg.1 | 3.73 | 4.55 | -1.77 | 0.017 | *TAS2R20* | Taste receptor, type 2, member 20 |
| TC1400010777.hg.1 | 7.23 | 6.48 | 1.69 | 0.043 | *TC2N* | Tandem C2 domains, nuclear |
| TC0800010823.hg.1 | 6.49 | 5.39 | 2.15 | 0.040 | *TCEB1* | Transcription elongation factor B (SIII), polypeptide 1 (15kDa, elongin C) |
| TC1400009407.hg.1 | 3.68 | 4.29 | -1.52 | 0.027 | *TEX21P* | Testis expressed 21, pseudogene |
| TC0700013609.hg.1 | 4.28 | 5 | -1.65 | 0.016 | *TFEC* | Transcription factor EC |
| TC1200009247.hg.1 | 5.81 | 4.6 | 2.31 | 0.019 | *TMED2* | Transmembrane p24 trafficking protein 2 |
| TC0700012293.hg.1 | 6.45 | 5.2 | 2.38 | 0.002 | *TMEM168* | Transmembrane protein 168 |
| TC1100011048.hg.1 | 7.42 | 6.06 | 2.56 | 0.029 | *TMEM258; MIR611* | Transmembrane protein 258; microRNA 611 |
| TC1700011208.hg.1 | 7.49 | 6.8 | 1.62 | 0.046 | *TRIM25; MIR3614* | Tripartite motif containing 25; microRNA 3614 |
| TC1900006819.hg.1 | 6.17 | 5.56 | 1.52 | 0.024 | *TRIP10* | Thyroid hormone receptor interactor 10 |
| TC0100008305.hg.1 | 3.53 | 4.15 | -1.53 | 2E-04 | *TXNDC12-AS1* | TXNDC12 antisense RNA 1 |
| TC0300010672.hg.1 | 4.37 | 5.03 | -1.57 | 0.003 | *UBP1* | Upstream binding protein 1 (LBP-1a) |
| TC2200007035.hg.1 | 7.1 | 5.34 | 3.38 | 0.048 | *UQCR10* | Ubiquinol-cytochrome c reductase, complex III subunit X |
| TC0800012410.hg.1 | 9.94 | 6.52 | 10.65 | 0.025 | *WHSC1L1* | Wolf-Hirschhorn syndrome candidate 1-like 1 |
| TC1900009592.hg.1 | 5.35 | 4.71 | 1.56 | 0.009 | *ZNF562* | Zinc finger protein 562 |
| TSUnmapped00000222.hg.1 | 3.6 | 4.22 | -1.54 | 0.015 | *ZNF780A* | Zinc finger protein 780A |
| TSUnmapped00000311.hg.1 | 3.68 | 4.29 | -1.52 | 0.014 | *ZNF780A* | Zinc finger protein 780A |
| TC1900010431.hg.1 | 8.93 | 9.6 | -1.59 | 0.015 | *ZNF807* | Zinc finger protein 807 |
| TC0700011239.hg.1 | 3.38 | 3.99 | -1.53 | 0.001 | *ZNF90P3* | Zinc finger protein 90 pseudogene 3 [Source:HGNC Symbol;Acc:HGNC:39882] |
