# Supplementary figures and images for: Identification of Thrombosis-Related Genes in Patients with Advanced Gastric Cancer: Data from AGAMENON-SEOM Registry
Source: Biomedicines. 2022 Jan 11;10(1):148. doi: 10.3390/biomedicines10010148 (PMC8773420; doi:10.3390/biomedicines10010148)

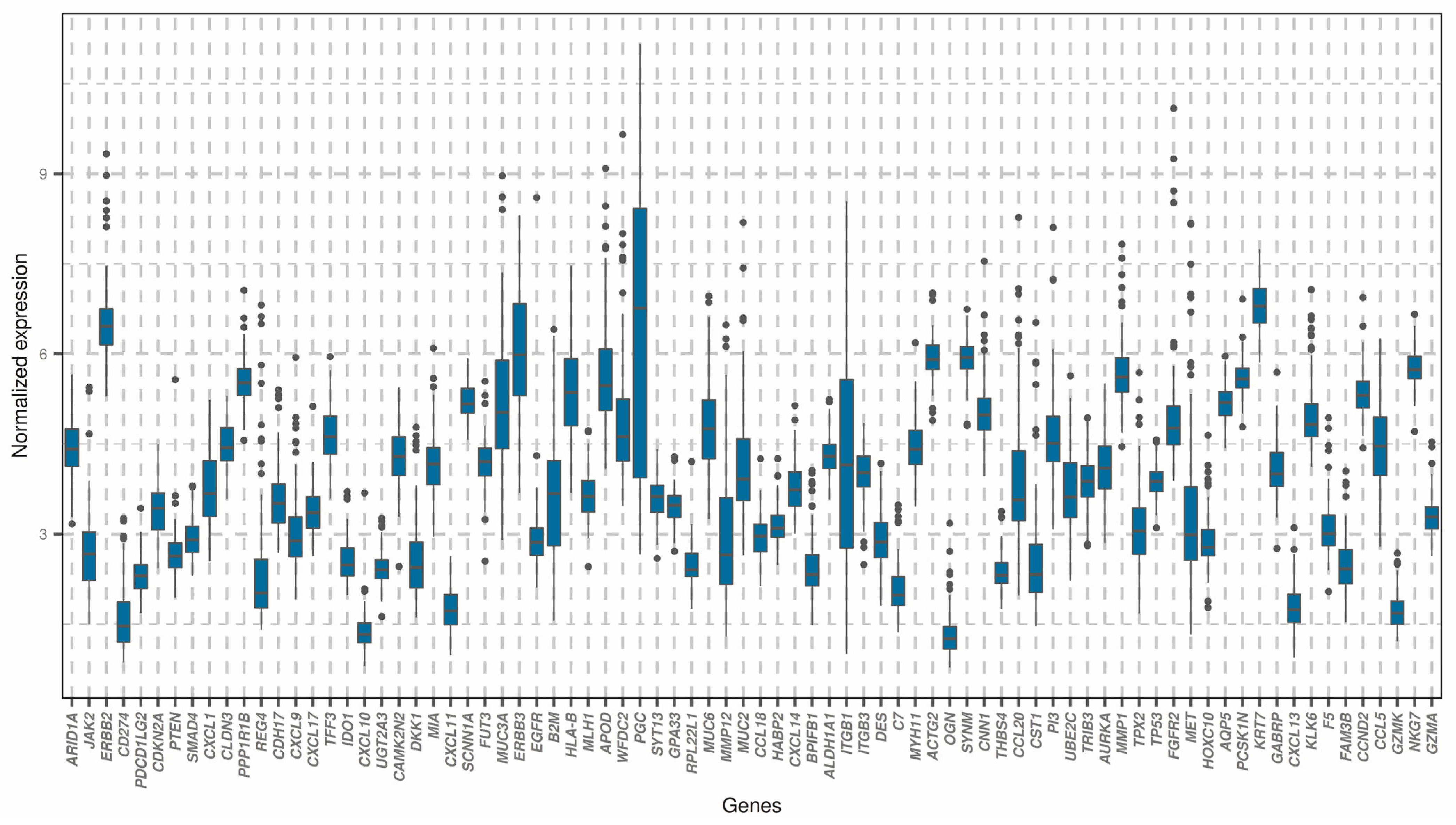

Supplement: Supplementary file 1 [file biomedicines-10-00148-s001.zip › biomedicines-1533227-supplementary/Figure S1.tiff]
